# Supplementary material for: CAM photosynthesis may have conferred an advantage during the Permian–Triassic mass extinction event
Source: Nat Ecol Evol. 2026 Apr 20;10(5):997–1010. doi: 10.1038/s41559-026-03026-0 (PMC13167456; doi:10.1038/s41559-026-03026-0)
Supplement: Supplementary file 1 — Supplementary Figs. 1–32, Tables 1–5, R code 1–3, and text with an explanation of morphological characters and figures. [file 41559_2026_3026_MOESM1_ESM.pdf]

nature ecology & evolution

Supplementary information

https://doi.org/10.1038/s41559-026-03026-0

CAM photosynthesis may have conferred an advantage during the Permian–Triassic mass extinction event

In the format provided by the authors and unedited

Supplementary Information 3

This file includes:

Supplementary text for the morphological character explanation with figures
Figs. S1–S32
Table S1–S5
R code 1–3

Other Supplementary Materials for this manuscript include the following:

Character Matrix named as Additional_supplementary_information_1-morphological_characte_matrix

Supplementary text: Morphological character explanation with figures

Root:

[Ch-1] Root *Stigmaria*: Absent (0); Present (1).

[Ch-2] Root *Protostigmaria*: Absent (0); Present (1).

Whole plant morphology habit:

[Ch-3] Herbaceous: Absent (0); Present (1).

[Ch-4] Shrub: Absent (0); Present (1).

[Ch-5] Arborescent: Absent (0); Present (1).

Cone:

[Ch-6] Single cone on each plant: Absent (0); Present (1).

[Ch-7] Multiple cones on each plant: Absent (0); Present (1).

[Ch-8] Cone positioned at shoot apex: Absent (0); Present (1).

[Ch-9] Cone positioned at end of axis: Absent (0); Present (1).

[Ch-10] Single sporangium positioned on leaf or attached to branch: Absent (0); Present (1).

Annotation: [C8] 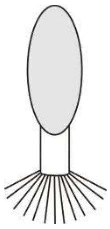 [C9] 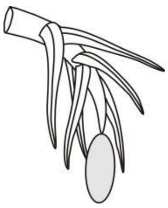 [C10]: 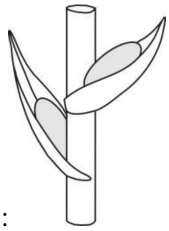

Sporophyll phylotaxy in cone:

[Ch-11] Sporophylls arranged in cluster: Absent (0); Present (1).

[Ch-12] Sporophyll spiral or colyliform: Absent (0); Present (1).

Annotation: [12] Lateral view spiral sporophyll 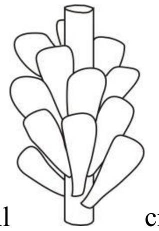 cross section of spiral cone

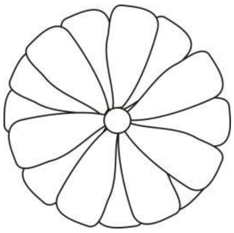

Lateral view colyliform sporophylls 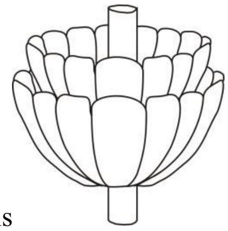 cross section of colyliform sporophylls

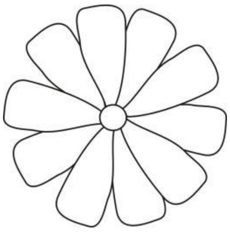

[Ch-13] Sporophyll dispersed through plant: Absent (0); Present (1).

Sexual reproduction and spore type:

[Ch-14] Homosporous: Absent (0); Present (1).

[Ch-15] Heterosporous: Absent (0); Present (1).

[Ch-16] Megaspore number per megasporangium: Multiple (0); Single (1).

Leaf morphology:

[Ch-17] Fertile and vegetative leaves different shape: Absent (0); Present (1).

Sporophyll (fertile leaf):

[Ch-18] Sporophyll planar (without transverse ridge): Absent (0); Present (1).

Annotation: [Ch-18] with transverse ridge 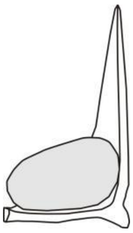 without transverse ridge 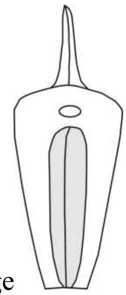

Sporophyll shape:

[Ch-19] Sporophyll oval-rounded to rectangular: Absent (0); Present (1).

[Ch-20] Sporophyll triangular: Absent (0); Present (1).

[Ch-21] Sporophyll ligulate: Absent (0); Present (1).

[Ch-22] Sporophyll reverse taper: Absent (0); Present (1).

[Ch-23] Sporophyll linear: Absent (0); Present (1).

Annotation:

[Ch-19] 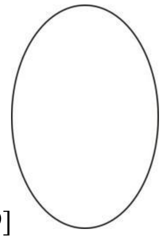 [Ch-20] 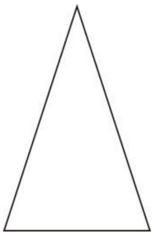 [Ch-21] 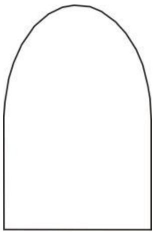 [Ch-22] 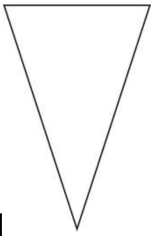 [Ch-23]

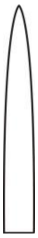

Sporophyll area/square area (FS):

[Ch-24] FS>π/4 (0.79): Absent (0); Present (1).

[Ch-25] FS≈0.5: Absent (0); Present (1).

[Ch-26] 0.5<FS≦π/4 (0.5-0.79): Absent (0); Present (1).

[Ch-27] FS<0.5: Absent (0); Present (1).

Annotation:

[Ch-24] 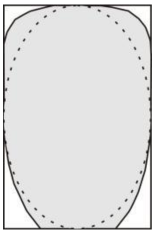 [Ch-25] 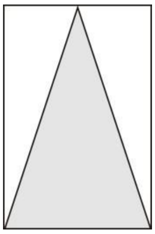 [Ch-26] 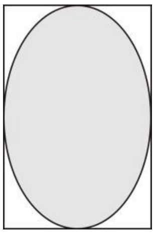 [Ch-27] 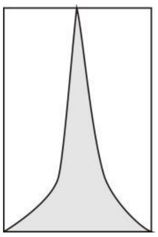

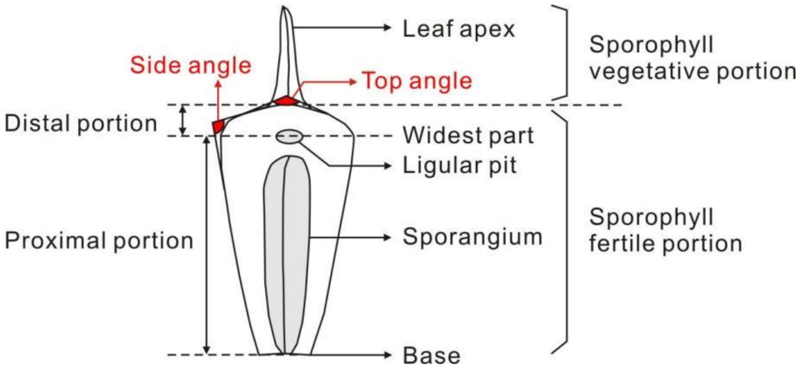

Figure S1. Sporophyll character definition summary

Sporophyll Fertile Portion length (FP):

[Ch-28] 0<FP≦10mm: Absent (0); Present (1).

[Ch-29] 10<FP≦20mm: Absent (0); Present (1).

[Ch-30] 20<FP≦30mm: Absent (0); Present (1).

[Ch-31] 30<FP≦40mm: Absent (0); Present (1).

[Ch-32] 40<FP≦50mm: Absent (0); Present (1).

[Ch-33] FP>50mm: Absent (0); Present (1).

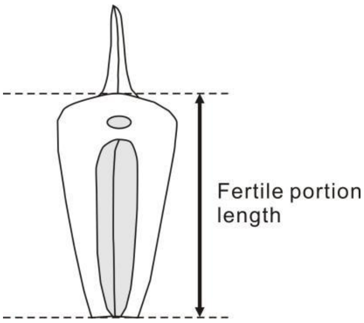

Annotation: [Ch-28]–[Ch-33]

Sporophyll Maximum Width (MW):

[Ch-34] 0<MW≦5mm: Absent (0); Present (1).

[Ch-35] 5<MW≦10mm: Absent (0); Present (1).

[Ch-36] 10<MW≦15mm: Absent (0); Present (1).

[Ch-37] 15<MW≦20mm: Absent (0); Present (1).

[Ch-38] 20<MW≦25mm: Absent (0); Present (1).

[Ch-39] 25<MW≦30mm: Absent (0); Present (1).

[Ch-40] MW>30mm: Absent (0); Present (1).

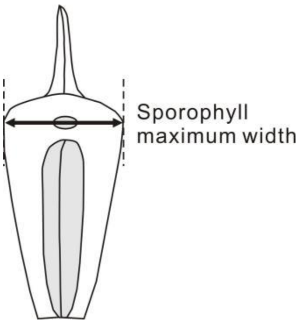

Annotation: [Ch-34]–[Ch-40]

Sporophyll Fertile Portion length/ maximum width (FM):

[Ch-41] 0<FM≦1: Absent (0); Present (1).

[Ch-42] 1<FM≦2: Absent (0); Present (1).

[Ch-43] 2<FM≦3: Absent (0); Present (1).

[Ch-44] 3<FM≦6: Absent (0); Present (1).

Area (including fertile portion and sporophyll apex):

[Ch-45] 0<Area≦50mm^2: Absent (0); Present (1).

[Ch-46] 50<Area≦100mm^2: Absent (0); Present (1).

[Ch-47] 100<Area≦150mm^2: Absent (0); Present (1)

[Ch-48] 150<Area≦200mm^2: Absent (0); Present (1).

[Ch-49] 200<Area≦250mm^2: Absent (0); Present (1).

[Ch-50] 250<Area≦500mm^2: Absent (0); Present (1).

[Ch-51] 500<Area≦1000mm^2: Absent (0); Present (1).

[Ch-52] Area>1000mm^2: Absent (0); Present (1).

Ligular pit:

[Ch-53] Ligular pit: Absent (0); Present (1).

[Ch-54] Ligular pit position at the top of sporophyll fertile portion: Absent (0); Present (1).
[Ch-55] Ligular pit position at 1/2–2/3 of sporophyll fertile portion: Absent (0); Present (1).
[Ch-56] Ligular pit position at 1/3–1/2 of the sporophyll fertile portion: Absent (0); Present (1).
[Ch-57] Ligular pit position at <1/3 of the sporophyll fertile portion: Absent (0); Present (1).

Annotation: [Ch-54]

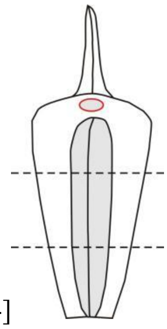

[Ch-55]

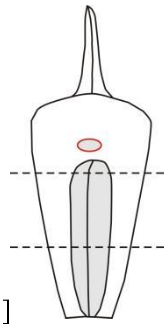

[Ch-56]

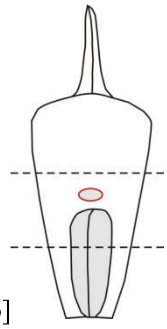

[Ch-57]

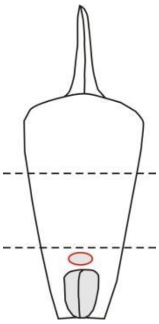

Sporophyll apex: [Ch-58] leaf apex (sterile portion): Absent (0); Present (1).

Width of sporophyll apex widest part:
[Ch-59] Sporophyll apex widest part <<Sporophyll fertile portion width: Absent (0); Present (1).
[Ch-60] Sporophyll apex widest part equal to sporophyll fertile length: Absent (0); Present (1).

Total sporophyll length/sporophyll fertile portion length (TS):
[Ch-61] TS≦1: Absent (0); Present (1).
[Ch-62] 1<TS≦2: Absent (0); Present (1).
[Ch-63] 2<TS≦5: Absent (0); Present (1).
[Ch-64] TS > 5: Absent (0); Present (1).

Sporophyll distal portion (DP):
[Ch-65] DP at 1-2/3 of the fertile portion: Absent (0); Present (1).
[Ch-66] DP at 2/3-1/2 of the fertile portion: Absent (0); Present (1).
[Ch-67] DP at 1/2-1/3 of the fertile portion: Absent (0); Present (1).
[Ch-68] DP at <1/3 of the fertile portion: Absent (0); Present (1).

Annotation: [Ch-65]

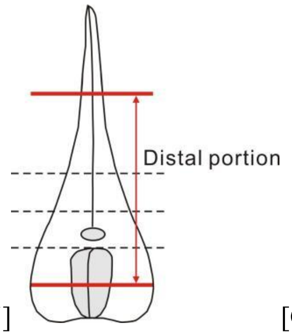

[Ch-66]

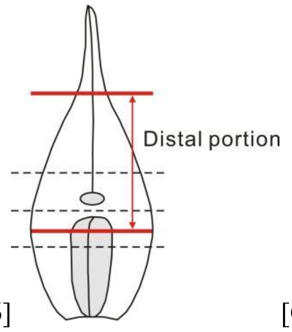

[Ch-67]

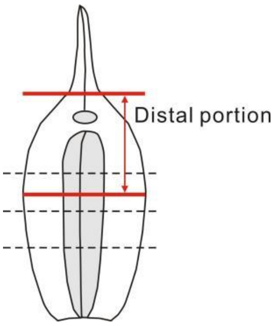

[Ch-68]

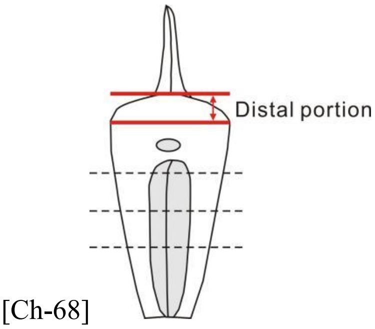

Sporophyll apical angle:

[Ch-69] Apical angle ≈180°: Absent (0); Present (1).

[Ch-70] 180°<Apical angle≦90°: Absent (0); Present (1).

[Ch-71] Apical angle <90°: Absent (0); Present (1).

Annotation: [Ch-69]

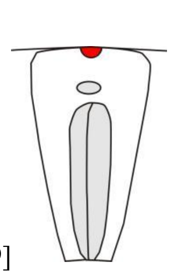

[Ch-70]

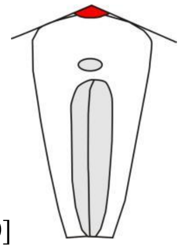

[Ch-71]

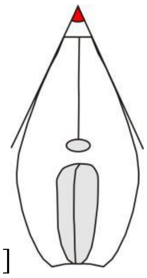

Sporophyll apex angle of the widest part (AW):

[Ch-72] AW≦90°: Absent (0); Present (1).

[Ch-73] 90°<AW≦120°: Absent (0); Present (1).

[Ch-74] 120°<AW≦180°: Absent (0); Present (1).

Annotation: [Ch-72]

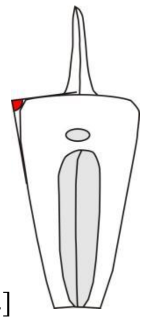

[Ch-73]

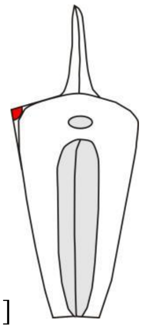

[Ch-74]

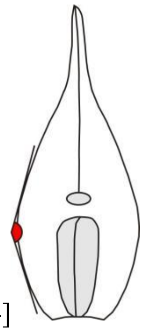

Sporophyll Proximal portion morphology:

[Ch-75] Gradual narrow to the base: Absent (0); Present (1).

[Ch-76] Concave: Absent (0); Present (1).

Annotation: [Ch-75] 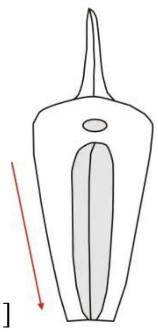 [Ch-76] 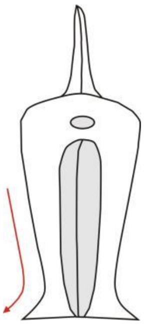

Sporophyll Base

[Ch-77] Petiole base: Absent (0); Present (1).

[Ch-78] Lobed base: Absent (0); Present (1).

[Ch-79] Flat base: Absent (0); Present (1).

[Ch-80] Hastate base: Absent (0); Present (1).

Annotation:

[Ch-77] 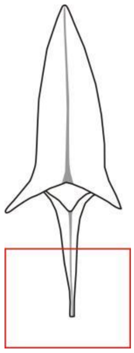 [Ch-78] 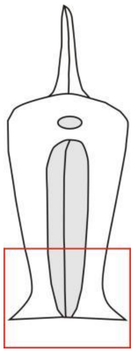 [Ch-79] 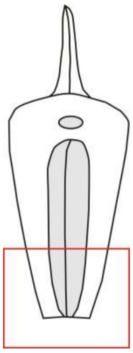 [Ch-80] 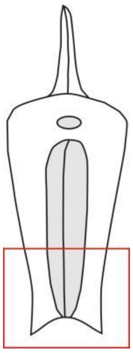

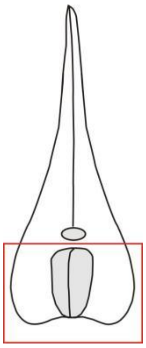

Sporophyll surface structures

[Ch-81] Longitudinal vein through whole sporophyll: Absent (0); Present (1).

[Ch-82] Longitudinal vein in leaf apex: Absent (0); Present (1).

[Ch-83] Transverse partition (wrinkle) on sporophyll: Absent (0); Present (1).

[Ch-84] Air bladder on sporophyll: Absent (0); Present (1).

[Ch-85] Multiple longitudinal grooves on sporophyll: Absent (0); Present (1).

[Ch-86] Sporophyll margin entire or nearly so: Absent (0); Present (1).
[C87] Sporophyll margin with lobe: Absent (0); Present (1).

Annotation:

[Ch-81]
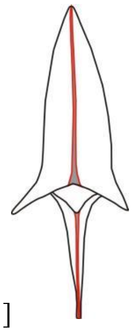
[Ch-82]
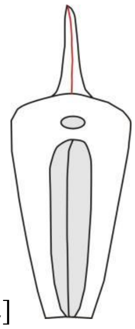
[Ch-83]
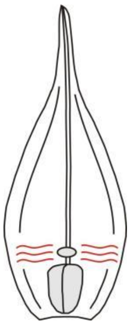
[Ch-84]
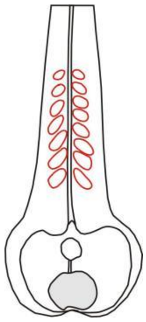
[Ch-85]
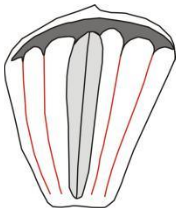

Sporangium (fertile portion):
[Ch-88] Clavate sporangium shape: Absent (0); Present (1).
[Ch-89] Round to oval sporangium: Absent (0); Present (1).

Annotation: [Ch-88]
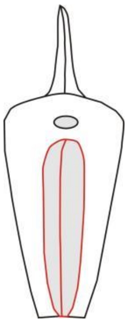
[Ch-89]
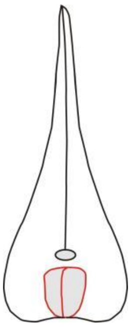

Sporangium position:
[Ch-90] Sporangium proximal on sporophyll length: Absent (0); Present (1).
[Ch-91] Sporangium medial on sporophyll length: Absent (0); Present (1).

Annotation: [Ch-90]
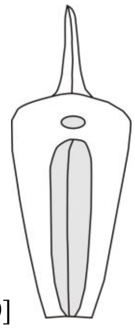
[Ch-91]
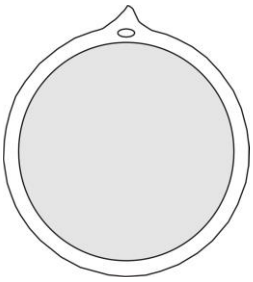

Sporangium Length (SL):

[Ch-92] 0<SL≦5mm: Absent (0); Present (1).
[Ch-93] 5<SL≦10mm: Absent (0); Present (1).
[Ch-94] 10<SL≦15mm: Absent (0); Present (1).
[Ch-95] 15<SL≦20mm: Absent (0); Present (1).
[Ch-96] 20<SL≦30mm: Absent (0); Present (1).
[Ch-97] 30<SL≦50mm: Absent (0); Present (1).

Annotation: [Ch-92]–[Ch-97]

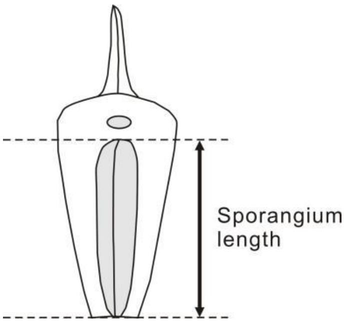

Sporangium Width (SW):
[Ch-98] 0<SW≦2mm: Absent (0); Present (1).
[Ch-99] 2<SW≦4mm: Absent (0); Present (1).
[Ch-100] 4<SW≦10mm: Absent (0); Present (1).
[Ch-101] 10<SW≦40mm: Absent (0); Present (1).

Annotation: [Ch-98]–[Ch-101]

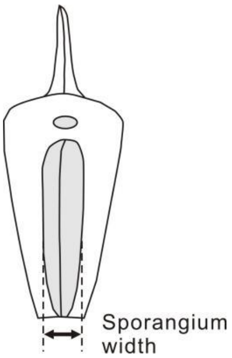

Sporangium area (SA):
[Ch-102] 0<SA≦20mm^2: Absent (0); Present (1).
[Ch-103] 20<SA≦40mm^2: Absent (0); Present (1).
[Ch-104] 40<SA≦60mm^2: Absent (0); Present (1).
[Ch-105] 60<SA≦80mm^2: Absent (0); Present (1).
[Ch-106] 80<SA≦100mm^2: Absent (0); Present (1).
[Ch-107] 100<SA≦200mm^2: Absent (0); Present (1).
[Ch-108] 200<SA≦400mm^2: Absent (0); Present (1).
[Ch-109] 400<SA≦1200mm^2: Absent (0); Present (1).

Sporangium Surface:
[Ch-110] Sporangium smooth surface: Absent (0); Present (1).

[Ch-111] Sporangium with one mid groove: Absent (0); Present (1).

[Ch-112] Sporangium with multiple longitudinal rib: Absent (0); Present (1).

Annotation: [Ch-110] 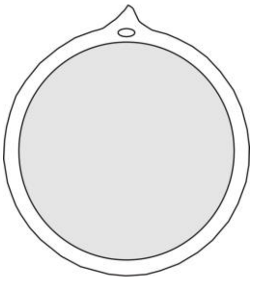 [Ch-111] 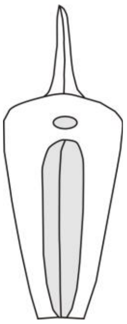 [Ch-112] 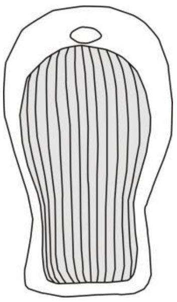

Sporophyll area/Sporangium area (SAS):

[Ch-113] 0<SAS≦2: Absent (0); Present (1).

[Ch-114] 2<SAS≦5: Absent (0); Present (1).

[Ch-115] 5<SAS≦10: Absent (0); Present (1).

[Ch-116] 10<SAS≦45: Absent (0); Present (1).

Sporophyll fertile portion length/sporangium length (SLS):

[Ch-117] SLS≦1: Absent (0); Present (1).

[Ch-118] 1<SLS≦2: Absent (0); Present (1).

[Ch-119] 2<SLS≦3: Absent (0); Present (1).

[Ch-120] 3<SLS≦4: Absent (0); Present (1).

[Ch-121] SLS>4: Absent (0); Present (1).

Sporophyll maximum width/sporangium width (SWS):

[Ch-122] SWS≦1: Absent (0); Present (1).

[Ch-123] 1<SWS≦2: Absent (0); Present (1).

[Ch-124] 2<SWS≦3: Absent (0); Present (1).

[Ch-125] 3<SWS≦4: Absent (0); Present (1).

[Ch-126] 4<SWS≦5: Absent (0); Present (1).

[Ch-127] SWS>5: Absent (0); Present (1).

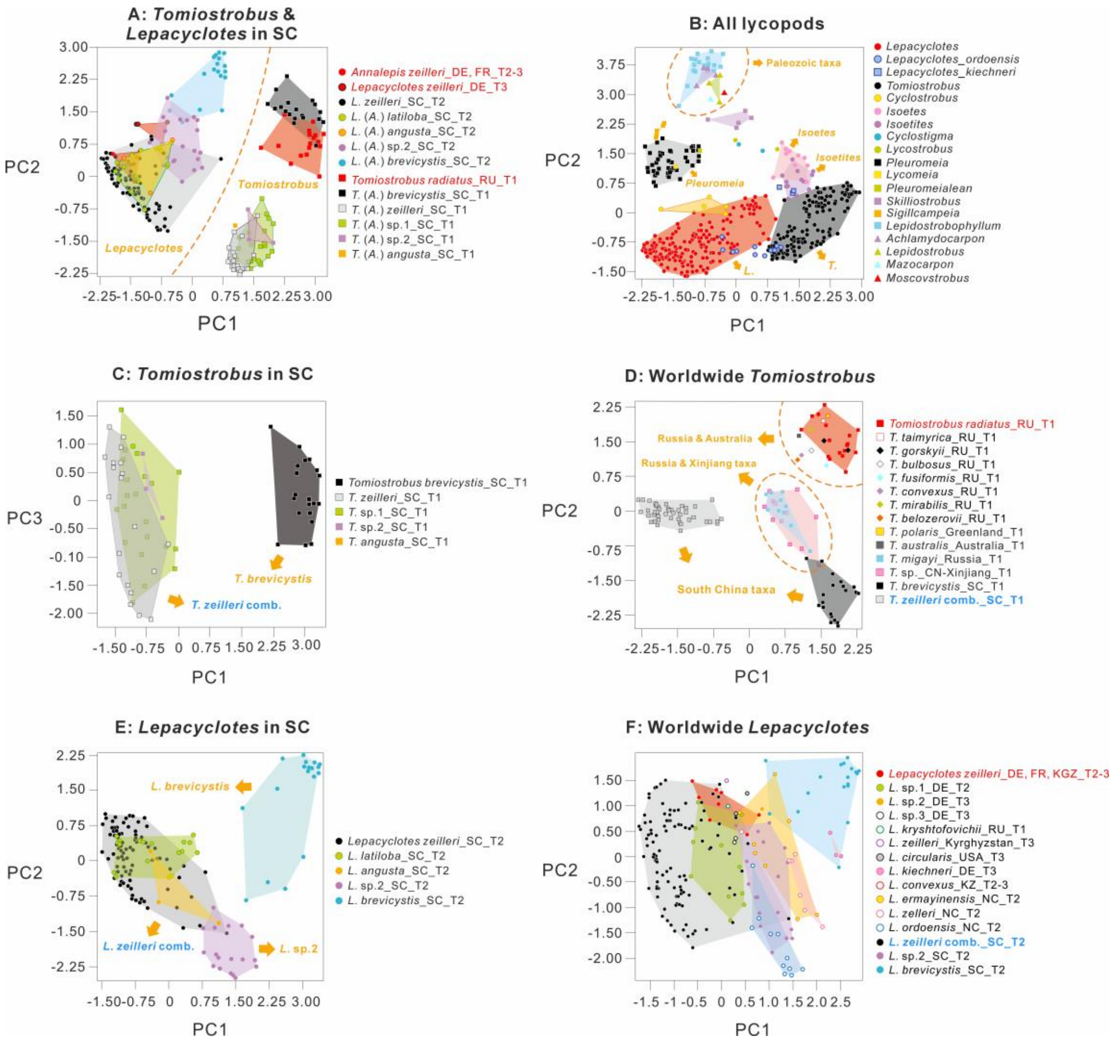

Fig. S1: Supplementary two-dimensional PCA results of Figure 2.

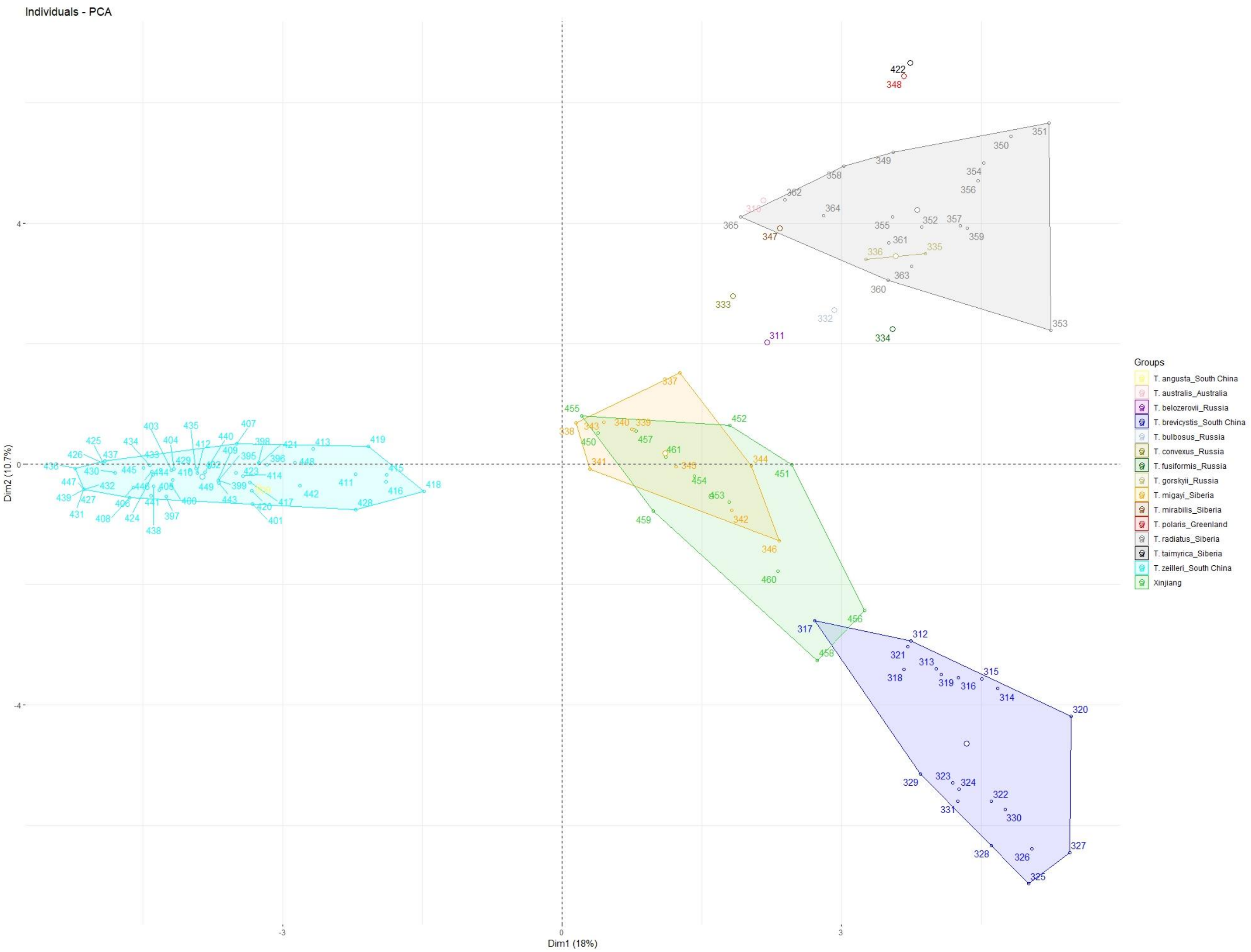
Individuals - PCA
Dim1 (18%)
Dim2 (10.7%)
-3
0
3
4
0
-4
Groups
T. angusta_South China
T. australis_Australia
T. belozerovii_Russia
T. brevicystis_South China
T. bulbosus_Russia
T. convexus_Russia
T. fusiformis_Russia
T. gorskyii_Russia
T. migayi_Siberia
T. mirabilis_Siberia
T. polaris_Greenland
T. radiatus_Siberia
T. taimyrica_Siberia
T. zeilleri_South China
Xinjiang

Fig. S2: Two-dimensional PCA results of the worldwide *Tomiostrobus* species sporophylls showing centroids of each PCA group. The centroids are virtual dots that are larger than other sample datapoints. The sample datapoints closest to the virtual centroids are used in the phylogenic neighborhood network analysis. Find the chosen datapoints in the Supplementary Information 2 that highlighted with a check mark. For high-resolution vector versions of Figures S2 and S3, please run the R code provided at the end of this file using the original data in Additional supplementary file 1. Note that, for each figure, the corresponding data in Additional supplementary file 1 must be appropriately filtered.

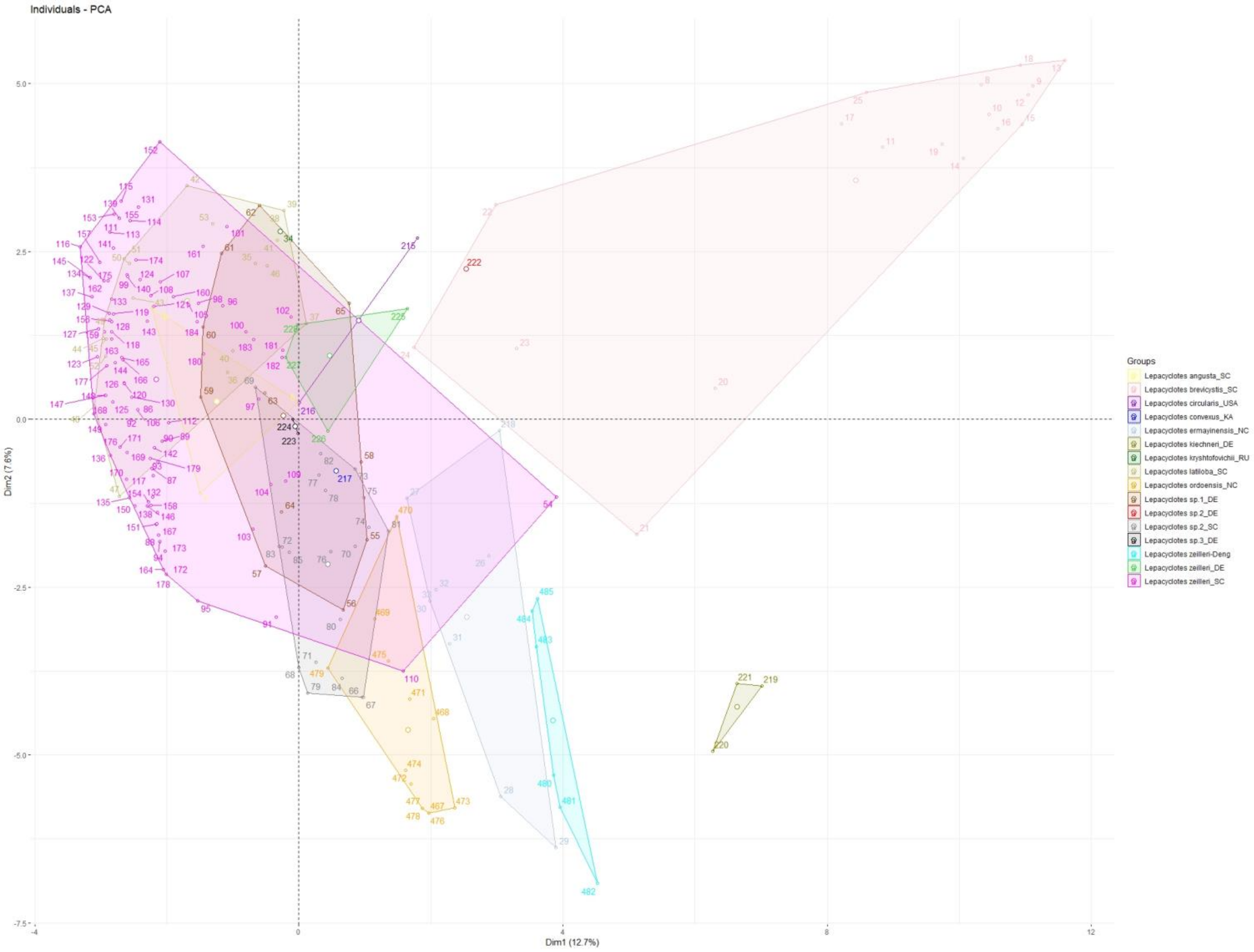
Individuals - PCA
Dim1 (12.7%)
Dim2 (7.6%)
Groups
Lepacyclotes angusta_SC
Lepacyclotes brevicystis_SC
Lepacyclotes circularis_USA
Lepacyclotes convexus_KA
Lepacyclotes ermayinensis_NC
Lepacyclotes kiechneri_DE
Lepacyclotes kryshtofovichii_RU
Lepacyclotes latiloba_SC
Lepacyclotes ordoensis_NC
Lepacyclotes sp.1_DE
Lepacyclotes sp.2_DE
Lepacyclotes sp.2_SC
Lepacyclotes sp.3_DE
Lepacyclotes zeilleri-Deng
Lepacyclotes zeilleri_DE
Lepacyclotes zeilleri_SC

Fig. S3: Two-dimensional PCA results of the worldwide *Lepacyclotes* species sporophylls showing centroids of each PCA group. The centroids are virtual dots that are larger than other sample datapoints. The sample datapoints closest to the virtual centroids are used in the phylogenic neighborhood network analysis. Find the chosen datapoints in the Supplementary Information 2 that highlighted with a check mark. For high-resolution vector versions of Figures S2 and S3, please run the R code provided at the end of this file using the original data in Additional supplementary file 1. Note that, for each figure, the corresponding data in Additional supplementary file 1 must be appropriately filtered.

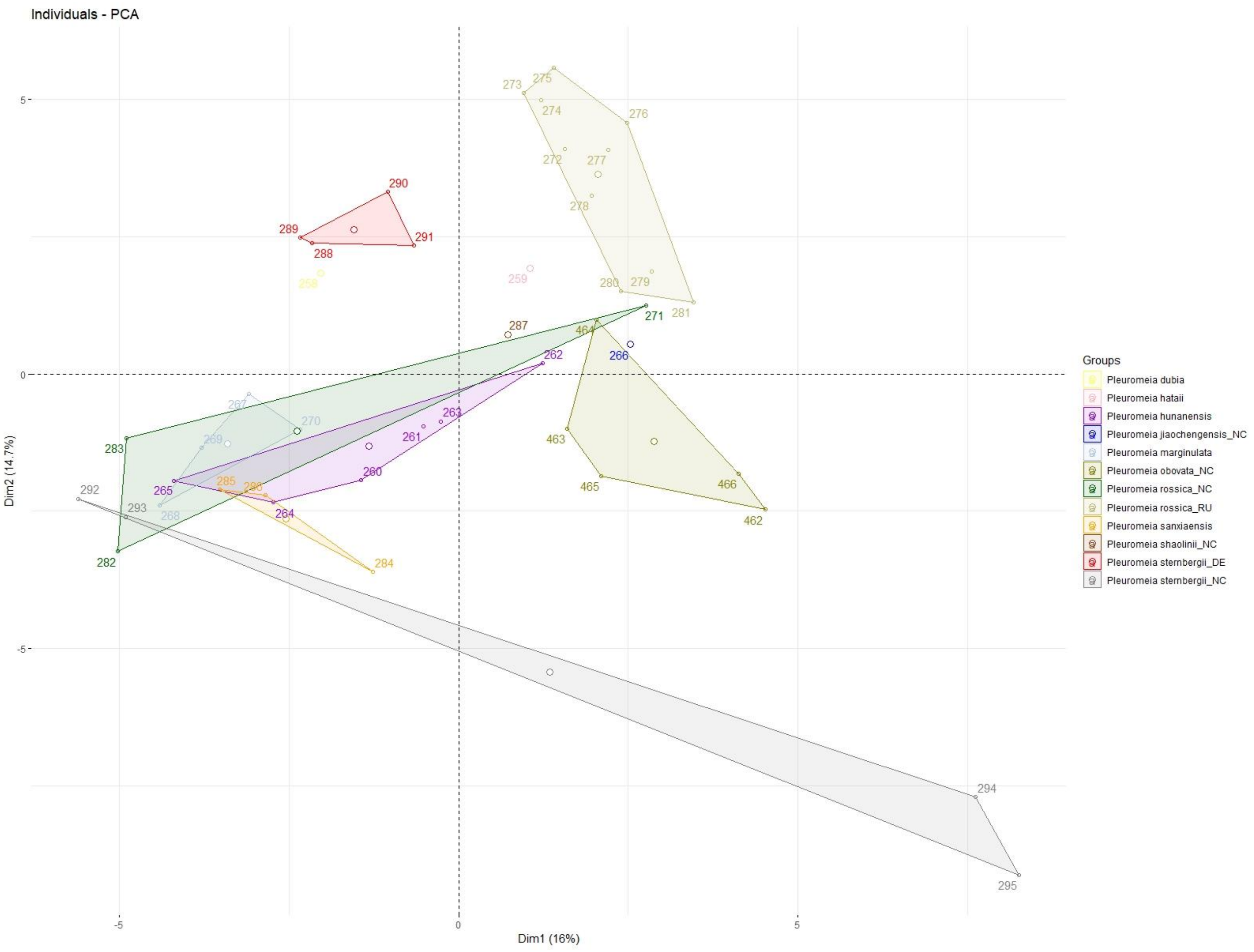
Individuals - PCA
Dim1 (16%)
Dim2 (14.7%)
Groups
Pleuromeia dubia
Pleuromeia hataii
Pleuromeia hunanensis
Pleuromeia jiaochengensis_NC
Pleuromeia marginulata
Pleuromeia obovata_NC
Pleuromeia rossica_NC
Pleuromeia rossica_RU
Pleuromeia sanxiaensis
Pleuromeia shaolinii_NC
Pleuromeia sternbergii_DE
Pleuromeia sternbergii_NC

Fig. S4: Two-dimensional PCA results of the worldwide *Pleuromeia* species sporophylls showing centroids of each PCA group. The centroids are virtual dots that are larger than other sample datapoints. The sample datapoints closest to the virtual centroids are used in the phylogenic neighborhood network analysis. Find the chosen datapoints in the Additional supplementary file 1 that highlighted with a check mark.

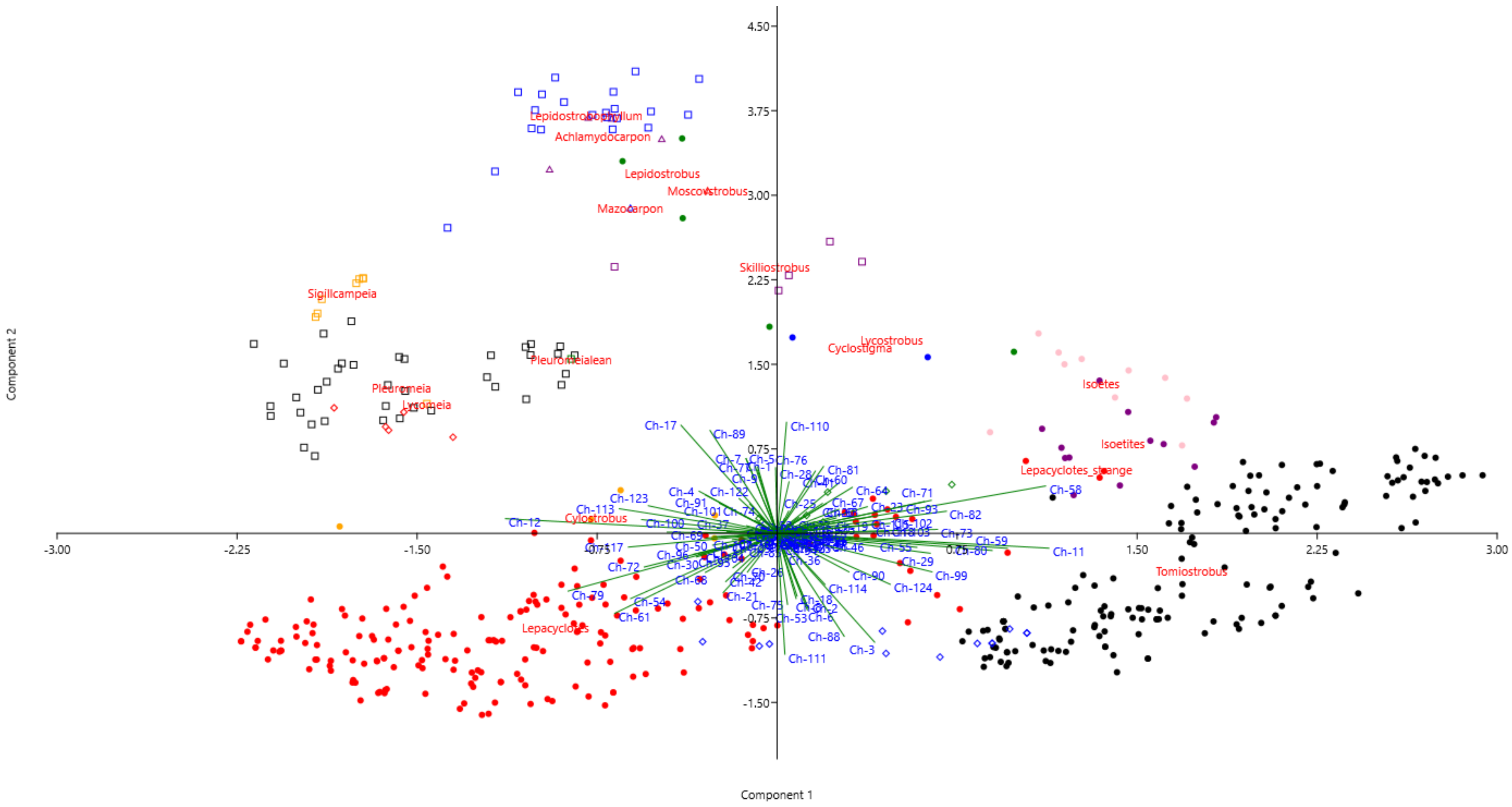

Fig. S5: Biplot of the figure 2B. This figure shows the contribution of each character on the PC1 and PC2. See the detail contribution of each character in the table S3.

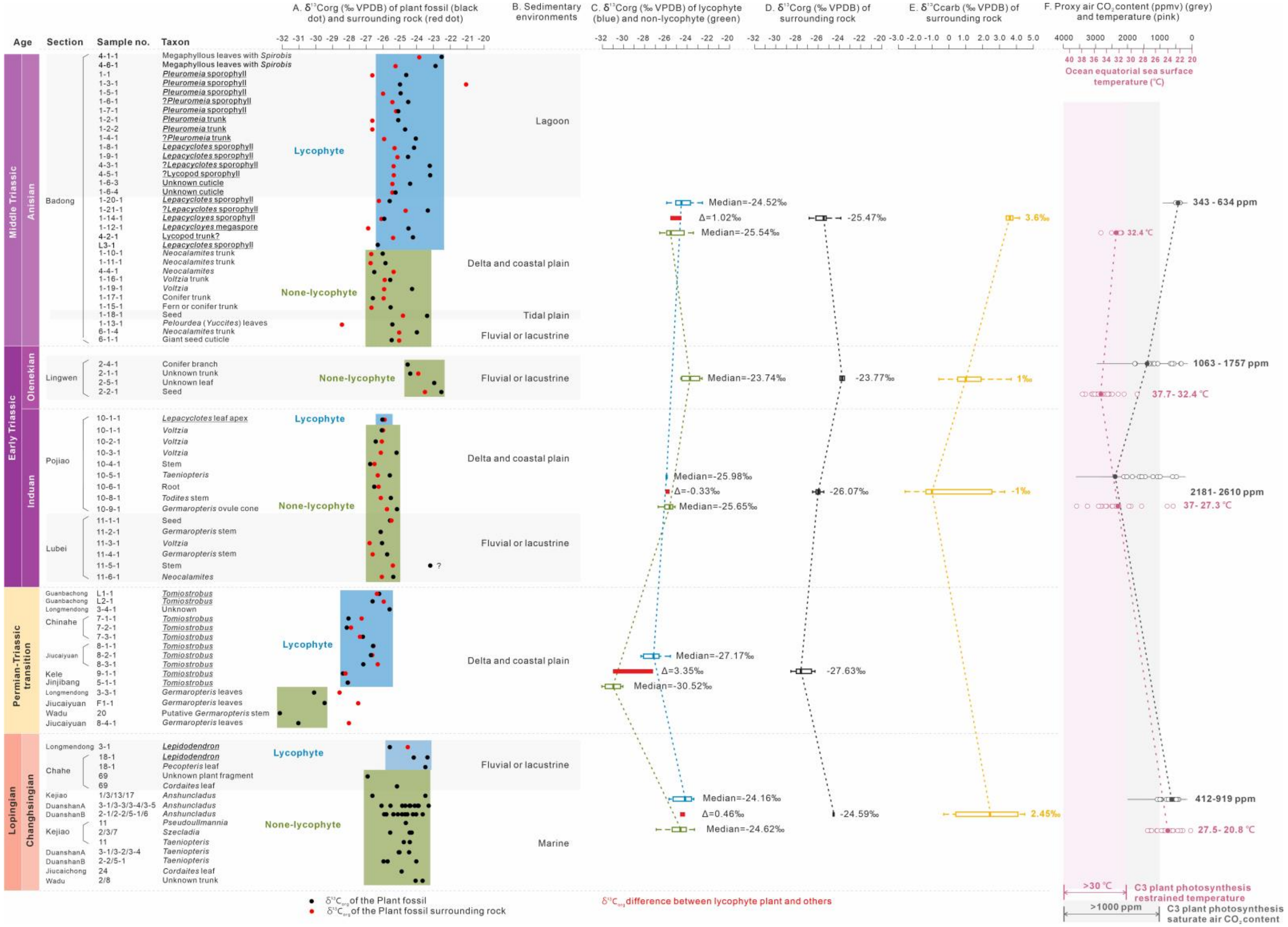

A. δ13Corg (‰ VPDB) of plant fossil (black dot) and surrounding rock (red dot)
B. Sedimentary environments
C. δ13Corg (‰ VPDB) of lycophyte (blue) and non-lycophyte (green)
D. δ13Corg (‰ VPDB) of surrounding rock
E. δ13Ccarb (‰ VPDB) of surrounding rock
F. Proxy air CO2 content (ppmv) (grey) and temperature (pink)
Age
Section
Sample no.
Taxon
Ocean equatorial sea surface temperature (℃)
Middle Triassic
Anisian
Early Triassic
Olenekian
Induan
Permian-Triassic transition
Lopingian
Changhsingian
Badong
Lingwen
Pojiao
Lubei
Chahe
Lycophyte
None-lycophyte
Lagoon
Delta and coastal plain
Tidal plain
Fluvial or lacustrine
Marine
Median=-24.52‰
Δ=1.02‰
Median=-25.54‰
-25.47‰
3.6‰
32.4 ℃
343 - 634 ppm
Median=-23.74‰
-23.77‰
1‰
1063 - 1757 ppm
37.7- 32.4 ℃
Median=-25.98‰
Δ=-0.33‰
Median=-25.65‰
-26.07‰
-1‰
2181- 2610 ppm
37- 27.3 ℃
Median=-27.17‰
Δ=3.35‰
Median=-30.52‰
-27.63‰
Median=-24.16‰
Δ=0.46‰
Median=-24.62‰
-24.59‰
2.45‰
412-919 ppm
27.5- 20.8 ℃
>30 ℃
C3 plant photosynthesis restrained temperature
>1000 ppm
C3 plant photosynthesis saturate air CO2 content
δ13Corg of the Plant fossil
δ13Corg of the Plant fossil surrounding rock
δ13Corg difference between lycophyte plant and others

Fig. S6: Section name, number taxa information, sedimentary environment of samples, together with the organic carbon isotope, carbonate inorganic carbon isotope, proxy equatorial sea surface temperature and atmospheric CO_2 reconstructions from end Permian to Middle Triassic in South China. The late Permian plant fossils were of the late Changhsingian age and yield in few beds at the upper part of the Xuanwei Formation which could be found in Li et al.[17] and Xu et al.[5, 77]. The Permian-Triassic transition plant fossils yield in one bed at the bottom of the Kayitou Formation and the age is correlated by the herbaceous lycopod *Tomiostrobus* and spinicaudata in Yu et al.[6] and Chu et al.[78]. The Early Triassic plant fossils were of the Olenekian age at the lower part of the Lingwen Formation and further information could be found in Zhou[79] and Xu et al.[5, 77]. The Middle Triassic plant fossils were of Anisian age at the bottom of the Badong Formation and more details could be found in Meng et al.[30] and Xu et al.[5, 77]. All the section information could be found in Xu et al.[5, 77]. All the plant fossil sampling pictures could be found in the supplementary files according to the specimen number in the front of the taxon name. The underlined plant taxa are lycopods. The black dots are the organic carbon isotope value of the plant fossil, and the red dots were the organic carbon isotope value of the surrounding rock at the same layer with the plant fossil. The black and red dots of each sample are in the same line. The blue shadow squares are putative "mangrove-like" plant and the green shadow shows the lowland-upland plant taxa. The sedimentary facies information of each plant fossils come from Xu et al.[5, 77]. The median organic carbon isotope values of the "mangrove-like" plant (blue) and the contemporaneous lowland-upland plants (green) of each period are listed next to the sedimentary information. The difference between the contemporaneous carbon isotope of the "mangrove-like" (blue dots and blue dash curve) and the lowland-upland (green dots and green dash curve) plants is marked as red thick line. The Early Triassic lacked lycopods data. The reconstructed atmospheric atmospheric CO_2 content data of each period comes from the plant stomata and paleosol data in Joachimski et al.[4] and is shown as grey dots and dash curve. The atmospheric CO_2 content data (grey) is slightly above the temperature data (pink) for no covering and these two sets of data come from the same period. Recent C_3 plant photosynthesis is generally saturated with atmospheric CO_2 content over 1000ppm and is shown in the gray square. The reconstructed equatorial sea surface temperature comes from Sun et al.[16] and is shown as pink dots and dash curve. Recent C_3 plant photosynthesis process is restrained when land surface temperature exceeded 40°C which is roughly equal to the sea surface temperature >30°C and is shown in the pink square. The time frameworks in all the cited data are correlated by the chronostratigraphy with the CTS2020. Because all the plant fossils in one time period are considered as contemporaneous, the median isotope, atmospheric CO_2 content and equatorial sea surface temperature are listed in the middle of each period.

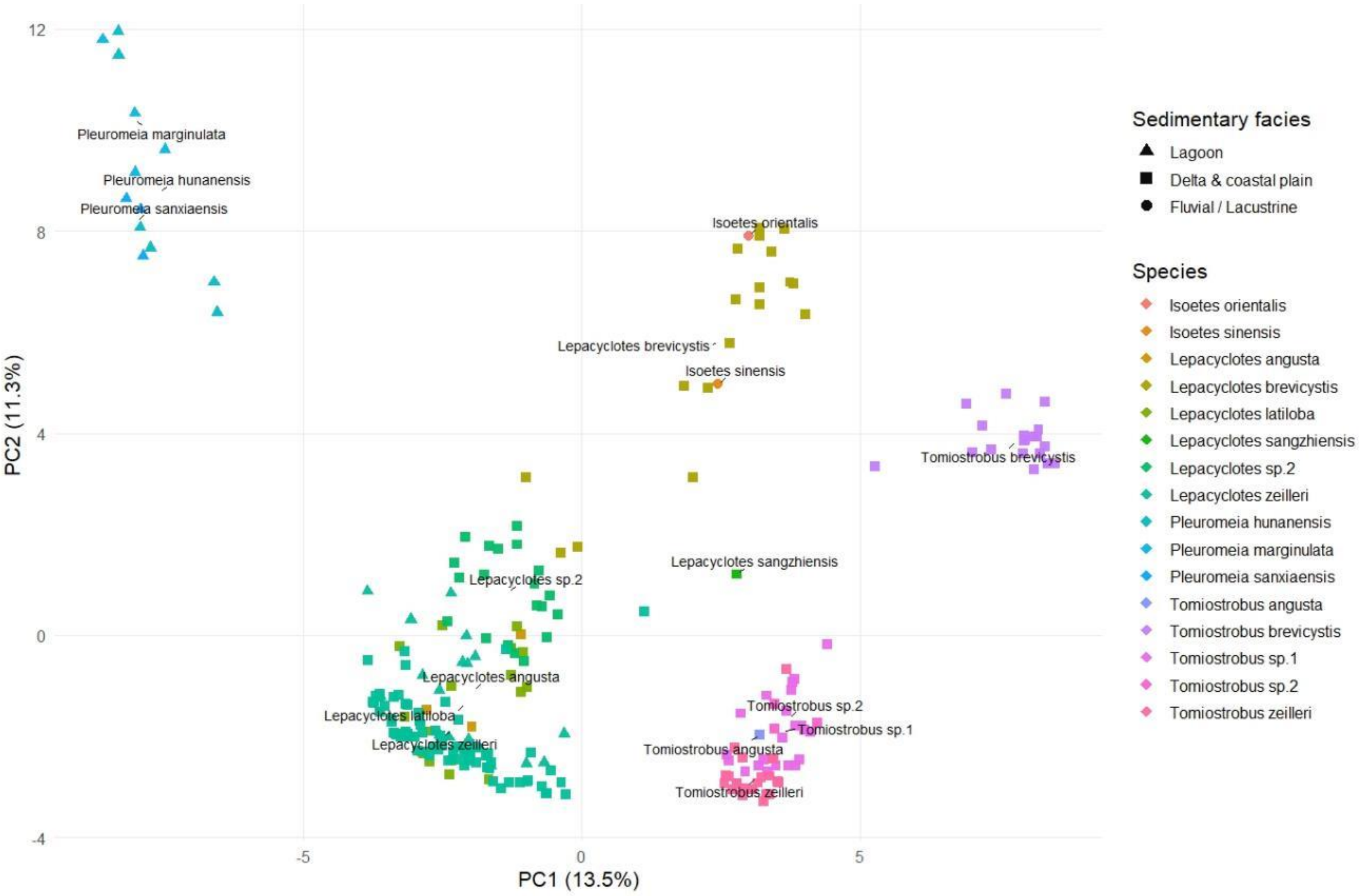

PC1 (13.5%)
PC2 (11.3%)
Sedimentary facies
Lagoon
Delta & coastal plain
Fluvial / Lacustrine
Species
Isoetes orientalis
Isoetes sinensis
Lepacyclotes angusta
Lepacyclotes brevicystis
Lepacyclotes latiloba
Lepacyclotes sangzhiensis
Lepacyclotes sp.2
Lepacyclotes zeilleri
Pleuromeia hunanensis
Pleuromeia marginulata
Pleuromeia sanxiaensis
Tomiostrobus angusta
Tomiostrobus brevicystis
Tomiostrobus sp.1
Tomiostrobus sp.2
Tomiostrobus zeilleri

Figure S7. Two-dimensional PCA of South China lycophyte sporophyll morphometrics (Triassic–Recent), evaluated against sedimentary facies. Sedimentary facies are represented by different symbol shapes, while species are represented by different colors. R code 2 for producing this figure is attached in this file. The result indicates that sedimentary facies/taphonomy is not a primary determinant of taxonomic grouping in ordination space.

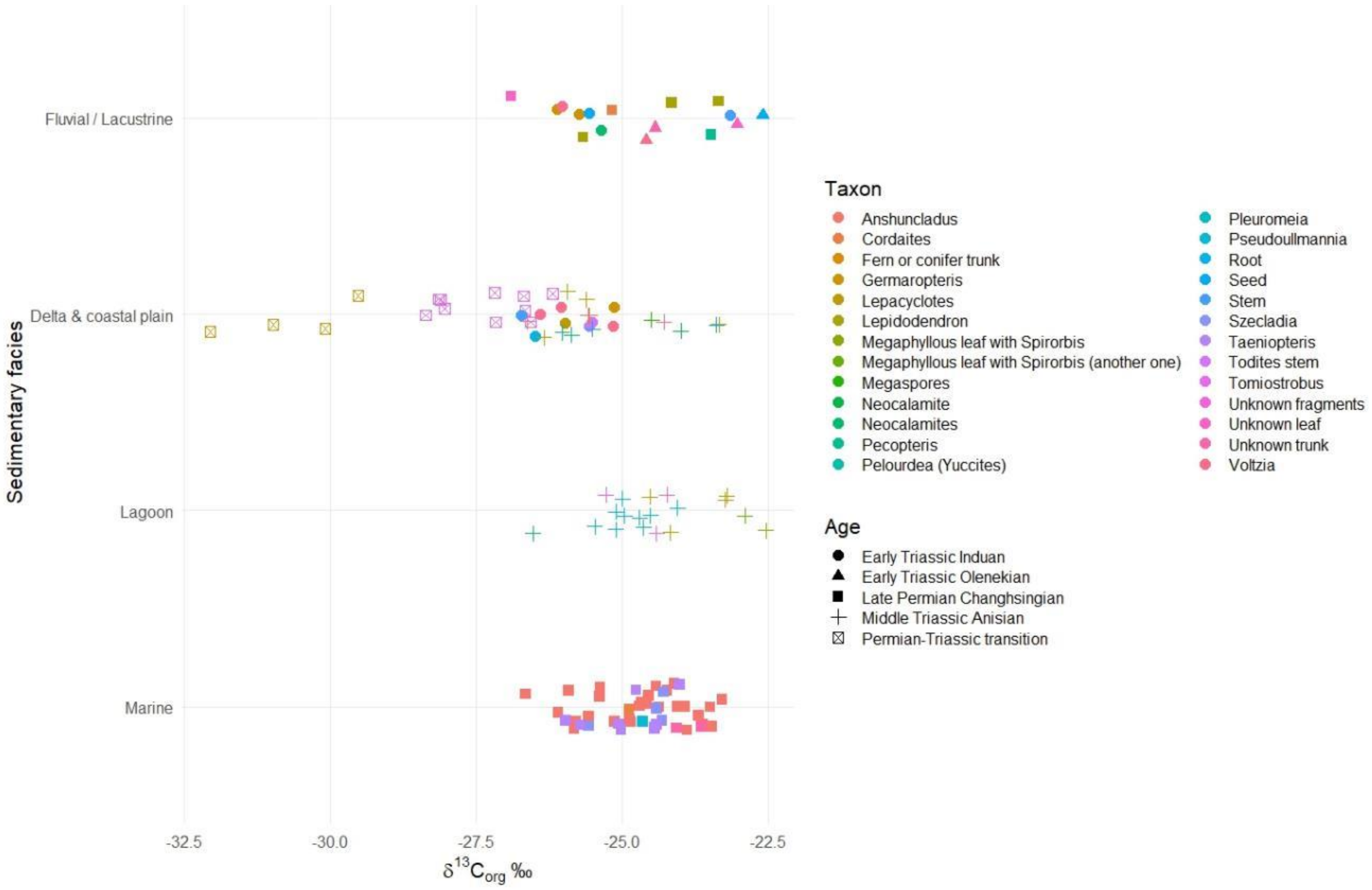
Sedimentary facies
Fluvial / Lacustrine
Delta & coastal plain
Lagoon
Marine
-32.5
-30.0
-27.5
-25.0
-22.5
$\delta^{13}C_{org}$ ‰
Taxon
Anshuncladus
Cordaites
Fern or conifer trunk
Germaropteris
Lepacyclotes
Lepidodendron
Megaphyllous leaf with Spirorbis
Megaphyllous leaf with Spirorbis (another one)
Megaspores
Neocalamite
Neocalamites
Pecopteris
Pelourdea (Yuccites)
Pleuromeia
Pseudoullmannia
Root
Seed
Stem
Szecladia
Taeniopteris
Todites stem
Tomiostrobus
Unknown fragments
Unknown leaf
Unknown trunk
Voltzia
Age
Early Triassic Induan
Early Triassic Olenekian
Late Permian Changhsingian
Middle Triassic Anisian
Permian-Triassic transition

Figure S8. $\delta^{13}C_{org}$ by sedimentary facies for late Permian to Middle Triassic lycophyte and non-lycophyte plants. Taxa are represented by colors, and age of each taxon is represented by shape. R code 3 for producing this figure is attached in this file. The result indicates sedimentary facies is not the primary control on $\delta^{13}C_{org}$; instead, age (reflecting background atmospheric CO_2) and genus exert stronger influence.

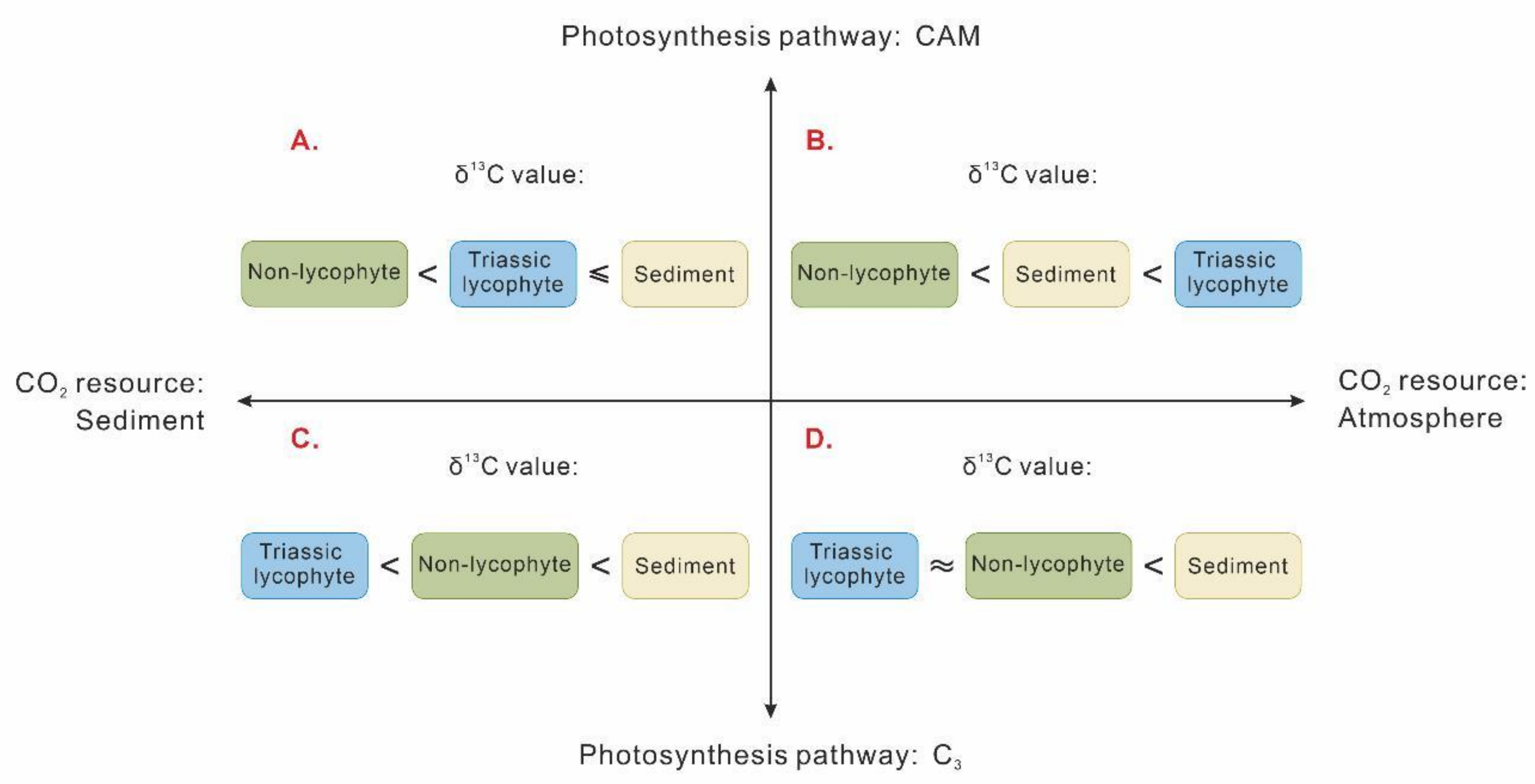

Fig. S9: Schematic diagram illustrating the $\delta^{13}C_{org}$ value ranges of sediments, Triassic lycophytes, and non-lycophyte plants, reflecting differences in photosynthetic pathways and CO_2 sources used by Triassic lycophytes. CAM photosynthesis typically produces more positive $\delta^{13}C_{org}$ values due to reduced discrimination against ^{13}C, whereas C_3 plants generally show more negative $\delta^{13}C_{org}$ values when using the same CO_2

source. CO_2 derived from decayed sedimentary organic matter is more depleted in ^{13}C compared to atmospheric CO_2, resulting in more negative $\delta^{13}C_{org}$ values. A. If Triassic lycophytes operated CAM photosynthesis using CO_2 from sedimentary organic matter, their $\delta^{13}C_{org}$ values would fall between those of non-lycophyte C_3 plants using atmospheric CO_2 and the $\delta^{13}C_{org}$ of the sediment-derived CO_2, depending on the proportion of CAM-fixed carbon. B. If Triassic lycophytes used CAM photosynthesis with atmospheric CO_2, their $\delta^{13}C_{org}$ values would be significantly more positive than both sediments and non-lycophyte plants. C. If Triassic lycophytes used C_3 photosynthesis with sediment-derived CO_2, their $\delta^{13}C_{org}$ values would be more negative than those of both sediments and non-lycophyte plants. D. If Triassic lycophytes used C_3 photosynthesis with atmospheric CO_2, their $\delta^{13}C_{org}$ values would be similar to those of non-lycophyte plants.

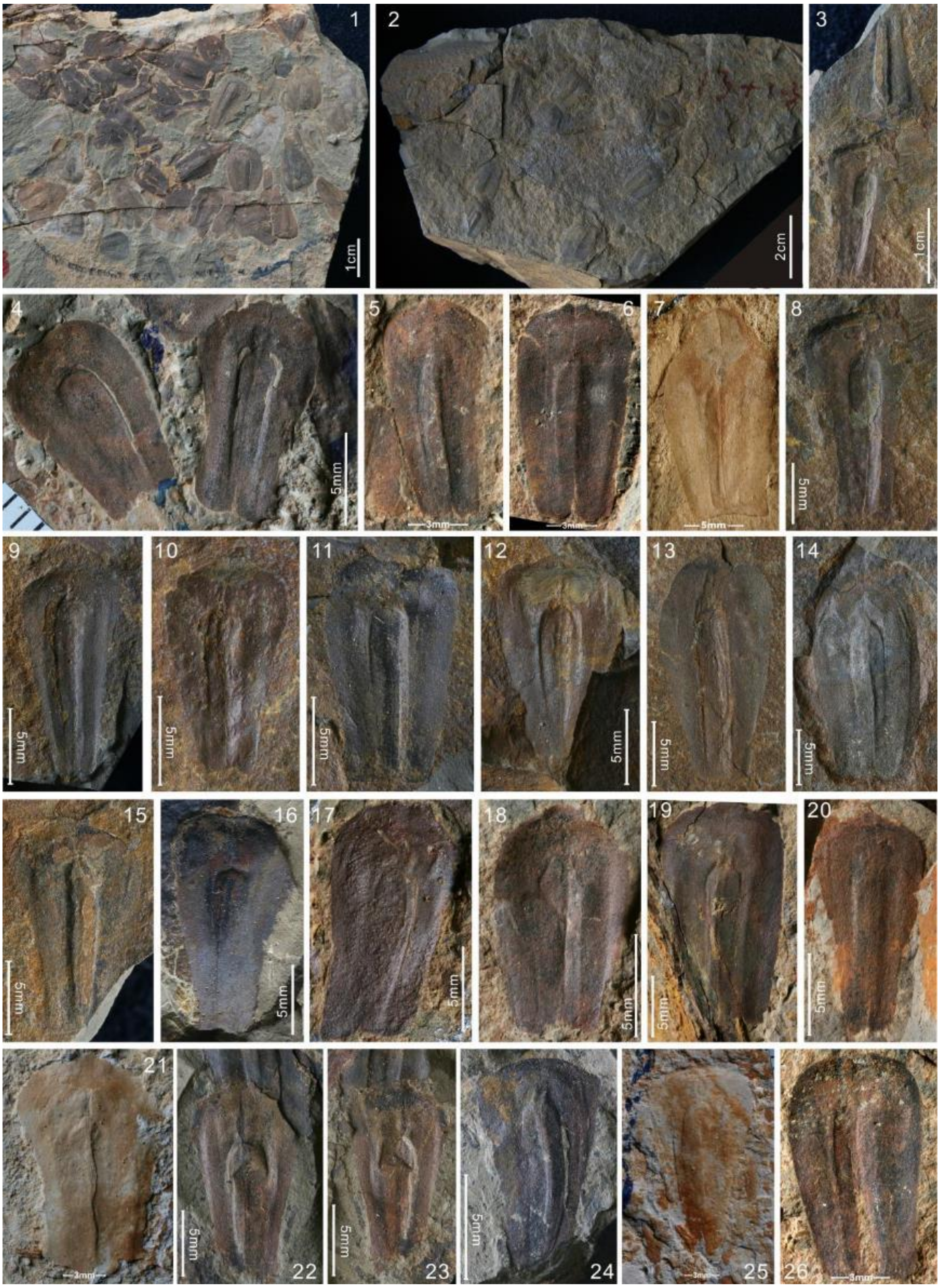

Fig. S10: Permian-Triassic transition *Tomiostrobus zeilleri* sporophylls in South China. Locality: 1, 4, 5–7, 17–19, 26 from Mide section, Xuanwei City, Yunnan Province; 2, 3, 8–16, 22–24 from Chinahe section, Xuanwei City, Yunnan Province; 20 from Jinzhong section, Weining County, Guizhou Province; 21, 25 from Tucheng section, Panxian County, Guizhou Province. Picture 22 is modified from Yu et al.[6] and all the others are taken by Jianxin Yu and Zhen Xu. Permission to reuse the relevant panel from Yu et al.[6] has been obtained from Springer Nature via RightsLink (License

No. 6194800725238).

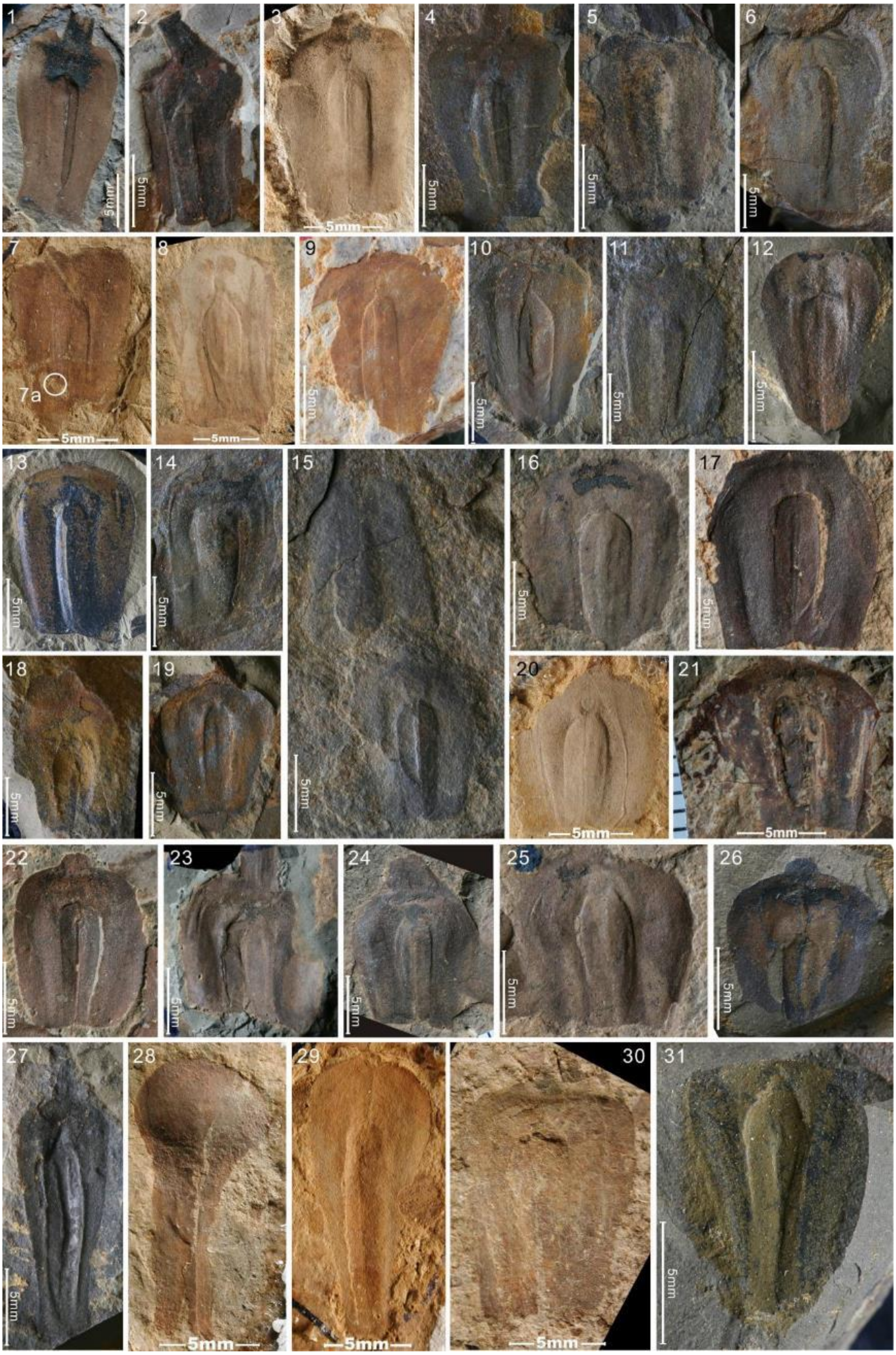

Fig. S11: Permian-Triassic transition *Tomiostrobus zeilleri*, *T.* sp.1 and *T.* sp.2 sporophylls in South China. Locality: 1, 3, 7, 8, 15, 16, 20–25, 28–30 from Mide

section, Xuanwei City, Yunnan Province; 2 from Jinzhong section, Weining County, Guizhou Province; 9 from Tucheng section, Panxian County, Guizhou Province; 4-6, 10-14, 17-19, 26, 27, 31 from Chinahe section, Xuanwei City, Yunnan Province. Picture 13, 19, 27 are modified from Yu et al.[6] and all the others are taken by Jianxin Yu and Zhen Xu. Permission to reuse the relevant panel from Yu et al.[6] has been obtained from Springer Nature via RightsLink (License No. 6194800725238).

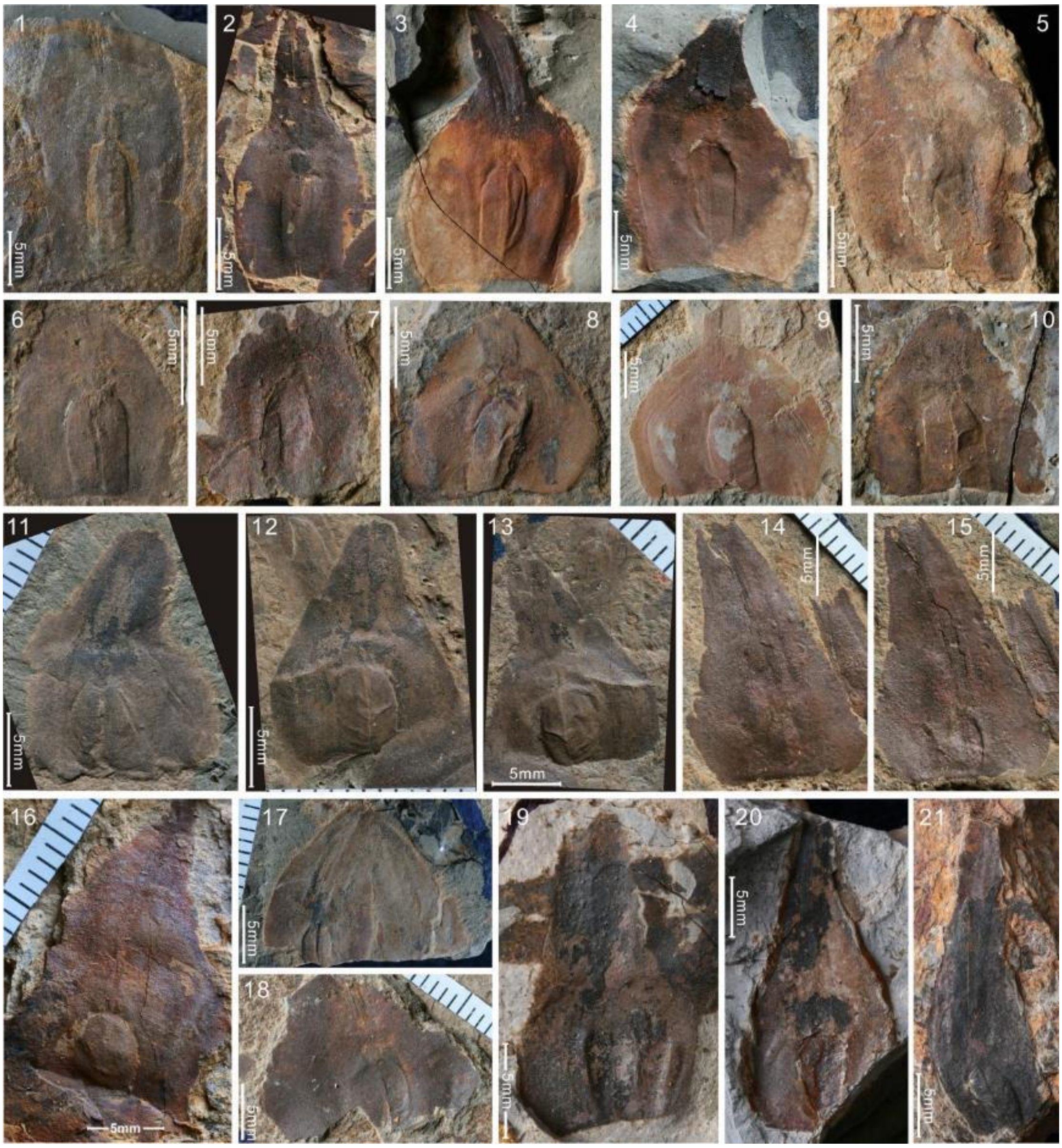

Fig. S12: Permian-Triassic transition *Tomiostrobus brevicystis* sporophylls in South China. Locality: 2–18 from Mide section, Xuanwei City, Yunnan Province; 19–21 from Jinzhong section, Weining County, Guizhou Province; 1 from Chinahe section, Xuanwei City, Yunnan Province. Pictures by Jianxin Yu and Zhen Xu.

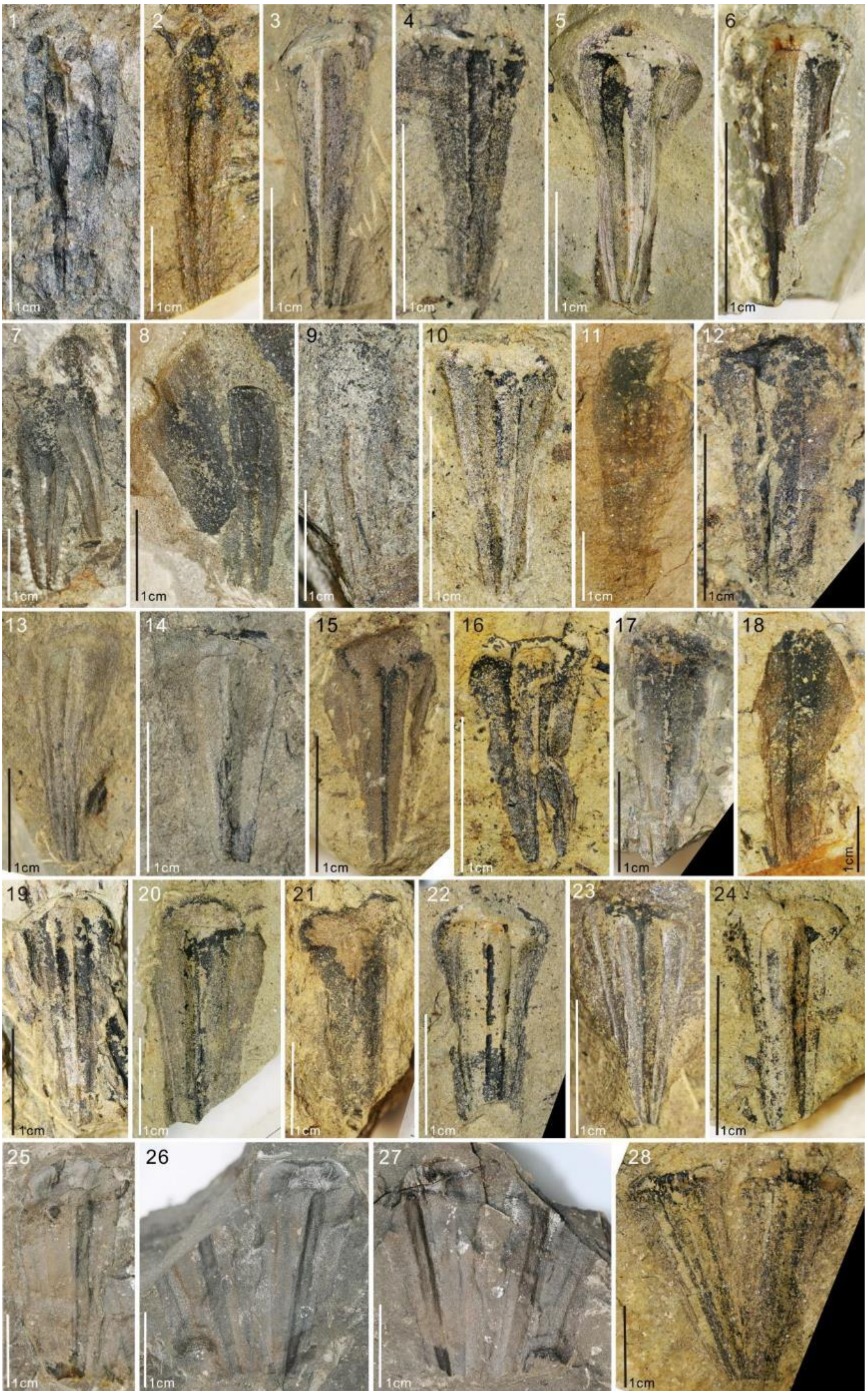

Fig. S13: Middle Triassic *Lepacyclotes zeilleri* sporophylls in South China. 1 has a

ligular pit in the upper part of the sporophyll. Locality: 1, 25–27 from Section HSF2, Furongqiao Village, Hunan Province; 2, 11, 18 from Section HSF1, Furongqiao Village, Hunan Province; 3-6, 10, 12–17, 19–24, 28 from Section HSF4, Furongqiao Village, Hunan Province; 7-9 from Section HSF3, Furongqiao Village, Hunan Province. All fossils deposited in Main Building of China University of Geosciences (Wuhan), Hubei Province, P. R. China. Pictures by Zhen Xu.

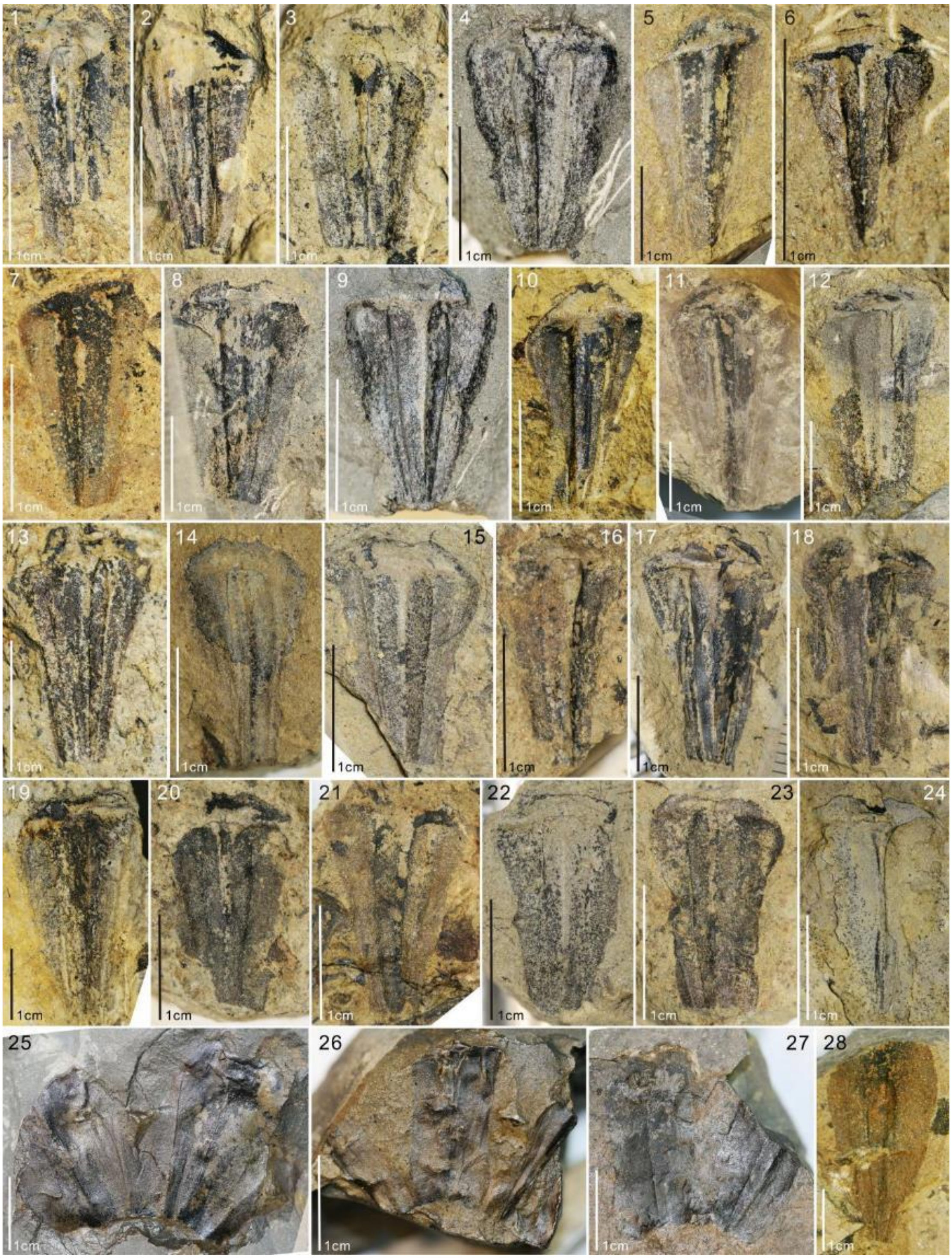

Fig. S14: Middle Triassic *Lepacyclotes zeilleri* sporophylls in South China. Locality:

1–24 from Section HSF4, Furongqiao Village, Hunan Province; 25–27 from Section HSF2, Furongqiao Village, Hunan Province; 28 from Section HSF1, Furongqiao Village, Hunan Province. All fossils are stored in Main Building of China University of Geosciences (Wuhan), Hubei Province, P. R. China. Pictures by Zhen Xu.

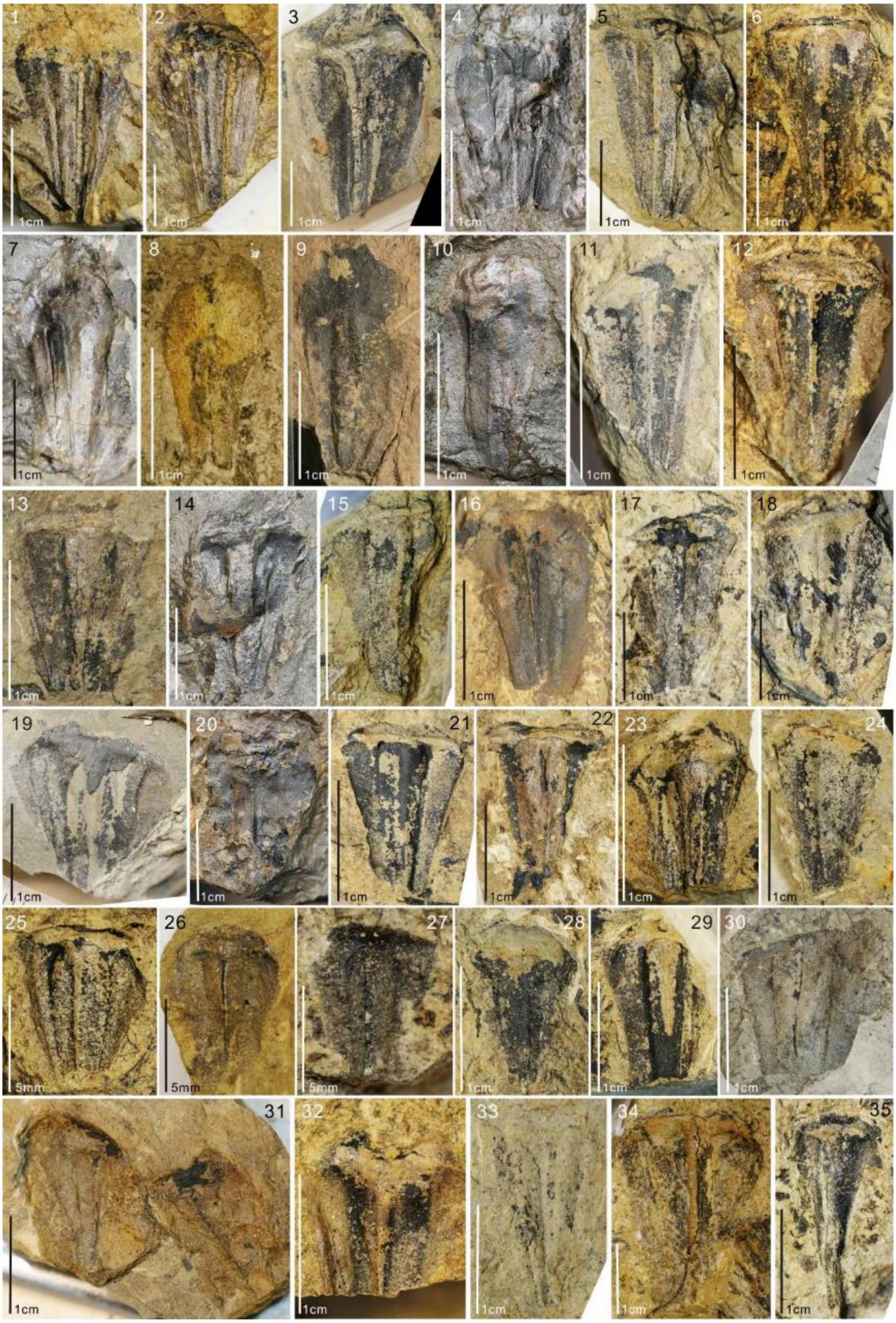

Fig. S15: Middle Triassic *Lepacyclotes zeilleri* sporophylls in South China. Locality: 1–3, 5, 6, 9, 11–13, 15–19, 21–29, 32–35 from Section HSF4, Furongqiao Village, Hunan Province; 4, 7, 10, 14, 20 from Section HSF2, Furongqiao Village, Hunan Province; 8 from Section HSF1, Furongqiao Village, Hunan Province; 30 from Section

HSF3, Furongqiao Village, Hunan Province; 31 from Section HSH1, Hongjiaguan Village, Hunan Province. All fossils are stored in Main Building of China University of Geosciences (Wuhan), Hubei Province, P. R. China. Pictures by Zhen Xu.

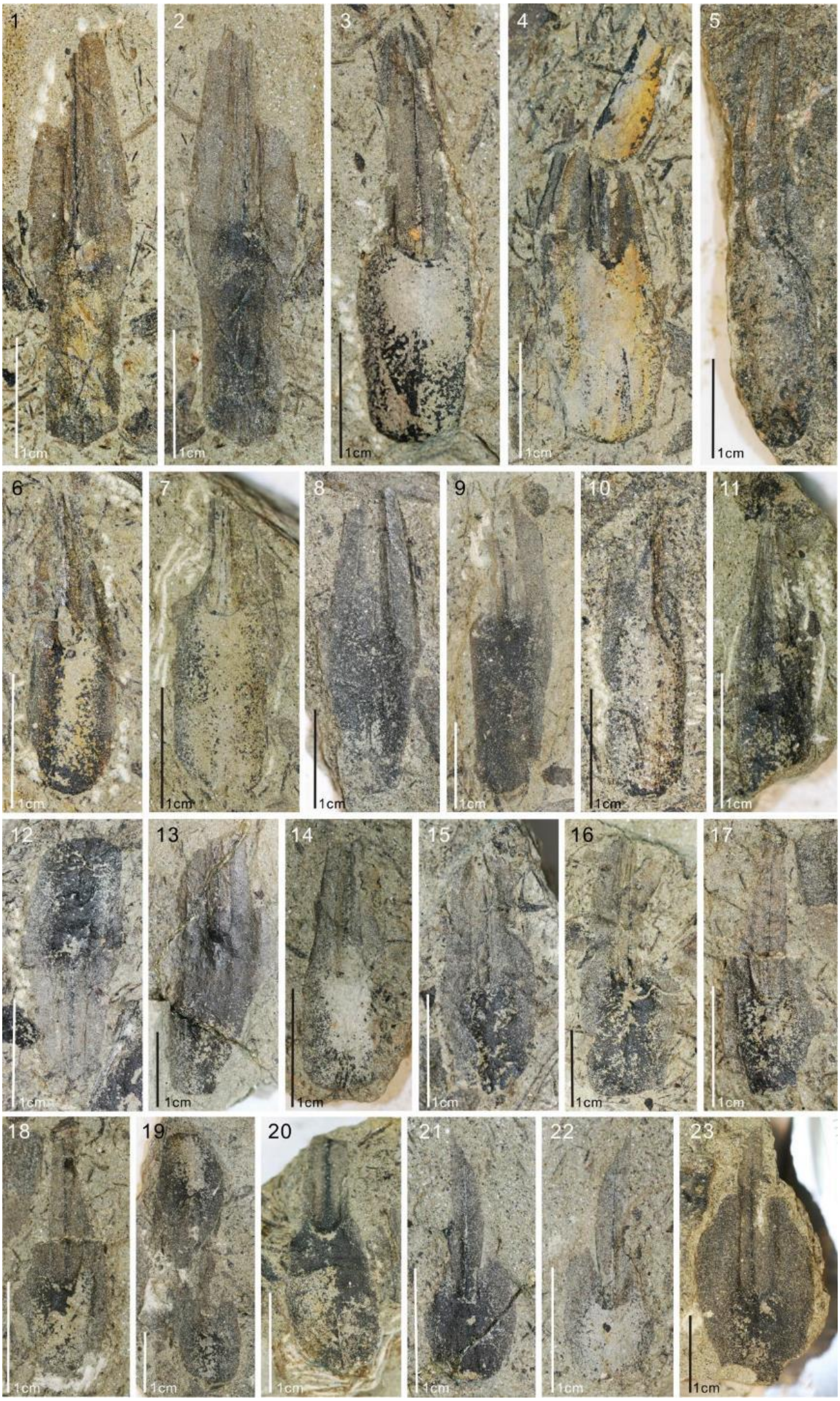
1
2
3
4
5
6
7
8
9
10
11
12
13
14
15
16
17
18
19
20
21
22
23
1cm

Fig. S16: Middle Triassic *Lepacyclotes* sp. 2 with long sporophyll apex in South China. 1 has a ligular pit in the middle of sporophyll. Locality: 1–23 from Section HSF3, Furongqiao Village, Hunan Province. All fossils are stored in Main Building of China University of Geosciences (Wuhan), Hubei Province, P. R. China. Pictures by Zhen Xu.

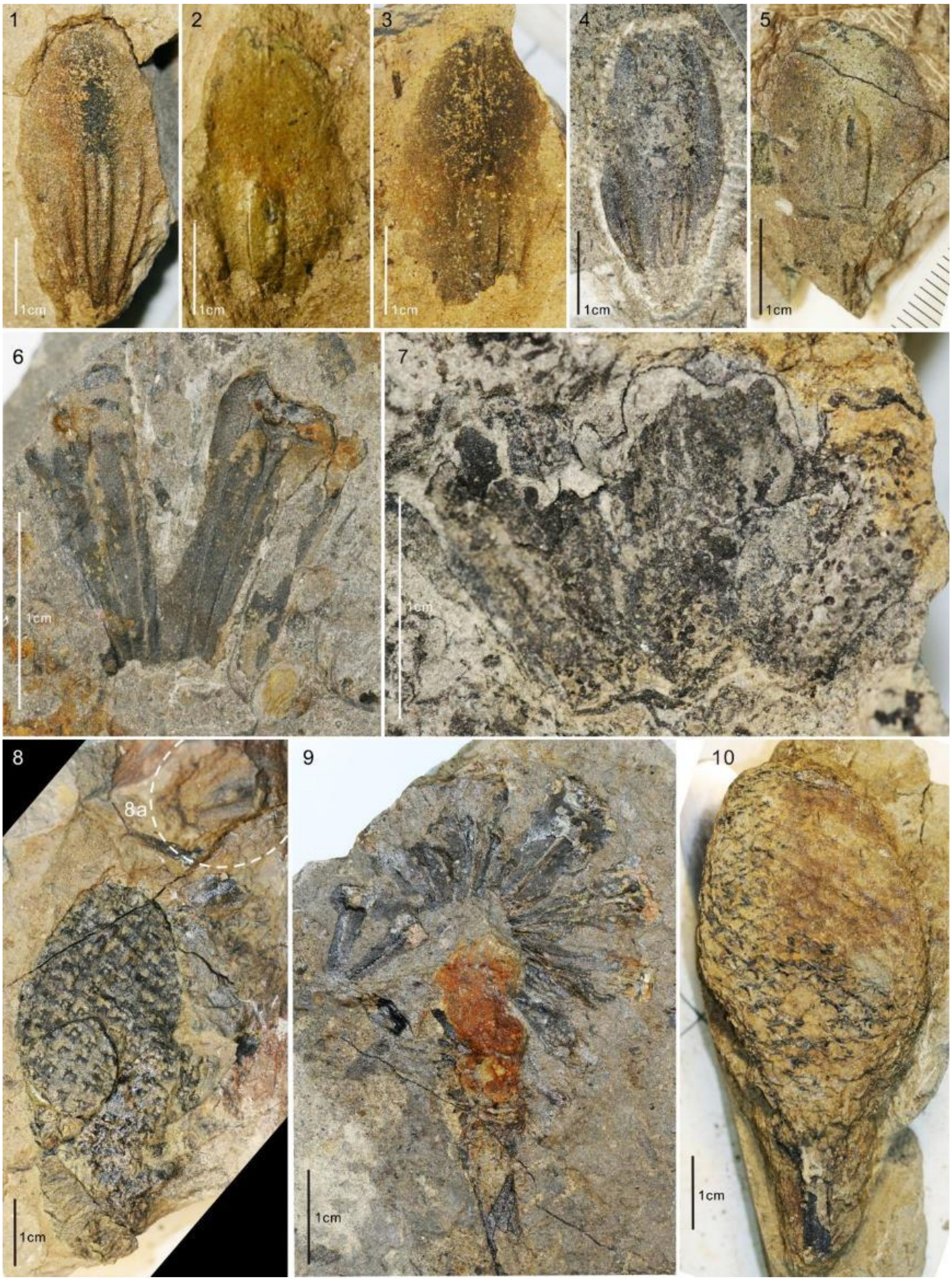

Fig. S17: *Lepacyclotes* sporophylls and strobili in the Middle Triassic Badong Formation, South China. 1–5: *Lepacyclotes brevicystis* sporophylls; 6: *Lepacyclotes*

zeilleri sporophyll preserved with aquatic animal Conchostraca; 7: *Lepacyclotes zeilleri* sporophyll with in-situ spores; 8–10: Cones of *Lepacyclotes* preserved in same beds as disperse sporophylls. Locality: 1–3, 8 from Section HSF1, Furongqiao Village, Hunan Province; 4 from Section HSF3, Furongqiao Village, Hunan Province; 5, 7, 10 from Section HSF4, Furongqiao Village, Hunan Province; 6, 9 from Section HSF2, Furongqiao Village, Hunan Province. All fossils are stored in Main Building of China University of Geosciences (Wuhan), Hubei Province, P. R. China. Pictures by Zhen Xu.

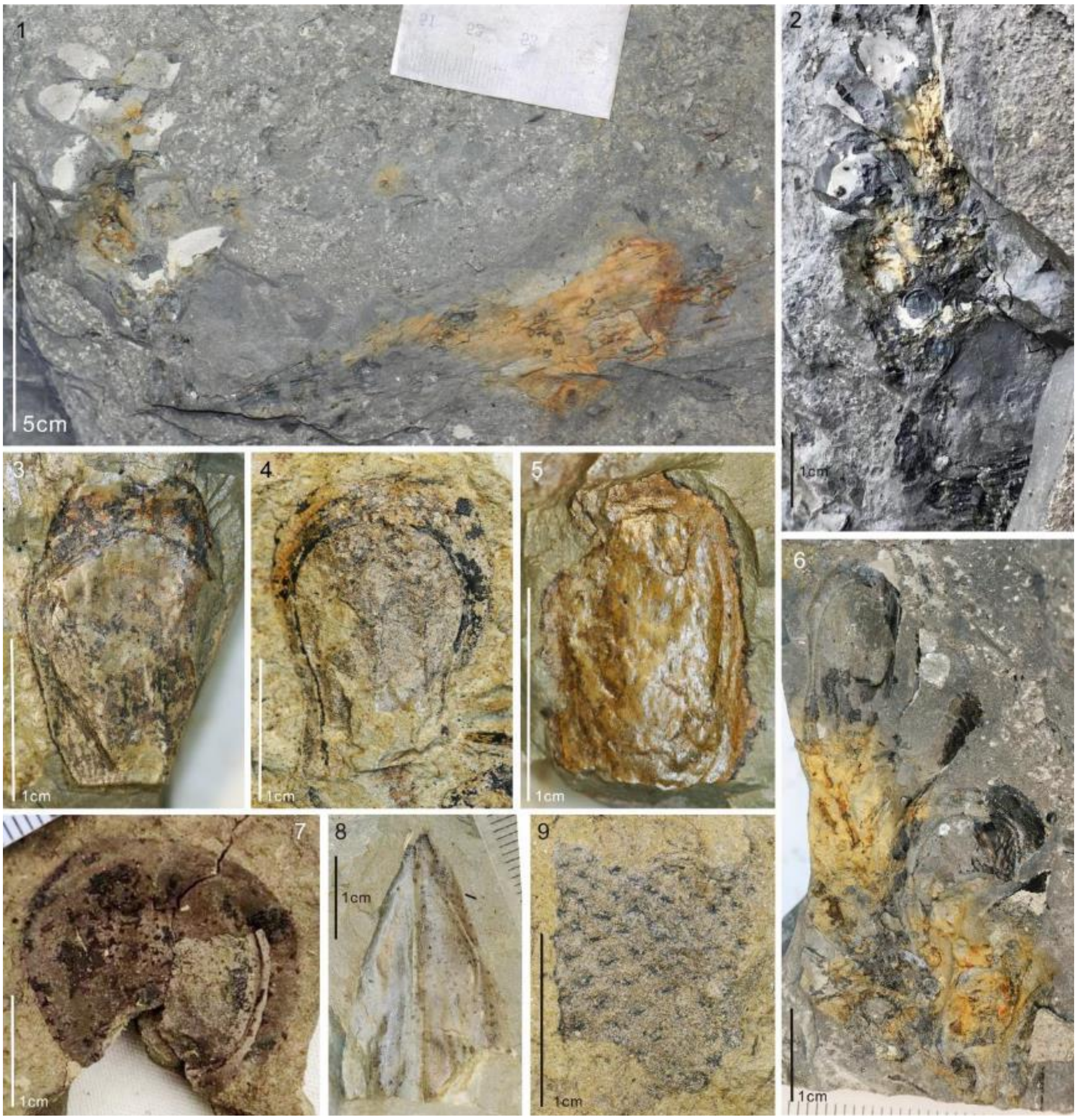

Fig. S18: Middle Triassic *Pleuromeia* whole plant and dispersed sporophylls in South China. 1, 2, 6: whole plant of *Pleuromeia hunanensis*; 3–5: dispersed sporophylls of *Pleuromeia hunanensis*; 7: dispersed sporophyll of *Pleuromeia marginulata*; 8: vegetative leaf of *Pleuromeia*; 9: trunk with leaf scar of *Lepacyclotes* or *Pleuromeia*. Locality: 1, 2, 6 from Section HSF2, Furongqiao Village, Hunan Province; 3–5, 8 from Section HSH3, Hongjiaguan Village, Hunan Province; 7, 9 from Section HSH2, Hongjiaguan Village, Hunan Province. All fossils are stored in Main Building of China University of Geosciences (Wuhan), Hubei Province, P. R. China.

Pictures by Zhen Xu.

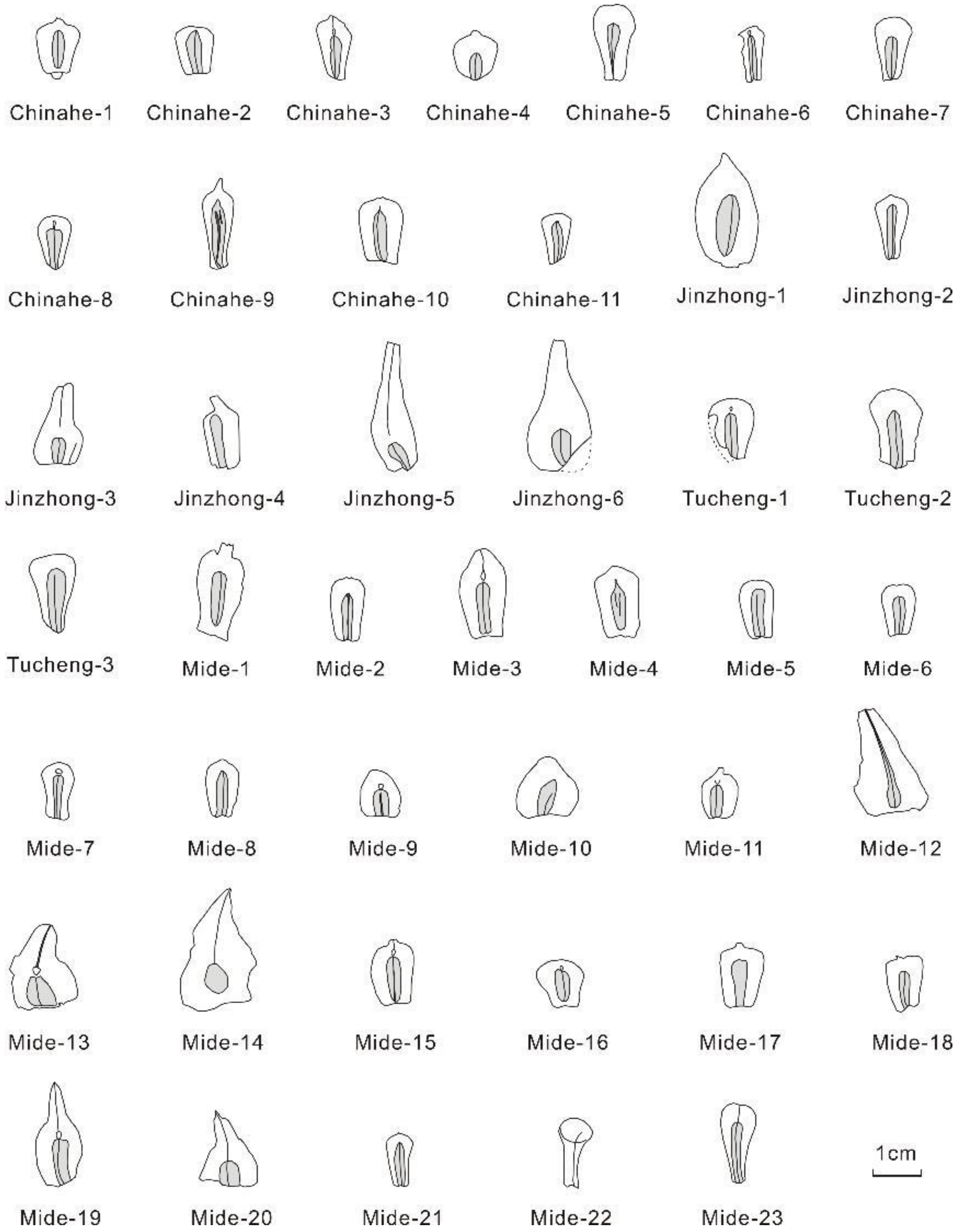

Fig. S19: Sketches of Permian-Triassic transition *Tomiostrobus* sporophylls in Figure S6 to S8. Sketches by Zhen Xu.

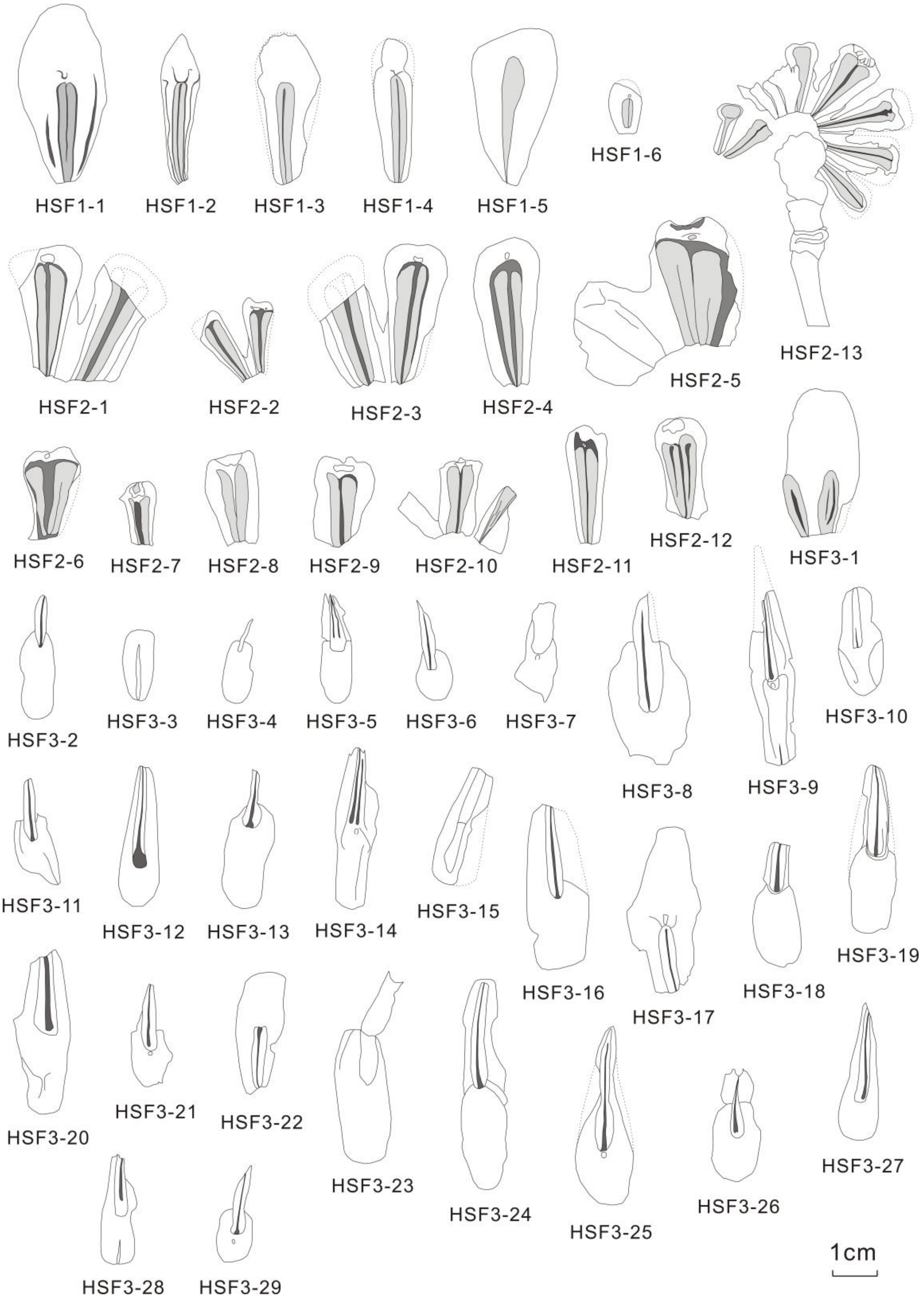

Fig. S20: Sketches of Middle Triassic *Lepacyclotes* sporophylls in figure S9 to S13.
Sketches by Zhen Xu.

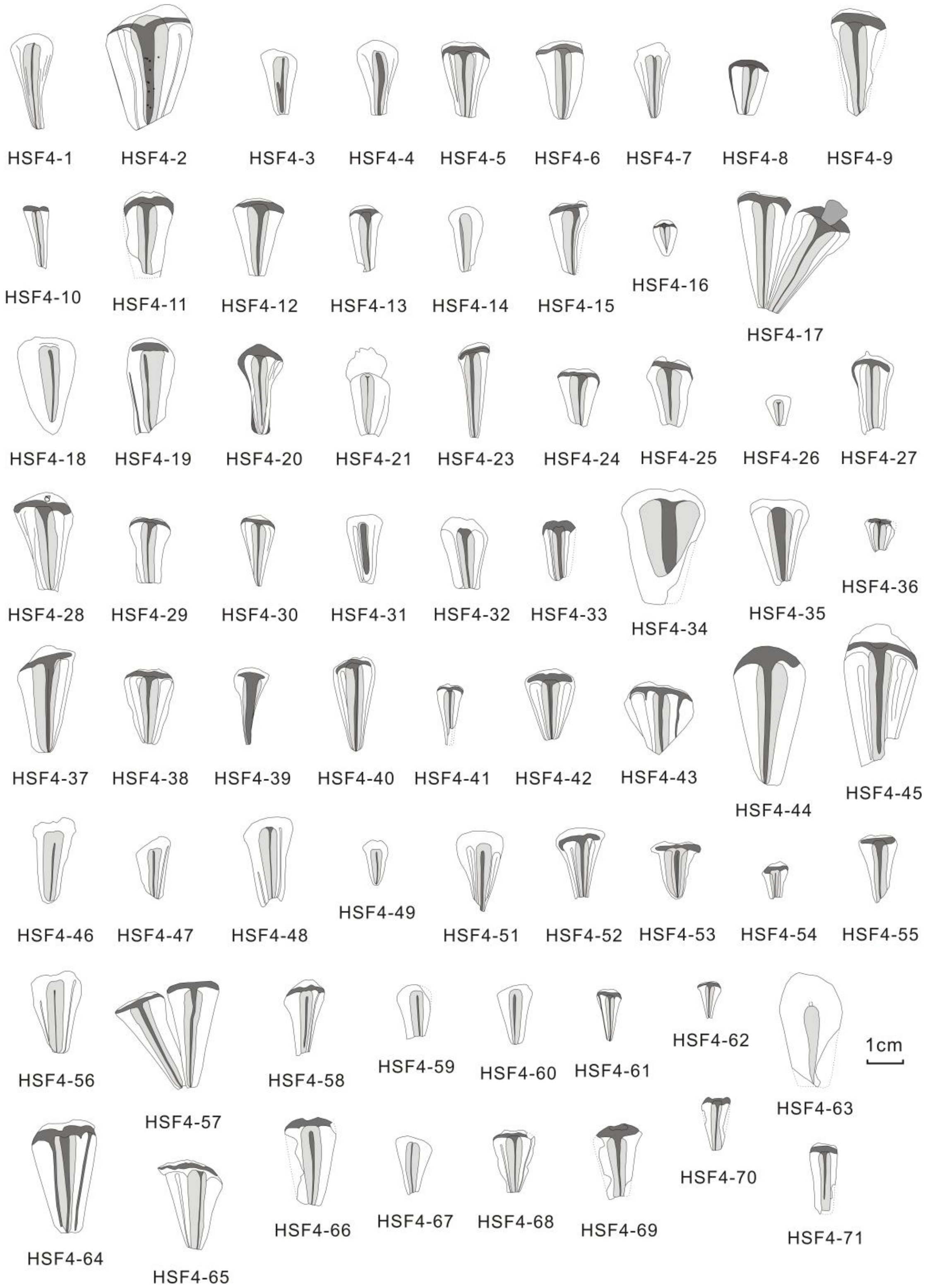

Fig. S21: Sketches of Middle Triassic *Lepacyclotes* sporophylls in figure S9 to S13. Sketches by Zhen Xu.

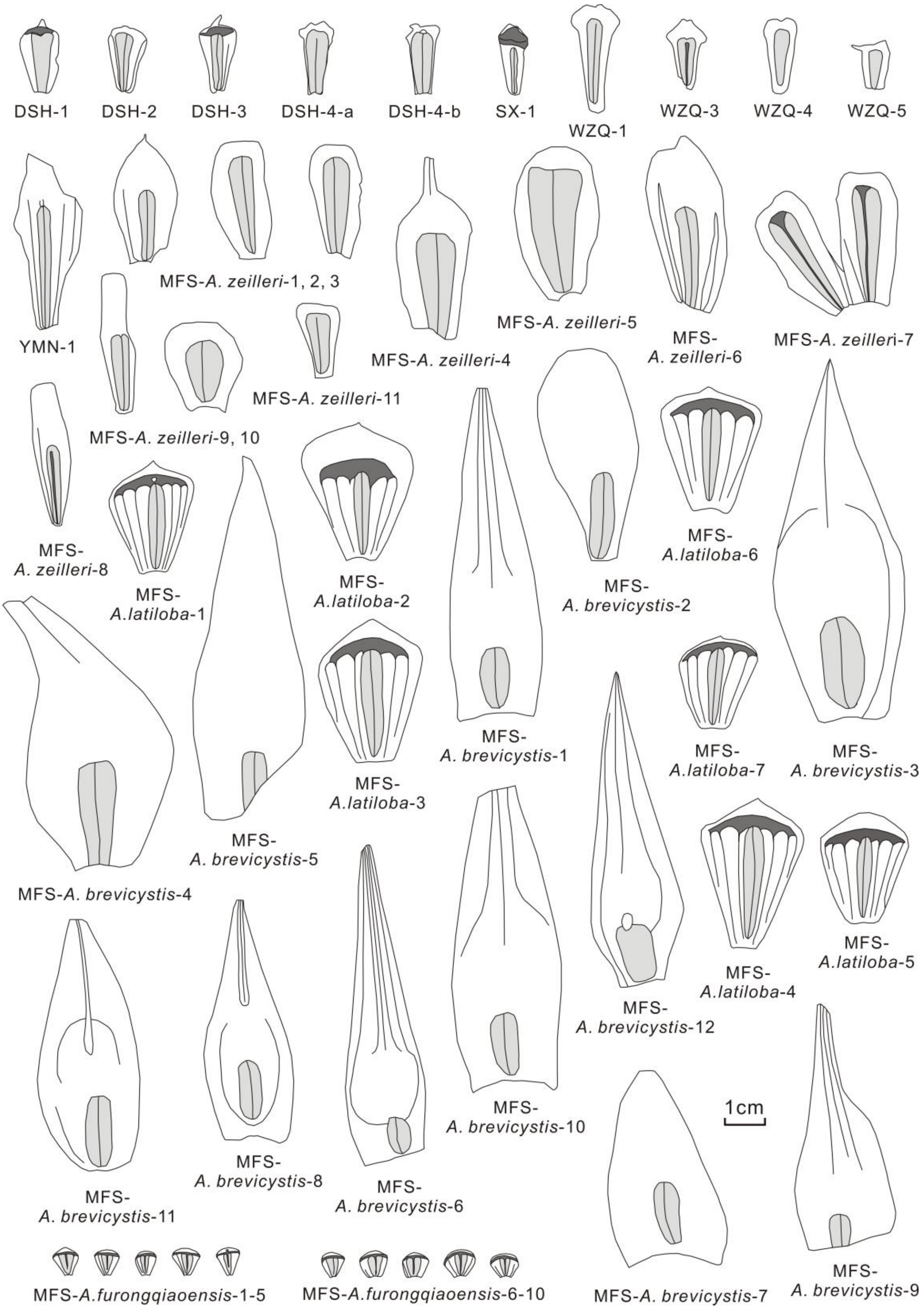

Fig. S22: Sketches of Middle Triassic *Lepacyclotes* sporophylls in China from references. Samples named as DSH come from Deng et al.[28], WZQ come from Wang and Lou[80], YMN come from Ye[81], MFS come from Fansong Meng's collections. Sketches by Zhen Xu.

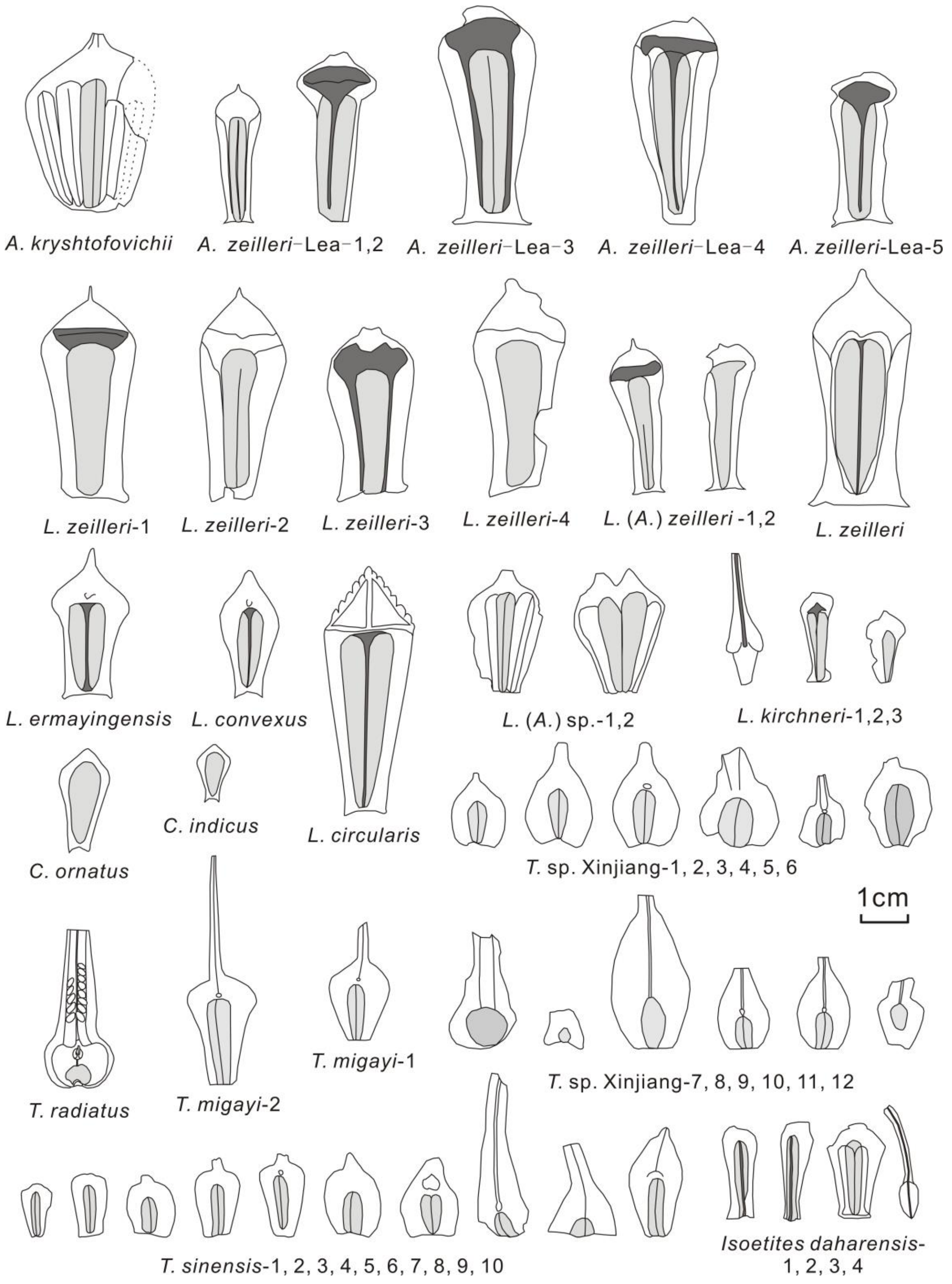

Fig. S23: Sketches of global lycopod sporophylls from references. All the samples come from the references listed in Table 2. Sketches by Zhen Xu.

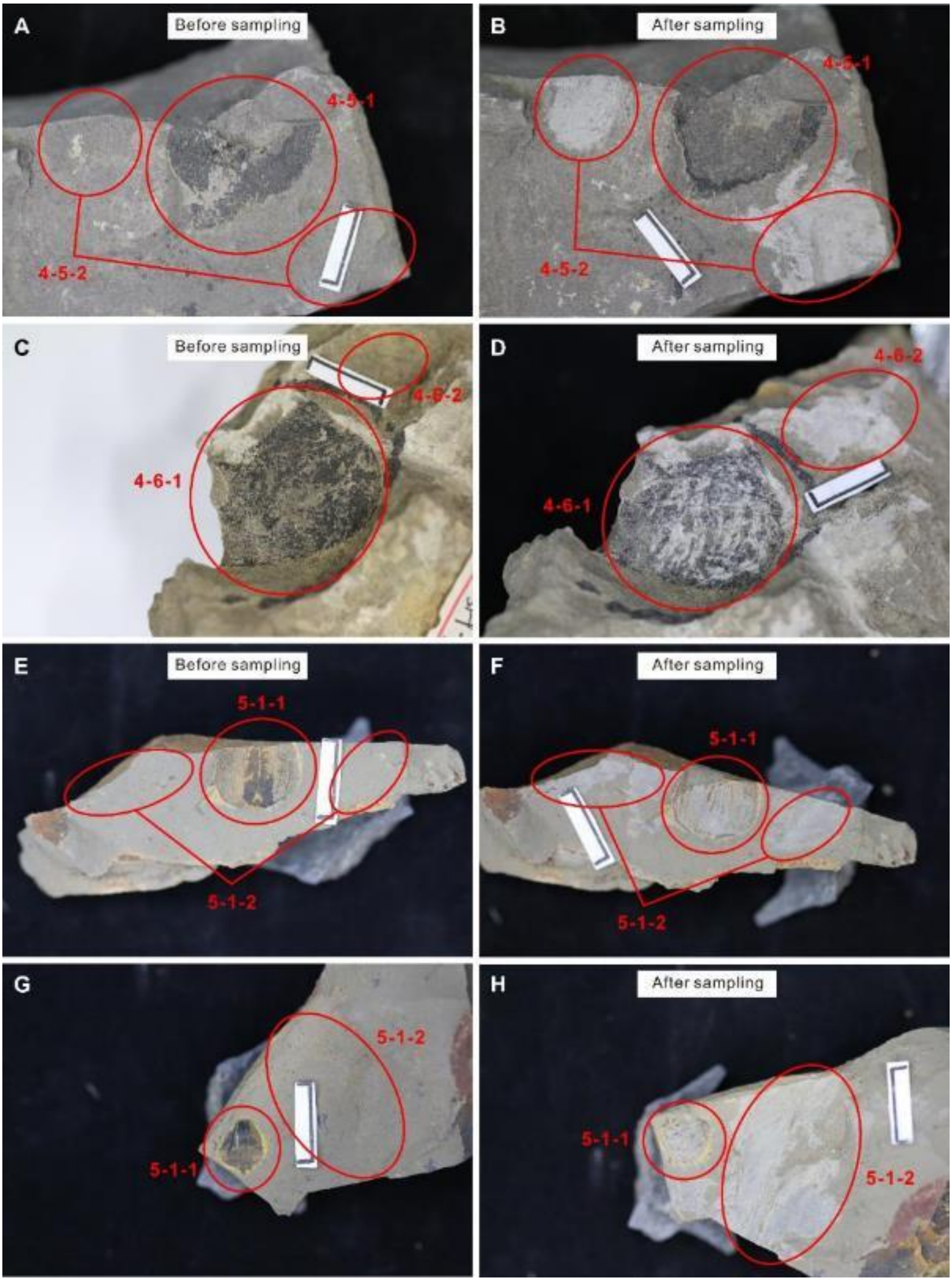

Fig. S24: Carbon isotope sampling locations on the fossils. All the plant fragment samples have names with 1 at the end, and the surrounding rock samples have number have 2 at the end. A, C, E, G are pictures taken before sampling, and B, D, F, H are taken after sampling. A. 4-5-1 is putative *Lepacyclotes* sporophyll; C. 4-6-1 is a megaphyllous leaf with *Spirobis*; E, G. 5-1-1 is *Tomiostrobus* sporophyll. Due to the

small amount of carbon tissues in the plant body of *Tomiostrobus*, sample 5-1-1 is taken from two *Tomiostrobus* fossils from two rocks in the sedimentaty bed. All scale bars are 1cm. Pictures by Zhen Xu.

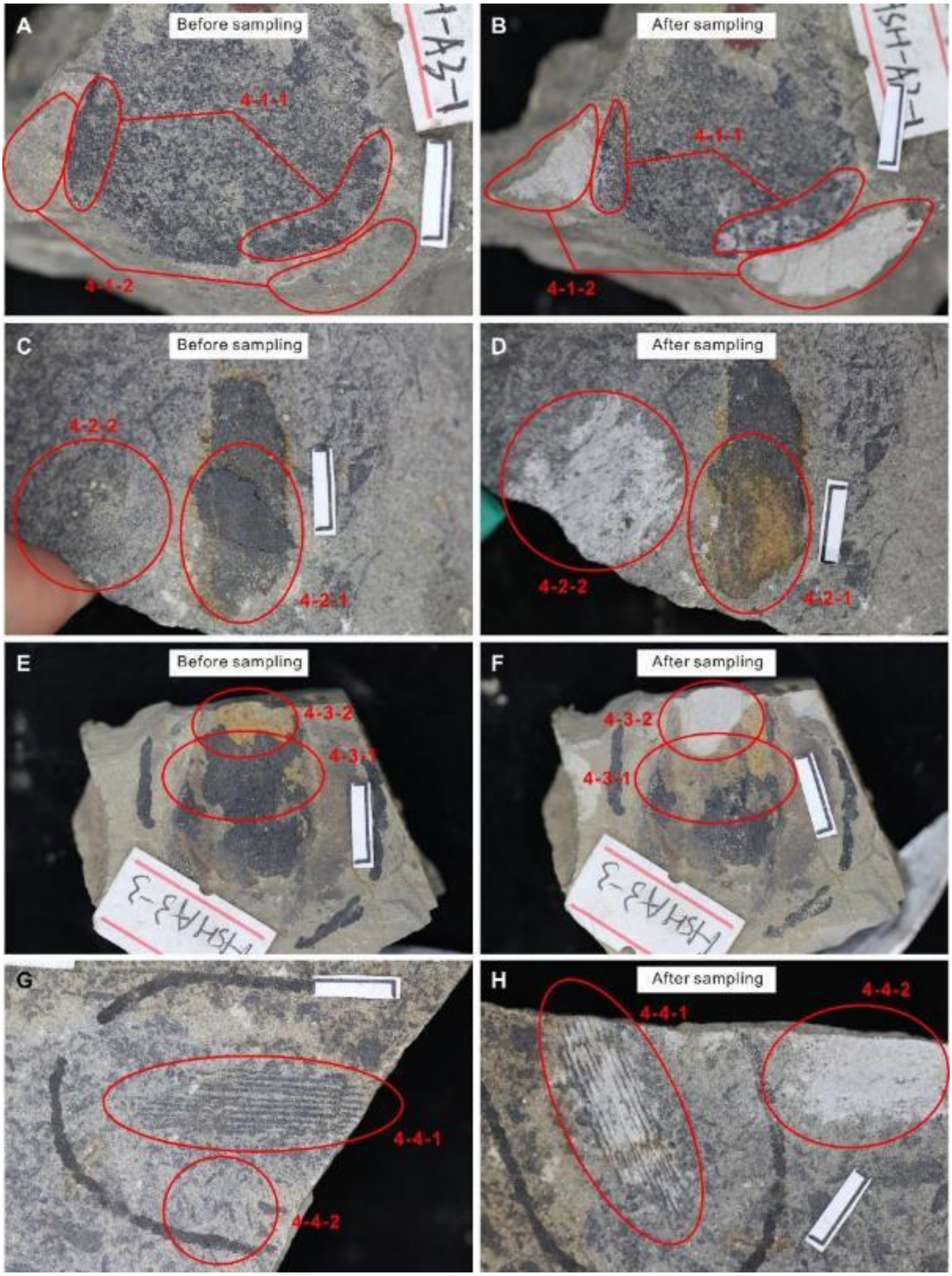

Fig. S25: Carbon isotope sampling locations on the fossils. All the plant fragment

samples names have number 1 at the end, and the surrounding rock sample names have number have number 2 at the end. A, C, E, G are pictures taken before sampling, and B, D, F, H are taken after sampling. A. 4-1-1 is a megaphyllous leaf with *Spirobis* on the surface; C. 4-2-1 is a possible lycopod trunk; E. 4-3-1 is a possible fragmented *Lepacyclotes* sporophyll; G. 4-4-1 is *Neocalamites* trunk. All the scale bars are 1cm. Pictures by Zhen Xu.

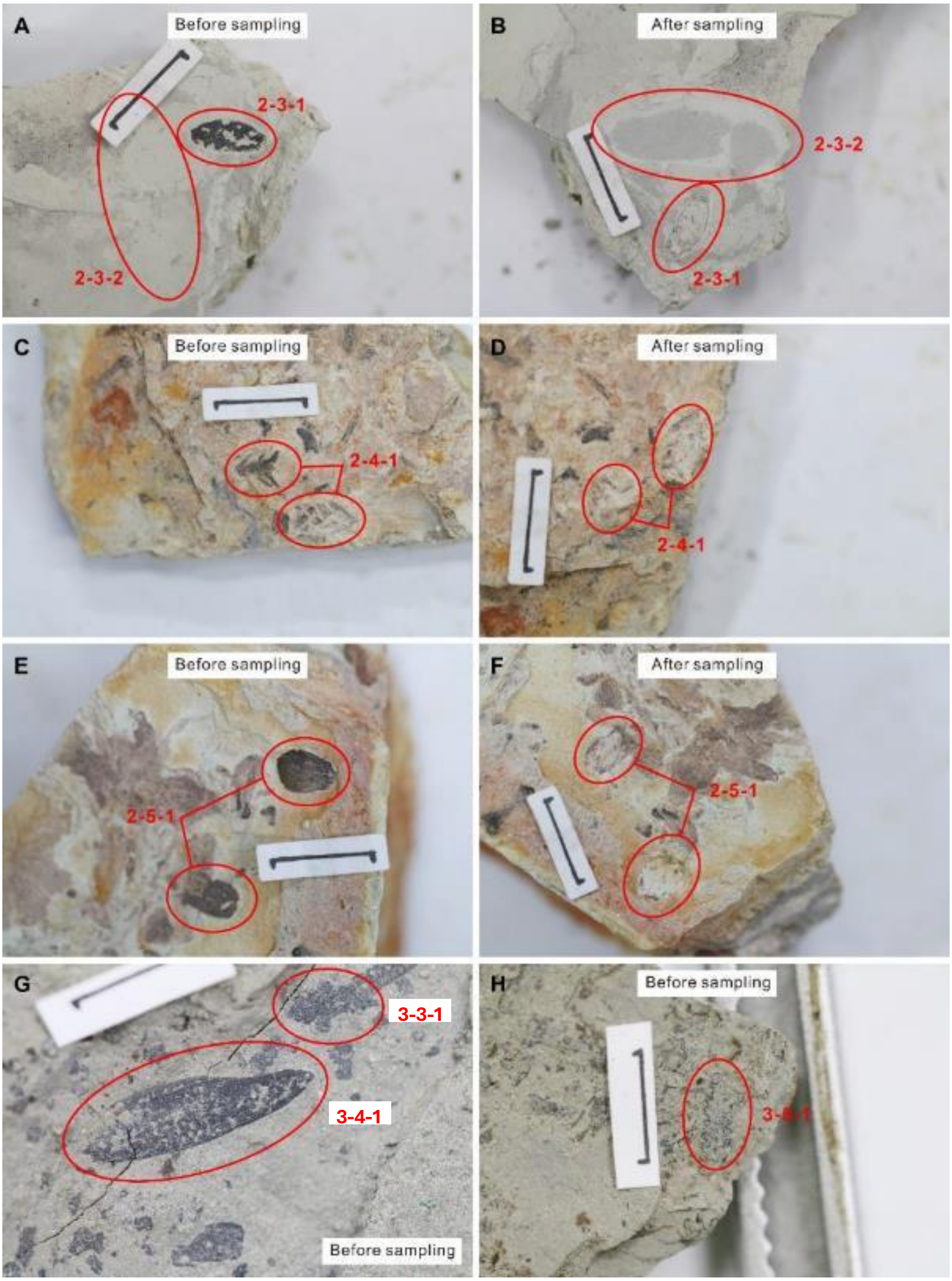

Fig. S26: Carbon isotope sampling locations on the fossils. All the plant fragment samples have names ending in 1, and the surrounding rock samples have names ending in number 2. A, C, E, G, H are pictures taken before sampling, and B, D, F are taken after sampling. A. 2-3-1 is a seed and is mixed with 2-2-1 due to the small sample amount; C. 2-4-1 is a conifer branch; E. 2-5-1 is unknown leaf with middle vein and

vague parallel veins; G. 3-3-1 is a *Germaropteris* leaf (*Lepidopteris*) and 3-4-1 is an unknown leaf; H. 3-5-1 is *Germaropteris* (*Lepidopteris*) and is mixed with 3-3-1 due to insufficient sampling amount. All scale bars are 1cm. Pictures by Zhen Xu.

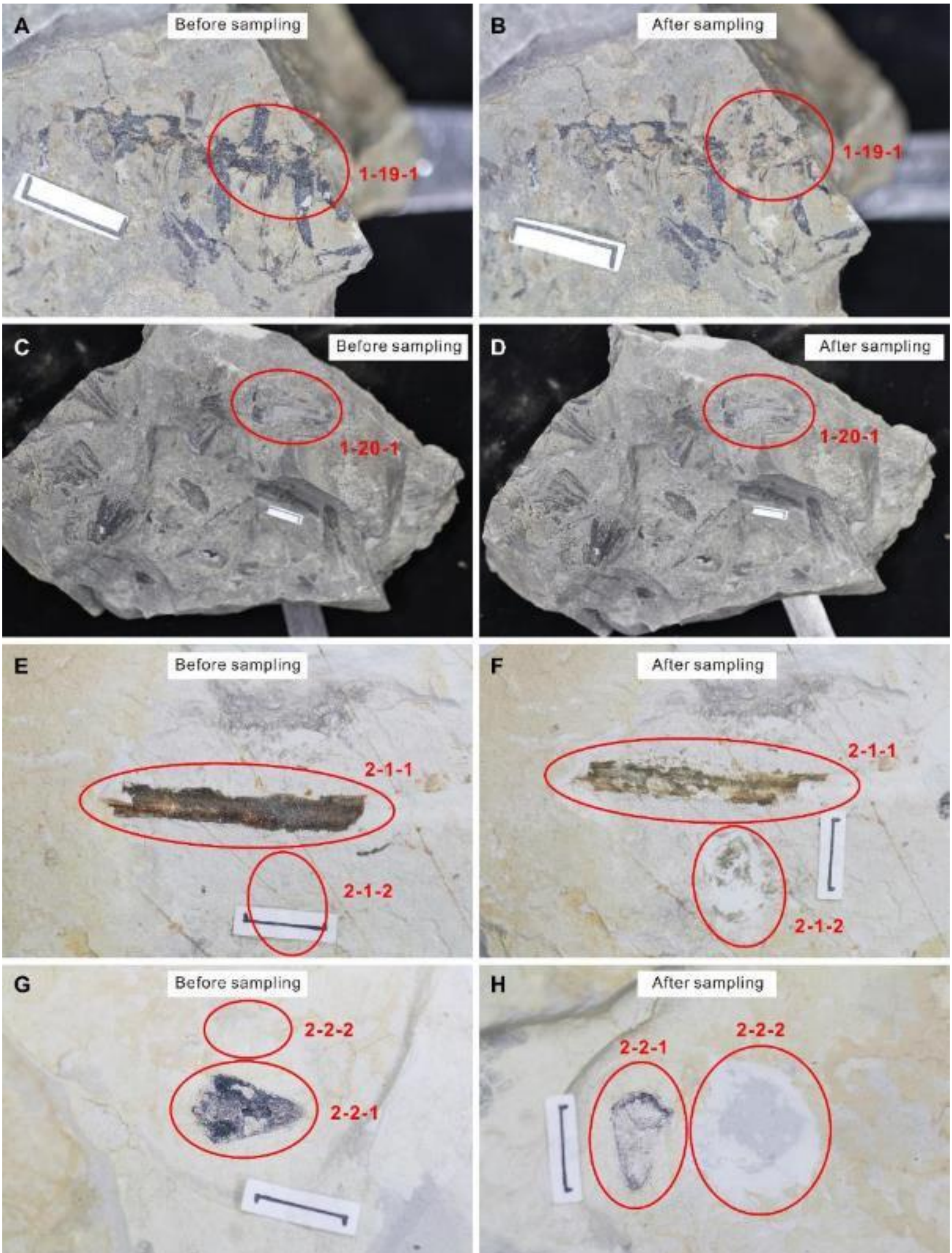

Fig. S27: Carbon isotope sampling locations on the fossils. All the plant fragment

samples names end with 1, and the surrounding rock samples names end with 2. A, C, E, G are pictures taken before sampling, and B, D, F, H are taken after sampling. A. 1-19-1 is a *Voltzia* branch with leaves; C. 1-20-1 is *Lepacyclotes* sporophyll; E. 2-1-1 is an unknown trunk; G. 2-2-1 is a seed. All scale bars are 1cm. Pictures by Zhen Xu.

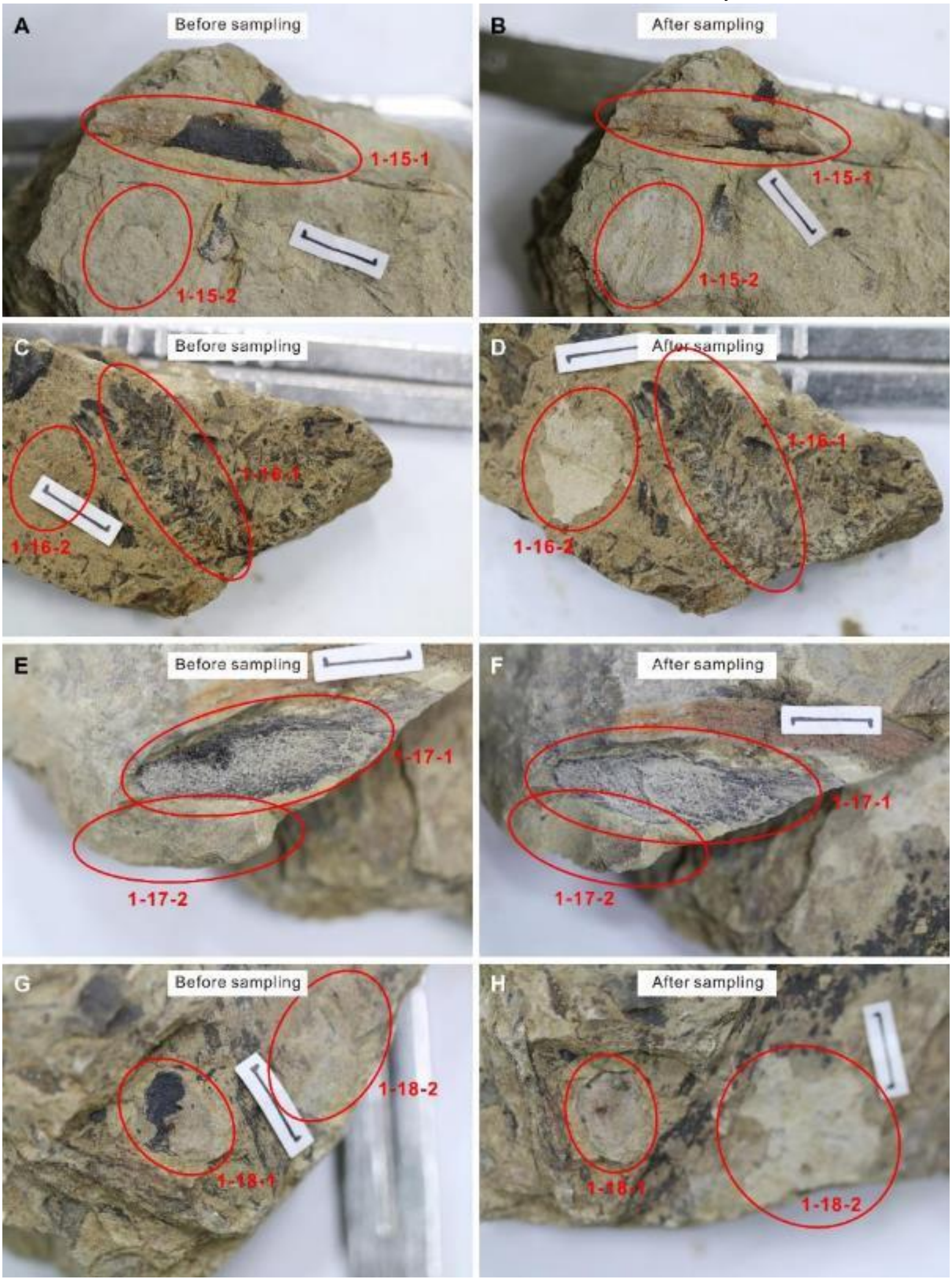

Fig. S28: Carbon isotope sampling locations on the fossils. All the plant fragment

samples names end with 1, and the surrounding rock samples named end with number 2. A, C, E, G are before sampling, and B, D, F, H are taken after sampling. A. 1-15-1 is a fern or conifer trunk; C. 1-16-1 is a *Voltzia* trunk and leaves; E. 1-17-1 is a conifer trunk; G. 1-18-1 is a seed. All scale bars are 1cm. Pictures by Zhen Xu.

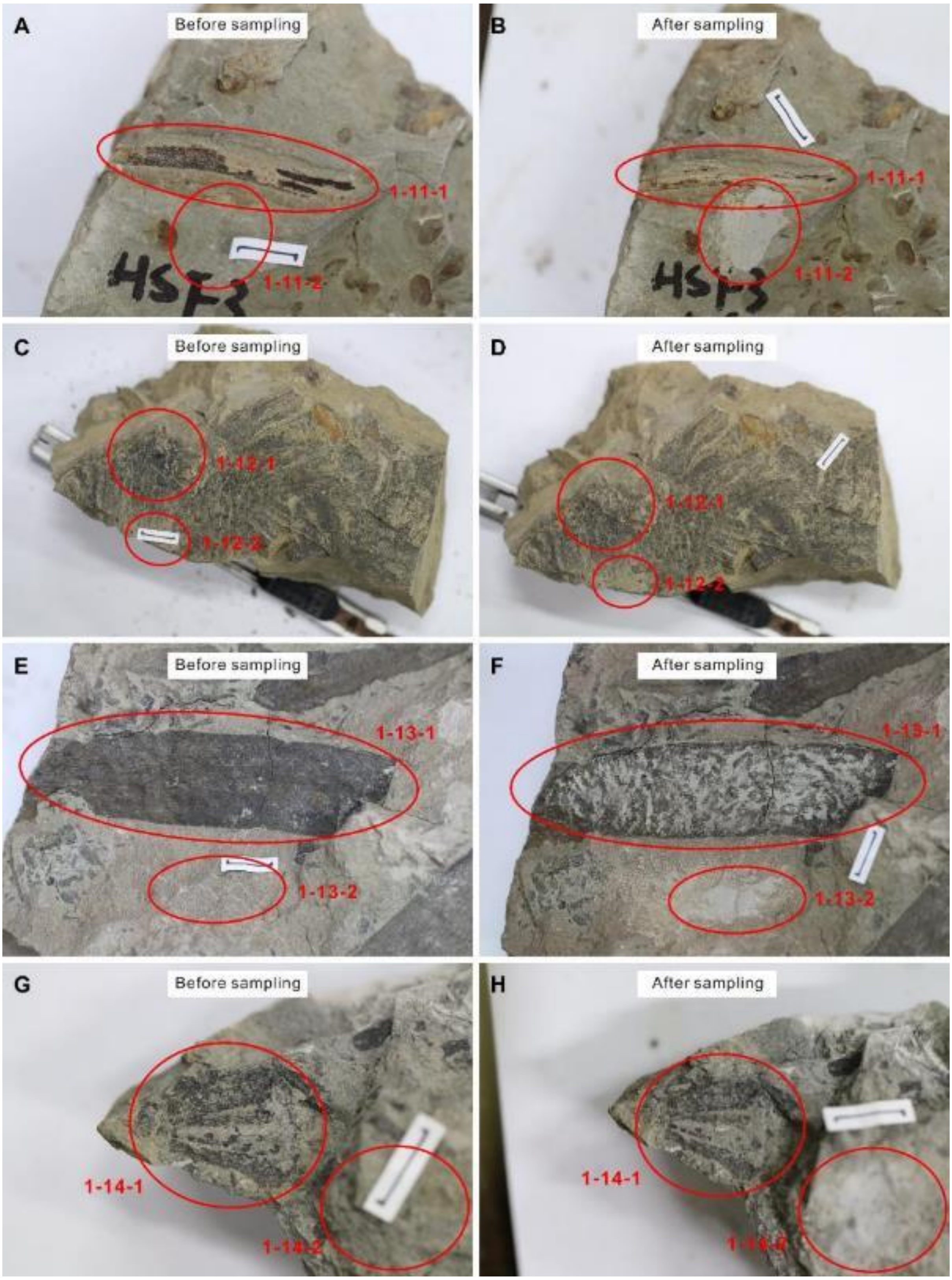

Fig. S29: Carbon isotope sampling locations on the fossils. All the plant fragment

samples have names ending with number 1, and the surrounding rock samples names end in number 2. A, C, E, G are pictures taken before sampling, and B, D, F, H are taken after sampling. A. 1-11-1 is a *Neocalamites* trunk; C. 1-12-1 is a *Lepacyclotes* sporophyll with in-situ megasporangium, the sample mainly come from the megasporangium; E. 1-13-1 is a *Pelourdea* (*Yuccites*) leaf; G. 1-14-1 is a *Lepacyclotes* sporophyll. All scale bars are 1cm. Pictures by Zhen Xu.

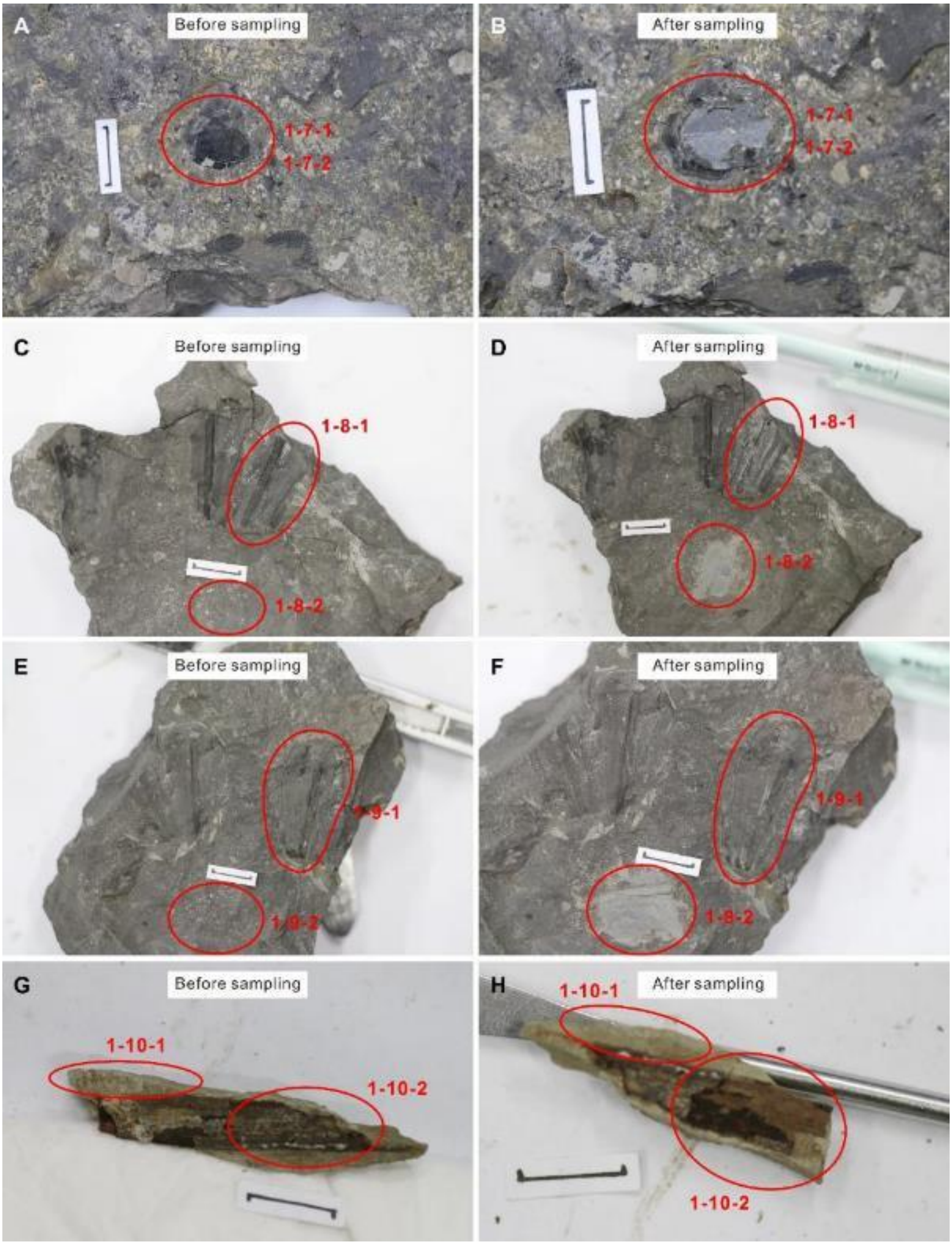

Fig. S30: Carbon isotope sampling locations on the fossils. All the plant fragment samples have names ending with 1, surrounding rock samples named end in number 2. A, C, E, G are pictures taken before sampling, and B, D, F, H are taken after sampling. A. 1-7-1 is a *Pleuromeia* sporophyll; C. 1-8-1 is a *Lepacyclotes* sporopyhll; E. 1-9-1 is a *Lepacyclotes* sporophyll; G. 1-10-1 is a *Neocalamites* trunk. All scale bars are 1cm. Pictures by Zhen Xu.

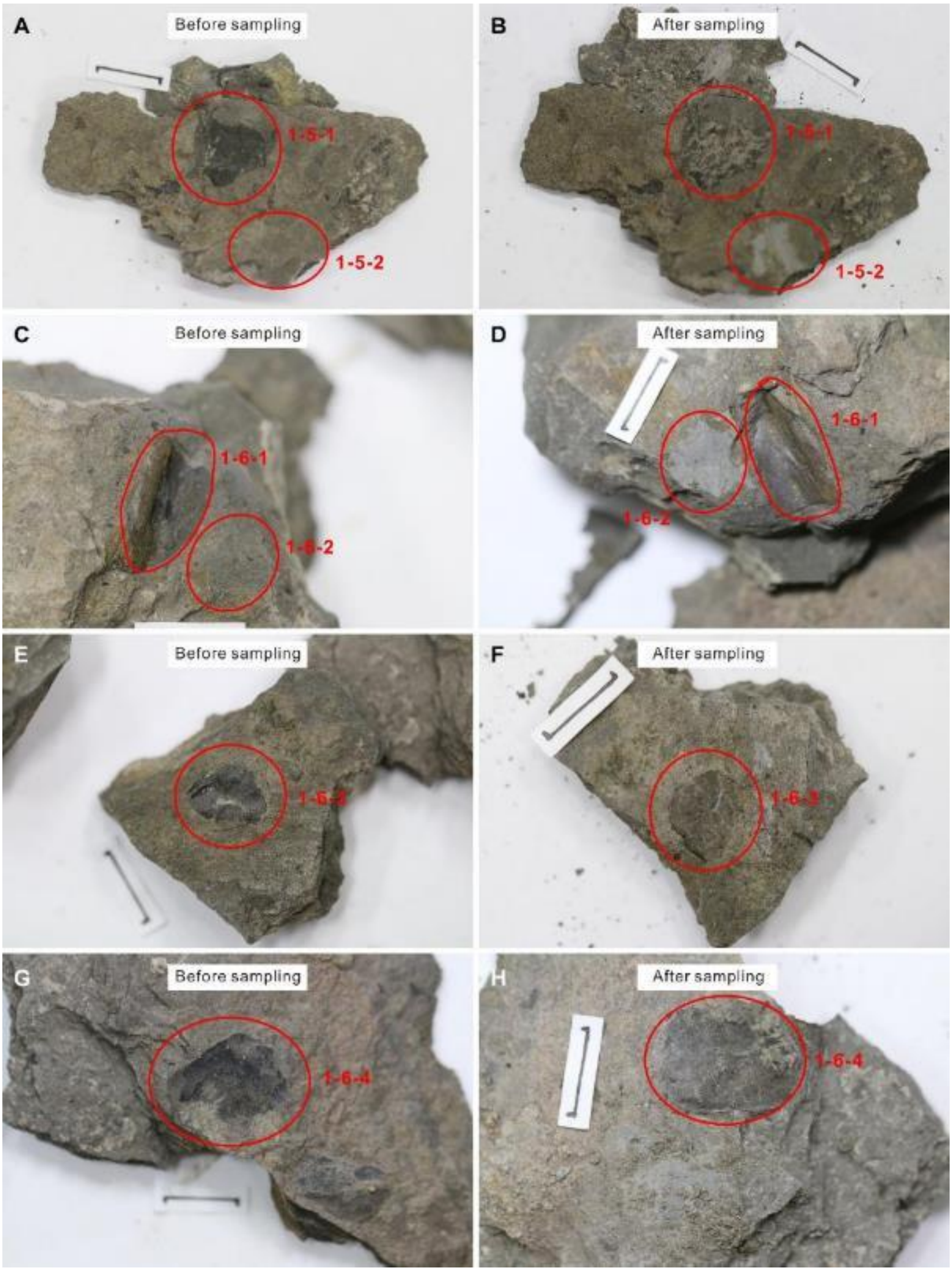

Fig. S31: Carbon isotope sampling locations on the fossils. All the plant fragment samples have names ending with 1, and the surrounding rock samples names end in number 2. A, C, E, G are pictures taken before sampling, and B, D, F, H are taken after sampling. A. 1-5-1 is a *Pleuromeia* sporophyll; C. 1-6-1 is a *Pleuromeia* sporophyll; E. 1-6-3 is an unknown plant cuticle; G. 1-6-4 is an unknown cuticle. All scale bars are

1cm. Pictures by Zhen Xu.

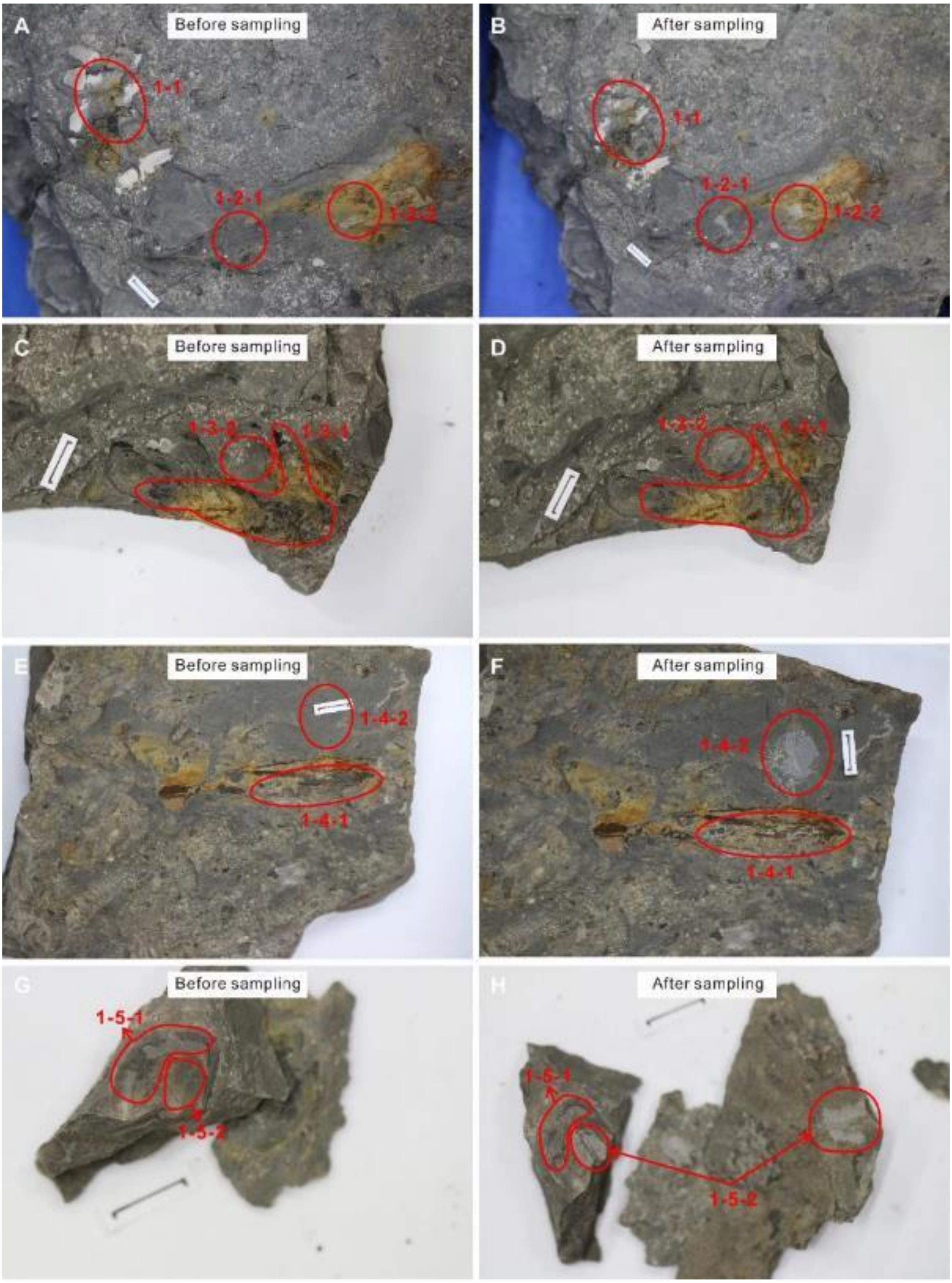

Fig. S32: Carbon isotope sampling locations on the fossils. All plant fragment samples with names ending with 1, and surrounding rock samples names ending with number 2. A, C, E, G are pictures taken before sampling, and B, D, F, H are taken after

sampling. A. 1-1 is a *Pleuromeia* sporophyll, 1-2-1 is a *Pleuromeia* trunk; C. 1-3-1 is a *Pleuromeia* sporophyll; E. 1-4-1 is a *Pleuromeia* trunk; G. 1-5-1 is a *Pleuromeia* sporophyll. All scale bars are 1cm. Pictures by Zhen Xu.

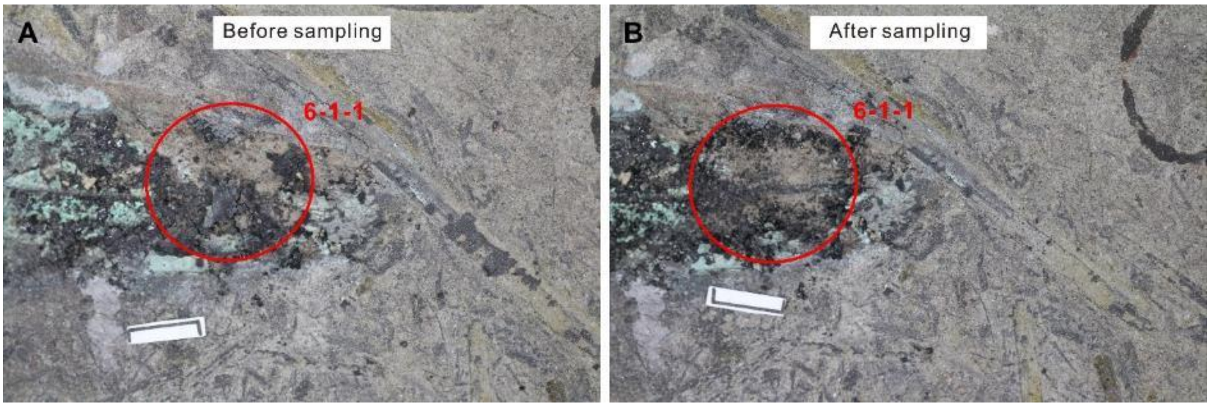

Fig. S33: Carbon isotope sampling locations on the fossils. All the plant fragment samples names end with 1, surrounding rock samples names end with 2. A is picture taken before sampling, and B is taken after sampling. A. 6-1-1 is giant unknown seed. All scale bars are 1cm. Pictures by Zhen Xu.

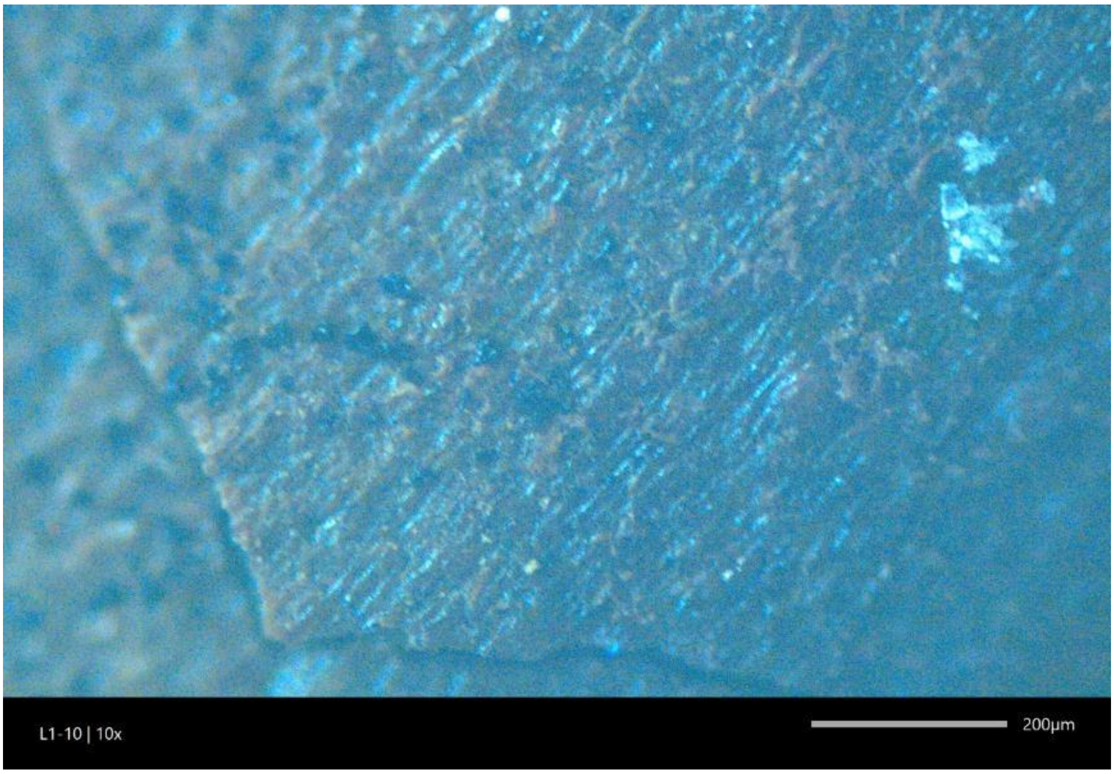

Fig. S34: Epidermal cell-like structure on the Permian-Triassic transitional Lycophyte *Tomiostrobus* L1. Picture is taken under fluorescence microscope. Pictures by Zhen Xu.

Table S1. History of nomenclature in *Tomiostrobus*, *Lepacyclotes* and *Annalepis*

Date	Author	**Genera**/eliminated genera	Age	Area	Taxonomy conclusions in these reference for the Triassic lycophytes
1856	Emmons[82]	***Lepacyclotes*** Emmons	T3	Australia	Reproductive organ of Araucariaceae
1910	Fliche[83]	***Annalepis*** Fliche	T2–3	France	Synphyllodium of *Araucarites*
1936	Neuburg[84]	***Tomiostrobus*** Neuburg	T1	Russia	Reproductive organ of Araucariaceae
1979	Ye Meina[81]	***Annalepis*** Fliche	T2	South China	Lycopsida, Lepidodendrales
1983	Grauvogel-Stamm[85]	***Annalepis*** Fliche	T2	France	Unknown family, Lepidodendrales
1991	Wang Ziqiang[86]	***Annalepis*** Fliche	T2	North China	Isoetaceae, *Isoëtes*
1995-2000	Meng Fansong[29,87–90]	***Annalepis*** Fliche, Grauvogel-Stamm =*Tomiostrobus* Neuburg	T2	South China	Paleozoic *Sigillaria* evolved into Triassic *Annalepis* and *Pleuromeia*. *Annalepis brevicystis* evolved to recent *Isoëtes*
1997	Retallack[15]	***Tomiostrobus*** Neuburg	T1	Australia	*Isoëtes* appeared in Early Triassic and continued to recent, *Tomiostrobus* evolved from *Isoëtes* then extinct in Early Triassic and evolved into *Lepacyclotes* which extinct in Late Triassic. *Pleuromeia* evolved from *Isoëtes* and extinct in Middle Triassic
		Lepacyclotes Emmons=*Annalepis* Fliche	T2–3		
2010	Yu et al.[30]	***Annalepis*** Fliche, Grauvogel-Stamm, Meng	T1	South China	Early and Middle Triassic lycopod sporophyll are all *Annalepis*, and keep *Annalepis* as morphotype
2010, 2015	Kustatscher et al.[26,91]	***Lepacyclotes*** Emmons =*Annalepis* Fliche, Grauvogel-Stamm	T2	Italy	Delete *Annalepis* and use *Lepacyclotes*
2012	Naugolnykh[13]	***Tomiostrobus*** Neuburg, Retallack	T1	Russia	*Tomiostrobus* in the Early Triassic evolved into *Annalepis* in the Middle Triassic
2020	Feng et al.[25]	***Tomiostrobus*** Neuburg, Retallack, Naugolnykh =*Annalepis* Fliche	T1–2	South China	All Early and Middle Triassic lycopod sporophylls in South China are one species of *Tomiostrobus sinensis*
2023	Deng et al.[28]	***Lepacyclotes*** Emmons, Kustatscher =*Annalepis* Fliche, Grauvogel-Stamm, Meng =*Tomiostrobus*	T1–2	North China	All Early and Middle Triassic lycopod sporophyll are different species of *Lepacyclotes*

		Neuburg, Retallack, Naugolnykh, Feng			
2025	This paper	***Tomiostrobus*** Neuburg, Retallack, Naugolnykh =*Annalepis* Fliche, Grauvogel-Stamm, Yu	T1	South China	Delete *Annalepis*, Early Triassic sporophylls are *Tomiostrobus* which evolved to recent *Isoëtes* and extinct after the Early Triassic, Middle Triassic ones are *Lepacyclotes* extinct in Late Triassic
		Lepacyclotes=*Annalepis* Fliche, Grauvogel-Stamm, Meng	T2		

Table S2: Summary score of each Principal Component (PC) in Figure 2, Figure S1 and Figure S2

PC	Figure 2&S2-A		Figure 2&S2-B		Figure 2&S2-C		Figure 2&S2-D		Figure 2&S2-E		Figure 2&S2-F	
	Eigenvalue	Variance (%)	Eigenvalue	Variance (%)	Eigenvalue	Variance (%)	Eigenvalue	Variance (%)	Eigenvalue	Variance (%)	Eigenvalue	Variance (%)
1	2.64	21.08	3.40	38.09	2.80	25.52	1.88	18.49	1.60	14.20	2.16	13.84
2	1.54	12.34	0.97	10.85	1.17	10.67	1.12	11.03	1.08	9.60	1.71	10.95
3	0.82	6.59	0.85	9.51	0.89	8.07	0.90	8.87	0.87	7.79	1.12	7.18
4	0.74	5.90	0.57	6.39	0.72	6.57	0.62	6.12	0.73	6.28	0.96	6.15
5	0.58	4.66	0.41	4.62	0.61	5.59	0.54	5.30	0.59	5.27	0.69	4.40
6	0.48	3.86	0.38	4.25	0.48	4.36	0.47	4.67	0.56	5.00	0.58	3.74
7	0.41	3.31	0.31	3.49	0.44	4.00	0.44	4.36	0.49	4.33	0.56	3.61
8	0.40	3.17	0.30	3.34	0.42	3.79	0.36	3.51	0.40	3.54	0.48	3.09
9	0.38	3.02	0.22	2.52	0.34	3.12	0.33	3.27	0.39	3.45	0.42	2.67
10	0.33	2.60	0.22	2.44	0.30	2.75	0.32	3.17	0.34	3.00	0.41	2.61

Table S3. Loading plot of each character in all lycopods PCA result (Fig. 2B). Find the character description based on the number in the supplementary file 1.

Character No.	Principle Components						
	PC 1	PC 2	PC 3	PC 4	PC 5	PC 6	PC 7
Ch-1	-0.03245	0.16608	-0.11304	-0.04388	-0.0654	-0.0034	-0.04797
Ch-2	0.03245	-0.16608	0.11304	0.04388	0.065404	0.003403	0.04797
Ch-3	0.10164	-0.24158	-0.08459	0.082058	0.084209	-0.06466	0.072251
Ch-4	-0.08131	0.093558	0.21782	-0.03456	-0.05379	0.056778	-0.03258
Ch-5	-0.02034	0.14802	-0.13323	-0.0475	-0.03042	0.007879	-0.03967
Ch-6	0.03245	-0.16608	0.11304	0.04388	0.065404	0.003403	0.04797
Ch-7	-0.03245	0.16608	-0.11304	-0.04388	-0.0654	-0.0034	-0.04797
Ch-8	0.019751	-0.14454	0.13102	0.046092	0.028704	-0.01131	0.040207
Ch-9	-0.02041	0.14055	-0.12728	-0.04533	-0.02817	0.015826	-0.02871
Ch-11	0.28326	-0.03277	0.048991	-0.08446	-0.00886	0.060359	0.1672
Ch-12	-0.28326	0.032771	-0.04899	0.084457	0.008859	-0.06036	-0.1672
Ch-16	-0.00374	0.023903	-0.01986	-0.00733	-0.0016	-2.85E-05	-0.02054
Ch-17	-0.10025	0.24019	0.081133	-0.08187	-0.08321	0.061595	-0.07439
Ch-18	0.020771	-0.13931	0.11476	0.038703	0.022394	-0.00586	0.048702
Ch-19	0.061576	0.034181	0.27371	-0.16778	0.041575	-0.00418	0.080248
Ch-20	0.047234	0.066485	-0.08305	0.11477	-0.12119	-0.0608	-0.02082
Ch-21	-0.05637	-0.13176	-0.13354	0.057582	0.095992	0.13676	0.047343
Ch-22	-0.01854	0.015141	-0.02327	0.017753	-0.03606	0.002064	-0.04869
Ch-23	0.093984	0.059216	0.013214	0.04077	0.26863	0.032091	0.089288
Ch-24	-0.00488	-0.0004	0.16696	-0.13232	-0.02374	-0.14892	0.064547
Ch-25	0.011982	0.049351	-0.04278	0.00046	0.015773	0.052676	0.023642

Ch-26	-0.03098	-0.08584	-0.12045	0.12354	-0.06459	0.12814	-0.10337
Ch-27	0.022535	0.035425	-0.00501	0.007706	0.066124	-0.03032	0.01395
Ch-28	0.012369	0.11441	-0.06524	-0.04429	0.025581	-0.03748	0.060644
Ch-29	0.15965	-0.04408	0.020256	-0.21598	-0.0085	0.22301	-0.17521
Ch-30	-0.084	-0.05219	0.005773	0.10593	0.057713	0.048347	0.13577
Ch-31	-0.06717	-0.02606	0.019039	0.063918	-0.04247	-0.13603	0.029719
Ch-32	-0.01807	0.000307	0.022709	0.025949	-0.0141	-0.03825	-0.01707
Ch-33	-0.00094	0.005301	-0.0054	0.063813	-0.0139	-0.05793	-0.03278
Ch-34	0.007437	0.032449	-0.00503	-0.00778	0.031649	-0.02766	0.029983
Ch-35	0.078677	-0.00783	-0.03763	-0.25343	0.11741	-0.23501	-0.20792
Ch-36	0.007741	-0.0515	-0.00032	0.10515	-0.05382	0.47232	0.1236
Ch-37	-0.05236	0.004244	0.005159	0.063215	-0.0182	-0.0865	0.069685
Ch-38	-0.01352	-0.00684	-0.01727	0.041278	-0.01764	-0.0518	-0.00311
Ch-39	-0.01283	0.002373	0.011312	0.035118	-0.03068	-0.04798	-0.00467
Ch-40	-0.01866	0.02455	0.043156	0.01317	-0.02548	-0.02243	-0.00918
Ch-41	0.064647	0.11255	-0.00871	-0.02601	-0.1871	0.046099	0.23189
Ch-42	-0.05358	-0.10686	0.019448	-0.05677	0.12634	0.11443	-0.14648
Ch-43	-0.00937	-0.00474	-0.01455	0.056601	0.053178	-0.10895	-0.05688
Ch-44	-0.0004	-0.00198	0.001048	0.025518	0.007886	-0.05628	-0.0242
Ch-45	0.006269	0.017963	-0.0152	-0.03574	0.013398	-0.04479	0.030745
Ch-46	0.070552	-0.01294	-0.0109	-0.2255	-0.01888	-0.18358	-0.08918
Ch-47	0.038665	-0.00444	0.009928	-0.01959	0.10249	0.29483	-0.09065
Ch-48	0.016852	-0.01395	-0.01619	0.05576	-0.03529	0.13391	0.032328
Ch-49	-0.00941	-0.01185	0.012131	0.043402	0.006501	0.018578	0.045262
Ch-50	-0.0743	-0.01042	0.01251	0.093104	-0.01156	-0.10027	0.095578
Ch-51	-0.03005	-0.00034	-0.00466	0.048906	-0.02908	-0.07001	0.005219

Ch-52	-0.01749	0.037133	0.013133	0.040746	-0.02814	-0.04619	-0.02872
Ch-53	0.031538	-0.17024	0.11255	0.043705	0.057907	0.000955	0.057393
Ch-54	-0.15276	-0.14473	0.088564	-0.19511	0.18392	-0.02084	0.15077
Ch-55	0.14575	-0.03097	0.059349	0.11972	-0.04604	0.041236	-0.07319
Ch-56	0.040215	0.001952	-0.03941	0.11628	-0.08268	-0.02756	-0.01697
Ch-57	-0.00307	0.012404	-0.00152	0.001608	0.003915	0.009664	-0.01373
Ch-58	0.27985	0.10533	-0.11914	-0.04036	0.050876	-0.03312	-0.06454
Ch-59	0.24003	-0.03301	0.024203	-0.08011	0.16526	-0.05747	-0.06521
Ch-60	0.039827	0.13835	-0.14334	0.039746	-0.11438	0.024348	0.000671
Ch-61	-0.16933	-0.17747	0.1342	-0.15574	-0.13694	-0.01731	0.10209
Ch-62	0.039504	0.009015	-0.03819	0.091663	0.058039	-0.04941	-0.23903
Ch-63	0.051383	0.066173	-0.08205	0.049134	-0.11175	0.035512	0.005776
Ch-64	0.078446	0.10228	-0.01396	0.014943	0.19065	0.031202	0.13116
Ch-65	0.009425	0.012021	0.007655	0.0236	0.096062	-0.04634	0.047625
Ch-66	-6.44E-05	0.020228	0.039406	0.063241	-0.01747	-0.04389	-0.07271
Ch-67	0.064459	0.053562	0.12295	0.046669	-0.06709	-0.00638	0.063735
Ch-68	-0.07481	-0.08659	-0.16904	-0.13504	-0.01434	0.10344	-0.04062
Ch-69	-0.11467	0.002784	-0.1767	-0.16113	-0.01366	0.12638	0.1484
Ch-70	-0.04606	-0.07624	0.18603	-0.05711	-0.12963	-0.10821	-0.2549
Ch-71	0.16073	0.073452	-0.00933	0.21824	0.14329	-0.01817	0.10651
Ch-72	-0.13852	-0.07459	-0.25952	-0.22472	-0.0338	0.0475	0.13752
Ch-73	0.16723	0.008871	0.026864	0.22734	-0.13849	-0.00793	-0.03415
Ch-74	-0.02871	0.065719	0.23266	-0.00262	0.17229	-0.03957	-0.10337
Ch-75	0.010813	-0.1576	0.093557	-0.01976	-0.26466	0.081417	-0.01456
Ch-76	-0.0016	0.14584	-0.11475	0.016736	0.26213	-0.08475	0.012079
Ch-77	-0.02266	0.14754	-0.12545	-0.04393	-0.02718	0.020506	-0.03139

Ch-78	0.016435	0.018356	-0.00671	0.050893	0.27191	-0.09498	0.047349
Ch-79	-0.2179	-0.128	0.014315	0.1225	-0.03601	-0.03218	-0.08873
Ch-80	0.22413	-0.03789	0.11784	-0.12946	-0.20872	0.10665	0.072769
Ch-81	0.048334	0.14864	-0.16148	0.12671	-0.10676	-0.07848	-0.07968
Ch-82	0.17644	0.049323	0.044938	0.11505	0.10948	0.076327	0.12149
Ch-83	0.003298	-0.00459	-0.0266	0.092913	0.016936	-0.07351	-0.08001
Ch-84	0.026255	0.006633	0.009555	0.018163	0.013755	0.03965	0.039386
Ch-85	-0.02894	-0.02509	-0.00189	0.005318	-0.01943	-0.01817	-0.00648
Ch-88	0.069963	-0.22846	-0.12582	0.049343	0.048695	-0.03611	0.025594
Ch-89	-0.06996	0.22846	0.12582	-0.04934	-0.0487	0.036105	-0.02559
Ch-90	0.074916	-0.08545	-0.21355	0.02618	0.059087	-0.03527	0.025189
Ch-91	-0.07492	0.085451	0.21355	-0.02618	-0.05909	0.03527	-0.02519
Ch-92	0.039561	0.025956	0.009289	0.02102	0.036528	-0.01518	0.060126
Ch-93	0.15352	0.070819	-0.033	-0.14876	-0.17078	-0.08457	0.11481
Ch-94	0.008582	-0.02036	-0.00873	-0.01809	0.12969	0.21984	-0.32726
Ch-95	-0.08204	-0.04667	0.000479	0.032981	0.061559	0.060884	0.089335
Ch-96	-0.08769	-0.04676	-0.00674	0.096555	-0.04195	-0.14071	0.061524
Ch-97	-0.03194	0.017005	0.038706	0.016292	-0.01505	-0.04028	0.001465
Ch-98	0.040116	-0.00933	0.004293	-0.06449	0.042558	-0.10192	0.053869
Ch-99	0.16154	-0.08541	-0.03393	-0.10511	-0.04	0.04732	-0.0808
Ch-100	-0.14129	0.030684	-0.06001	0.17023	0.046529	0.095077	-0.00108
Ch-101	-0.06008	0.066777	0.092228	0.003795	-0.04805	-0.03841	0.020932
Ch-102	0.1178	0.022351	-0.01496	-0.09999	-0.01649	-0.16895	0.1496
Ch-103	0.11574	0.003959	-0.02809	-0.09779	-0.01623	0.12024	-0.13182
Ch-104	-0.03549	-0.03649	-0.02533	0.027039	0.034679	0.10576	-0.10283
Ch-105	-0.04671	-0.01379	0.014671	0.052865	0.040749	0.0656	-0.00031

Ch-106	-0.0401	-0.00471	-0.02364	0.028768	0.006018	0.00715	0.028431
Ch-107	-0.06622	-0.00723	0.009187	0.058119	-0.00451	-0.06661	0.052784
Ch-108	-0.0232	0.009105	0.016852	0.021608	-0.01022	-0.04236	0.016788
Ch-109	-0.02307	0.028227	0.053322	0.002895	-0.02696	-0.01748	-0.00813
Ch-110	0.009802	0.24666	0.11355	-0.04793	0.1606	0.13295	0.084798
Ch-111	0.00811	-0.2683	-0.14625	0.043445	-0.1261	-0.12234	-0.0779
Ch-112	-0.01791	0.021637	0.032708	0.00448	-0.03449	-0.01061	-0.0069
Ch-113	-0.16455	0.054747	0.13168	0.003312	0.000942	0.10782	0.01857
Ch-114	0.049969	-0.11358	-0.10303	-0.20314	0.101	-0.03831	-0.14624
Ch-115	0.098169	0.037589	-0.01155	0.14664	-0.11289	-0.01753	0.11729
Ch-116	0.01747	0.023254	-0.01788	0.051022	0.012199	-0.04848	0.013665
Ch-117	-0.15509	-0.0303	-0.13691	-0.22728	0.022324	0.028974	0.18706
Ch-118	0.10314	0.021131	0.15766	0.11799	0.040903	0.018758	-0.20621
Ch-119	0.030642	0.002939	-0.00251	0.040434	-0.03464	0.009968	0.025384
Ch-120	0.016809	0.005619	-0.00997	0.033164	-0.01747	-0.02811	0.010379
Ch-121	0.004501	0.000613	-0.00827	0.035695	-0.01112	-0.02959	-0.01662
Ch-122	-0.03961	0.077295	0.079698	-0.03347	-0.053	-0.00351	0.006575
Ch-123	-0.13438	0.061475	-0.01615	-0.00061	0.23017	0.036962	0.002776
Ch-124	0.11795	-0.11311	-0.04525	-0.031	-0.09708	0.094994	-0.08237
Ch-125	0.045549	-0.01603	-0.01249	0.033932	-0.05981	-0.07932	0.045735
Ch-126	0.003553	-0.00788	-0.00498	0.025782	-0.01491	-0.0235	0.01527
Ch-127	0.006943	-0.00175	-0.00082	0.005365	-0.00538	-0.02563	0.012016

Table S4. Recommended Triassic Isoetales lycopod sporophyll nomenclature. Taxa in red and bold are extant; these should not be synonymized with fossil sporophyll taxa as in each case the taxon is distinguished and diagnosed primarily on features of other parts of the plant as well as their sporophylls. Name in brackets are junior synonyms. Fossil taxa denoted in open nomenclature as "sp." are referred to by their source location because in several instances individual genera include multiple examples of "sp.", but with each coming from a different source region and study. Taxa marked with * are highlighted because they are either based on small sample sizes, information taken from sketches rather than photos, or the fossils are poorly preserved, for which their reliability is questioned. Corrected species in this study have distinct morphospace in the PCA and NNA, while morphotypes of each species have divergence and overlapping within the concluded species. Detailed information on each taxon is presented in Supplementary file 2 based on the tracking number which allows information to be correlated across different documents.

**Corrected genera**	**Corrected species in this study**		**Before correction (junior synonyms and local varieties)**	**Reference**	**Track number**
	**Species**	**Morphotypes**			
Lepacyclotes	*Lepacyclotes zeilleri*_NC	*Lepacyclotes zeilleri*_NC	*Isoetes* (*Annalepis*) *ermayinensis*_North China_Wang	Wang et al.[92]	31
			*Lepacyclotes zeilleri*_North China_Deng	Deng et al.[28]	480
			Lepacyclotes kirchneri	Bauer et al.[93]	221
	Lepacyclotes brevicystis	*Lepacyclotes brevicystis*	*Annalepis brevicystis*	Meng et al.[29]; This work	11
	Lepacyclotes circularis (*zeilleri*)_EU	*Lepacyclotes ermayinensis*	*Lepacyclotes ermayinensis**	Retallack[15]	218
		Lepacyclotes convexus	*Lepacyclotes* (*Araucarites, Tomiostrobus*) *convexus**	Brik[94]; Sadovnikov[95]; Retallack[15]	217
		Lepacyclotes circularis (*zeilleri*)_EU	*Lepacyclotes* sp.3_German*	Bauer et al.[93]	224
			*Lepacyclotes circularis**	Emmons[82]; Retallack[15]	216
			Annalepis (*Cylostrobophyllum*) *zeilleri*_EU	Fliche[83]; Grauvogel-Stamm and Duringer[85]; Kustatscher et al.[26]	227
			Lepacyclotes sp.2_German*	Bauer et al.[96]	222

	*Lepacyclotes zeilleri*_SC	*Lepacyclotes* sp.1_DE	*Annalepis* sp.1_German*	Grauvogel-Stamm and Lugardon[14]	63
		*Lepacyclotes zeilleri*_SC	*Annalepis kryshtofovichii**	Naugolnykh[13]	34
			Annalepis latiloba	Meng et al.[29]; This work	43
			*Annalepis zeilleri*_South China	Meng et al.[29]; This work	126
			Annalepis angusta	Meng et al.[29]; This work	7
		Lepacyclotes sp.2_SC	*Annalepis* sp.2_South China	This work	76
	Lepacyclotes ordoensis	*Lepacyclotes ordoensis*	*Lepacyclotes ordoensis*	Deng et al.[28]	474
Tomiostrobus	*Tomiostrobus zeilleri*	*Tomiostrobus angusta*	*Annalepis angusta*	Yu et al.[30]	309
		Tomiostrobus sp.2_SC	*Annalepis* sp.2_South China	Yu et al.[6]	419
		Tomiostrobus zeilleri	*Annalepis zeilleri*	Yu et al.[30]	449
			Annalepis sp.1_South China	Yu et al.[6]	395
	Tomiostrobus migayi	*Tomiostrobus migayi*	*Tomiostrobus migayi*	Sadovnikov[95]; Retallack[15]	345
			Tomiostrobus sp.1_Xinjiang	Yu et al., unpublished data	453
		Tomiostrobus brevicystis	*Annalepis brevicystis*	Yu et al.[30]	324
		Tomiostrobus sp.2_Xinjiang	*Tomiostrobus* sp.2_Xinjiang	Yu et al., unpublished data	459
			Tomiostrobus sp.3_Xinjiang	Yu et al., unpublished data	461
	Tomiostrobus radiatus	*Tomiostrobus belozerovii*	*Tomiostrobus bulbosus**	Sadovnikov[95]	332
			*Tomiostrobus fusiformis**	Sadovnikov[95]	334
			*Tomiostrobus belozerovii**	Sadovnikov[95]	311
			*Tomiostrobus convexus**	Sadovnikov[95]	333
		Tomiostrobus polaris	*Tomiostrobus* (*Takhtajanodoxa*) *mirabilis**	Snigirevskaya[97]; Retallack[15]	347
			Tomiostrobus (*Selaginellites*) *polaris**	Lundblad[98]; Retallack[15]	348
			Tomiostrobus (*Pleuromeia*) *taimyrica**	Sadovnikov[95]; Retallack[15]	422

			*Tomiostrobus gorskyii**	Grauvogel-Stamm and Lugardon[14]	336
		Tomiostrobus radiatus	*Tomiostrobus radiatus*	Dobruskina[99]; Naugolnykh[13]	352
	Tomiostrobus australis	*Tomiostrobus australis*	*Skilliostrobus australis**	Ash[100]; Sadovnikov[95]; Retallack[15]	310
Isoetes* and *Isoetites		*Isoetites rolandii*	*Isoetites rolandii*	Ash and Pigg[101]	211
		Isoetes anatolica	***Isoetes anatolica***	Prada and Rolleri[102]	192
		Isoetes sinensis	***Isoetes sinensis***	Qin et al.[103]	201
			Isoetites choffati	Skog and Hill[104]; Teixeira[105]	202
	Isoetites horridus	*Isoetites horridus*	*Isoetites horridus*	Hickey[106]	209
	Isoetites daharensis	*Isoetites daharensis*	*Isoetites daharensis*	Barale[107]	205
	Isoetites serratus	*Isoetites serratus*	*Isoetes* (*Cylomeia*) *beestonii**	Retallack[15]	193
			Isoetites serratus	Brown[108]	212
		Isoetites sp.1_Spain	*Isoetites* sp.1_Spain*	Villanueva-Amadoz et al.[109]	214
			Isoetes serracarajensis	Pereira et al.[110]	200
			Isoetes cangae	Pereira et al.[110]	194
			Isoetes orientalis	Liu et al.[111]	199
			Isoetes lacustris	Naugolnykh[13]	198
			Isoetes innae	Naugolnykh[112]	196
		Isoetes georgiana	***Isoetes georgiana***	Brunton and Britton[113]	195
Pleuromeia	*Pleuromeia sternbergii*_NC	*Pleuromeia sternbergii*_NC	*Pleuromeia sternbergii*_North China	Wang et al.[114]	294
	*Pleuromeia rossica*_RU, EU and NC	*Pleuromeia rossica*_RU, EU and NC	*Pleuromeia obovata*	Deng et al.[115]	466
			*Pleuromeia jiaochengensis**	Wang[86]	266
			*Pleuromeia rossica*_RU and EU	Neuburg[84]; Lugardon et al.[116]	277
	*Pleuromeia sternbergii*_DE	*Pleuromeia rossica*_NC	*Pleuromeia rossica*_North China	Wang[86]	283
			Pleuromeia marginulata	Meng et al.[29]	269

		Pleuromeia marginulata	*Pleuromeia* (*Stigmatodendron, Cylomeia, Gregicaulis*) *dubia**	Retallack[15]	258
		*Pleuromeia sternbergii*_DE	*Pleuromeia sternbergii*_German	Grauvogel-Stamm[117]	290
		Pleuromeialean sp.1_AN	*Pleuromeialean* sp.1_Antarctica (vegetative leaf *Mesenteriophyllum serratum*) *	Bomfleur et al.[117]	296
			*Pleuromeia hataii**	Retallack[15]	259
	Pleuromeia rossica 2_RU	*Pleuromeia rossica* 2_RU	*Lycomeia rossica**	Retallack[15]	253
	Pleuromeia hunanensis	*Pleuromeia hunanensis*	*Pleuromeia sanxiaensis*	Meng et al.[29]	286
			Pleuromeia hunanensis	Meng et al.[29]	261
	Pleuromeia shaolinii	*Pleuromeia shaolinii*	*Pleuromeia shaolinii*	Zhang et al.[119]	287
Cylostrobus	*Cylostrobus ornatus*	*Cylostrobus ornatus*	*Cylostrobus ornatus*	Retallack[15]	188
	Cylostrobus indicus	*Cylostrobus indicus*	*Cylostrobus* (*Araucarites, Conites, Lycopodiopsis, Pleuromeia*) *indicus*	Lele[120]; Retallack[15]	187
			Cylostrobus (*Arucarites, Tomiostrobus, Caulopteris, Pleuromeia, Cylomeia*) *sydneyensis*	Helby and Martin[121]; Retallack[15]	189
Lycostrobus	*Lycostrobus chinleanus*	*Lycostrobus chinleanus*	*Lycostrobus chinleanus*	Retallack[15]	254
	Lycostrobus scotii	*Lycostrobus scotii*	*Lycostrobus scotii**	Retallack[15]	255

Table S5. Organic carbon isotope of plant fossils from end Permian to Middle Triassic in South China. CUG is China University of Geosciences (Wuhan) and UCD is University of California, Davis.

Age	Section number	Sample number	Content description	Carbon type	δ13Corg (some samples have multiple tests)			Test time	Test location
Middle Triassic (Badong Formation)	HSF1	1-13-1	*Pelourdea* (*Yuccites*)	Carbon film	-25.46788			2023/4/27	CUG
	HSF1	1-13-2	Surrounding rock	Mixed	-28.457792	-28.486908		2023/4/28	CUG
	HSF2	1-1	*Pleuromeia* sporophyll	Cuticle	-24.641588	-24.961864		2023/4/1	CUG
	HSF2	1-2-1	*Pleuromeia* trunk	Cuticle	-25.11146	-24.68	-25.007044	2023/4/2	CUG
	HSF2	1-2-2	*Pleuromeia* trunk	Cuticle	-24.706848	-24.424724	-24.786164	2023/4/3	CUG
	HSF2	1-2-3	Surrounding rock	Mixed	-26.650592			2023/4/4	CUG
	HSF2	1-3-1	*Pleuromeia* sporophyll	Cuticle	-24.997004	-24.983952		2023/4/5	CUG
	HSF2	1-3-2	Surrounding rock	Mixed	-21.086424			2023/4/6	CUG
	HSF2	1-4-1	*Pleuromeia* trunk?	Cuticle	-24.06	-24.495004	-24.551228	2023/4/7	CUG
	HSF2	1-4-2	Surrounding rock	Mixed	-25.946788			2023/4/8	CUG
	HSF2	1-5-1	*Pleuromeia* sporophyll	Cuticle	-24.959856	-24.80022		2023/4/9	CUG
	HSF2	1-5-2	Surrounding rock	Mixed	-26.008032	-26.04	-26.16566	2023/4/10	CUG
	HSF2	1-6-1	*Pleuromeia* trunk?	Cuticle	-24.516088	-24.266092		2023/4/11	CUG
	HSF2	1-6-2	Surrounding rock	Mixed	-25.47	-25.461856	-25.464868	2023/4/12	CUG
	HSF2	1-6-3	Unknown, could be sporophyll	Cuticle	-24.412676			2023/4/13	CUG
	HSF2	1-6-4	Unknown, could be sporophyll	Cuticle	-25.280132			2023/4/14	CUG
	HSF2	1-7-1	*Pleuromeia* sporophyll	Cuticle	-25.107444	-25.167684		2023/4/15	CUG
	HSF2	1-7-2	Surrounding rock	Mixed	-25.24198	-25.531132		2023/4/16	CUG
	HSF2	1-8-1	*Lepacyclotes* sporophyll?	Carbon film	-24.17774			2023/4/17	CUG
	HSF2	1-8-2	Surrounding rock	Mixed	-25.329328			2023/4/18	CUG
	HSF2	1-9-1	*Lepacyclotes* sporophyll?	Cuticle	-24.52914	-24.465888		2023/4/19	CUG
	HSF2	1-9-2	Surrounding rock	Mixed	-25.148608	-25.07	-25.07632	2023/4/20	CUG
	HSF3	1-10-1	*Neocalamites* trunk	Cuticle	-26.028112			2023/4/21	CUG
	HSF3	1-10-2	Surrounding rock	Mixed	-26.716856			2023/4/22	CUG
	HSF3	1-11-1	*Neocalamites* trunk	Cuticle	-25.87	-25.855424	-25.917672	2023/4/23	CUG
	HSF3	1-11-2	Surrounding rock	Mixed	-26.755008			2023/4/24	CUG

	HSF4	1-12-1	Megaspores	Cuticle	-24.500024			2023/4/25	CUG
	HSF4	1-12-2	Surrounding rock	Mixed	-26.90	-26.941752		2023/4/26	CUG
	HSF4	1-14-1	*Lepacyclotes* sporophyll	Cuticle	-25.936748			2023/4/29	CUG
	HSF4	1-14-2	Surrounding rock	Mixed	-26.116464			2023/4/30	CUG
	HSF4	1-15-1	Fern or conifer trunk	Cuticle	-25.56326			2023/5/1	CUG
	HSF4	1-15-2	Surrounding rock	Mixed	-26.700792			2023/5/2	CUG
	HSF4	1-16-1	*Voltzia*	Carbon film	-25.587356	-25.719884		2023/5/3	CUG
	HSF4	1-16-2	Surrounding rock	Mixed	-25.910644			2023/5/4	CUG
	HSF4	1-17-1	Conifer trunk	Carbon film	-26.621476			2023/5/5	CUG
	HSF4	1-17-2	Surrounding rock	Mixed	-25.981928			2023/5/6	CUG
	HSF4	1-18-1	Seed	Cuticle	-23.39	-23.436788		2023/5/7	CUG
	HSF4	1-18-2	Surrounding rock	Mixed	-24.833352			2023/5/8	CUG
	HSF4	1-19-1	Conifer trunk	Cuticle	-24.29			2023/9/6	CUG
	HSF4	1-19-2	Surrounding rock	Mixed	-25.95			2023/9/7	CUG
	HSF4	1-20-1	*Lepacyclotes* sporophyll	Cuticle	-25.61			2023/9/11	CUG
	HSF4	1-20-2	Surrounding rock	Mixed	-26.26			2023/9/12	CUG
	HSF4	1-21-1	*Lepacyclotes* sporophyll?	Cuticle	-23.35			2023/10/13	CUG
	HSF4	1-21-2	Surrounding rock	Mixed	-24.67			2023/10/14	CUG
	HSH1	4-1-1	Megaphyllous leaf with *Spirorbis*	Carbon film	-22.54			2023/9/15	CUG
	HSH1	4-1-2	Surrounding rock	Mixed	-23.87			2023/9/16	CUG
	HSH1	4-2-1	Unknown trunk	Cuticle	-24.24			2023/9/18	CUG
	HSH1	4-2-2	Surrounding rock	Mixed	-25.41			2023/9/19	CUG
	HSH1	4-3-1	*Lepacyclotes* sporophyll?	Cuticle	-23.24			2023/9/22	CUG
	HSH1	4-3-2	Surrounding rock	Mixed	-25.38			2023/9/23	CUG
	HSH1	4-4-1	*Neocalamite*stem	Carbon film	-26.53			2023/9/26	CUG
	HSH1	4-4-2	Surrounding rock	Mixed	-25.37			2023/9/27	CUG
	HSH1	4-5-1	*Lepacyclotes* sporophyll?	Cuticle	-23.21			2023/9/29	CUG
	HSH1	4-5-2	Surrounding rock	Mixed	-25.37			2023/9/30	CUG
	HSH1	4-6-1	Megaphyllous leaf with *Spirorbis* (another one)	Carbon film	-22.90			2023/10/3	CUG
	HSH1	4-6-2	Surrounding rock	Mixed	-25.27			2023/10/4	CUG
	HSH2	6-1-1	Giant seed	Cuticle	-25.51			2023/10/9	CUG

	HSH2	6-1-2	Surrounding rock	Mixed	-25.06			2023/10/10	CUG
	HSH2	6-1-4	*Neocalamite*	Cuticle	-24.00			2023/10/12	CUG
	HSF3	L3-1	*Lepacyclotes*	Carbon film	-26.342689			2025/4/21	UCD
Early Triassic Olenekian (Lingwen Formation)	Lingwen	2-4-1	Conifer trunk	Cuticle	-24.591388			2023/5/15	CUG
	Lingwen	2-1-1	Unknown trunk	Cuticle	-24.43878			2023/5/9	CUG
	Lingwen	2-1-2	Surrounding rock	Mixed	-23.97192			2023/5/10	CUG
	Lingwen	2-2-2	Surrounding rock	Mixed	-23.559276			2023/5/12	CUG
	Lingwen	2-5-1	Unknown leaf	Cuticle	-23.03318			2023/5/16	CUG
	Lingwen	2-2-1	Seed	Cuticle	-22.597444			2023/5/11	CUG
Early Triassic late Induan (Feixianguan Formation)	Pojiao	10-1-1	*Voltzia*	Carbon film	-26.0473172			2024/7/30	CUG
	Pojiao	10-1-2	Surrounding rock	Mixed	-25.9650861			2024/7/30	CUG
	Pojiao	10-2-1	*Voltzia*	Carbon film	-26.4126731			2024/7/30	CUG
	Pojiao	10-2-2	Surrounding rock	Mixed	-26.0421127			2024/7/30	CUG
	Pojiao	10-3-1	*Voltzia*	Carbon film	-25.1625522			2024/7/30	CUG
	Pojiao	10-3-2	Surrounding rock	Mixed	-26.0993622	-26.0754064		2024/7/30	CUG
	Pojiao	10-4-1	Stem	Woody	-26.7377749			2024/7/30	CUG
	Pojiao	10-4-2	Surrounding rock	Mixed	-26.4843016			2024/7/30	CUG
	Pojiao	10-5-1	*Taeniopteris*	Carbon film	-25.5674167			2024/7/30	CUG
	Pojiao	10-5-2	Surrounding rock	Mixed	-26.2815197			2024/7/30	CUG
	Pojiao	10-6-1	Root	Impression?	-26.4928224			2024/7/30	CUG
	Pojiao	10-6-2	Surrounding rock	Mixed	-26.2315565			2024/7/30	CUG
	Pojiao	10-7-1	Lycopod sporophyll leaf apex	Cuticle	-25.9786178			2024/7/30	CUG
	Pojiao	10-7-2	Surrounding rock	Mixed	-25.9057548			2024/7/30	CUG
	Pojiao	10-8-1	*Todites* stem	Woody	-25.508131			2024/7/30	CUG
	Pojiao	10-8-2	Surrounding rock	Mixed	-26.1024849			2024/7/30	CUG
	Pojiao	10-9-1	*Germaropteris* ovule cone	Carbon film	-25.1417342			2024/7/30	CUG
	Pojiao	10-9-2	Surrounding rock	Mixed	-25.737129			2024/7/30	CUG
	Lubei	11-1-1	Seed	Woody	-25.560176			2024/7/30	CUG
	Lubei	11-1-2	Surrounding rock	Mixed	-25.4758631			2024/7/30	CUG

	Lubei	11-2-1	*Germaropteris* stem	Woody	-26.1140011			2024/7/30	CUG
	Lubei	11-2-2	Surrounding rock	Mixed	-26.1045667			2024/7/30	CUG
	Lubei	11-3-1	*Voltzia*	Carbon film	-26.0254583			2024/7/30	CUG
	Lubei	11-3-2	Surrounding rock	Mixed	-26.7707427			2024/7/30	CUG
	Lubei	11-4-1	*Germaropteris* stem	Woody	-25.7322265			2024/7/30	CUG
	Lubei	11-4-2	Surrounding rock	Mixed	-26.5896261			2024/7/30	CUG
	Lubei	11-5-1	Stem	Woody	-23.1620281			2024/7/30	CUG
	Lubei	11-5-2	Surrounding rock	Mixed	-25.3884275			2024/7/30	CUG
	Lubei	11-6-1	*Neocalamites*	Carbon film	-25.362405			2024/7/30	CUG
	Lubei	11-6-2	Surrounding rock	Mixed	-26.049399			2024/7/30	CUG
	Pojiao	C1-1	Surrounding rock	Mixed	-25.480719	-25.500528		2025/4/21	UCD
Permian Triassic transition (Kayitou Formation)	Longmendong	3-3-1	*Germaropteris* leaf (*Lepidopteris*)	Cuticle	-30.08			2023/9/1	CUG
	Longmendong	3-3-2	Surrounding rock	Mixed	-28.58			2023/9/2	CUG
	Longmendong	3-4-1	Unknown leaf	Cuticle	-25.58			2023/9/3	CUG
	Jinjibang	5-1-1	*Tomiostrobus* sporophyll	Carbon film	-28.11			2023/10/6	CUG
	Jinjibang	5-1-2	Surrounding rock	Mixed	-28.06	-27.86		2023/10/7	CUG
	Feixianguan	WD-20	Putative *Germaropteris* stem	Mixed	-32.05			Li et al.[62]	CUG
	Chinahe	7-1-1	*Tomiostrobus*	Carbon film	-28.0343482			2024/7/30	CUG
	Chinahe	7-1-2	Surrounding rock	Mixed	-27.2474749			2024/7/30	CUG
	Chinahe	7-2-1	*Tomiostrobus*	Carbon film	-28.1374444	-28.058336		2024/7/30	CUG
	Chinahe	7-2-2	Surrounding rock	Mixed	-27.891792	-27.7620991		2024/7/30	CUG
	Chinahe	7-3-1	*Tomiostrobus*	Carbon film	-27.1810924			2024/7/30	CUG
	Chinahe	7-3-2	Surrounding rock	Mixed	-27.3630148	-27.1048716		2024/7/30	CUG
	Jiucaiyuan	8-1-1	*Tomiostrobus*	Carbon film	-26.5677496			2024/7/30	CUG
	Jiucaiyuan	8-1-2	Surrounding rock	Mixed	-26.5531946			2024/7/30	CUG
	Jiucaiyuan	8-2-1	*Tomiostrobus*	Carbon film	-26.684348			2024/7/30	CUG
	Jiucaiyuan	8-2-2	Surrounding rock	Mixed	-26.6250167			2024/7/30	CUG
	Jiucaiyuan	8-3-1	*Tomiostrobus*	Carbon film	-27.1652438			2024/7/30	CUG
	Jiucaiyuan	8-3-2	Surrounding rock	Mixed	-26.3054604			2024/7/30	CUG
	Jiucaiyuan	8-4-1	*Germaropteris* leaf (*Lepidopteris*)	Cuticle	-30.9696316	-30.8621923		2024/7/30	CUG

	Jiucaiyuan	8-4-2	Surrounding rock	Mixed	-27.9896366			2024/7/30	CUG
	Kele	9-1-1	*Tomiostrobus*	Carbon film	-28.3608385			2024/7/30	CUG
	Kele	9-1-2	Surrounding rock	Mixed	-28.2269618	-28.3539516		2024/7/30	CUG
	Guanbachong	L1-1	*Tomiostrobus*	Carbon film	-26.1913603			2025/4/21	UCD
	Guanbachong	L1-2	Surrounding rock	Mixed	-26.261338	-26.4567220		2025/4/21	UCD
	Guanbachong	L2-1	*Tomiostrobus*	Carbon film	-26.6637587			2025/4/21	UCD
	Guanbachong	L2-2	Surrounding rock	Mixed	-25.9712620	-25.9347019		2025/4/21	UCD
	Jiucaiyuan	F1-1	*Germaropteris* leaf (*Lepidopteris*)	Carbon film	-29.5165532			2025/4/21	UCD
	Jiucaiyuan	F1-2	Surrounding rock	Mixed	-27.6572962			2025/4/21	UCD
End Permian (Xuanwei and Dalong formations)	Longmendong	3-1	*Lepidodendron* trunk	Cuticle	-25.67			2023/5/17	CUG
	Longmendong	3-2	Surrounding rock	Mixed	-24.59			2023/5/18	CUG
	Chahe	CH-18-1	*Lepidodendron* trunk	Cuticle	-24.16			Li et al.[17]	CUG
	Chahe	CH-18-2	*Lepidodendron* trunk	Cuticle	-23.35			Li et al.[17]	CUG
	Chahe	CH-18-3	*Pecopteris*	Carbon film	-23.48			Li et al.[17]	CUG
	Chahe	CH-69-1	Unknown leaf	Carbon film	-26.9			Li et al.[17]	CUG
	Chahe	CH-69-2	*Cordaites*	Carbon film	-25.17			Li et al.[17]	CUG
	Jiucaichong	JC-24	*Cordaites*	Carbon film	-24.88			Li et al.[17]	CUG
	Wadu	WD-2	Unknown trunk	Cuticle	-23.65			Li et al.[17]	CUG
	Wadu	WD-8	Unknown trunk	Cuticle	-24.07			Li et al.[17]	CUG
	Kejiao	KJ-1	*Anshuncladus*	Cuticle	-26.65			Li et al.[17]	CUG
	Kejiao	KJ-2-1	*Szecladia*	Cuticle	-24.32			Li et al.[17]	CUG
	Kejiao	KJ-2-2	*Szecladia*	Cuticle	-24.29			Li et al.[17]	CUG
	Kejiao	KJ-3-1	*Anshuncladus*	Cuticle	-23.47			Li et al.[17]	CUG
	Kejiao	KJ-3-2	*Szecladia*	Cuticle	-25.57			Li et al.[17]	CUG
	Kejiao	KJ-7	*Szecladia*	Cuticle	-24.41			Li et al.[17]	CUG
	Kejiao	KJ-11-1	*Pseudoullmannia*	Cuticle	-24.65			Li et al.[17]	CUG
	Kejiao	KJ-11-2	*Taeniopteris*	Cuticle	-24.41			Li et al.[17]	CUG
	Kejiao	KJ-11-3	*Taeniopteris*	Cuticle	-24.76			Li et al.[17]	CUG
	Kejiao	KJ-13	*Anshuncladus*	Cuticle	-23.69			Li et al.[17]	CUG
	Kejiao	KJ-17	*Anshuncladus*	Cuticle	-23.5			Li et al.[17]	CUG

	Duanshan A	DSA3-1-1	*Anshuncladus*	Cuticle	-26.1			Li et al.[17]	CUG
	Duanshan A	DSA3-1-2	*Anshuncladus*	Cuticle	-24.38			Li et al.[17]	CUG
	Duanshan A	DSA3-1-3	*Anshuncladus*	Cuticle	-23.89			Li et al.[17]	CUG
	Duanshan A	DSA3-1-4	*Taeniopteris*	Cuticle	-25.06			Li et al.[17]	CUG
	Duanshan A	DSA3-1-5	*Taeniopteris*	Cuticle	-25.02			Li et al.[17]	CUG
	Duanshan A	DSA3-2	*Taeniopteris*	Cuticle	-24.45			Li et al.[17]	CUG
	Duanshan A	DSA3-3	*Anshuncladus*	Cuticle	-23.29			Li et al.[17]	CUG
	Duanshan A	DSA3-4-1	*Anshuncladus*	Cuticle	-24.68			Li et al.[17]	CUG
	Duanshan A	DSA3-4-2	*Anshuncladus*	Cuticle	-23.93			Li et al.[17]	CUG
	Duanshan A	DSA3-4-3	*Anshuncladus*	Cuticle	-24.11			Li et al.[17]	CUG
	Duanshan A	DSA3-4-4	*Taeniopteris*	Cuticle	-24.45			Li et al.[17]	CUG
	Duanshan A	DSA3-5-1	*Anshuncladus*	Cuticle	-24.85			Li et al.[17]	CUG
	Duanshan A	DSA3-5-2	*Anshuncladus*	Cuticle	-24.42			Li et al.[17]	CUG
	Duanshan A	DSA5	*Anshuncladus*	Cuticle	-25.57			Li et al.[17]	CUG
	Duanshan B	DSB2-1-1	*Anshuncladus*	Cuticle	-25.92			Li et al.[17]	CUG
	Duanshan B	DSB2-1-2	*Anshuncladus*	Cuticle	-24.23			Li et al.[17]	CUG
	Duanshan B	DSB2-1-3	*Anshuncladus*	Cuticle	-24.58			Li et al.[17]	CUG
	Duanshan B	DSB2-1-4	*Anshuncladus*	Cuticle	-24.7			Li et al.[17]	CUG
	Duanshan B	DSB2-1-5	*Anshuncladus*	Cuticle	-25.39			Li et al.[17]	CUG
	Duanshan B	DSB2-1-6	*Anshuncladus*	Cuticle	-23.62			Li et al.[17]	CUG
	Duanshan B	DSB2-1-7	*Anshuncladus*	Cuticle	-25.81			Li et al.[17]	CUG
	Duanshan B	DSB2-1-8	*Anshuncladus*	Cuticle	-24.05			Li et al.[17]	CUG
	Duanshan B	DSB2-1-9	*Anshuncladus*	Cuticle	-25.8			Li et al.[17]	CUG
	Duanshan B	DSB2-1-10	*Anshuncladus*	Cuticle	-25.82			Li et al.[17]	CUG
	Duanshan B	DSB2-2-1	*Anshuncladus*	Cuticle	-25.13			Li et al.[17]	CUG
	Duanshan B	DSB2-2-2	*Anshuncladus*	Cuticle	-24.55			Li et al.[17]	CUG
	Duanshan B	DSB2-2-3	*Taeniopteris*	Cuticle	-25.97			Li et al.[17]	CUG
	Duanshan B	DSB2-2-4	*Taeniopteris*	Cuticle	-25.72			Li et al.[17]	CUG
	Duanshan B	DSB5-1-1	*Anshuncladus*	Cuticle	-24.87			Li et al.[17]	CUG
	Duanshan B	DSB5-1-2	*Taeniopteris*	Cuticle	-24.02			Li et al.[17]	CUG
	Duanshan B	DSB6	*Anshuncladus*	Cuticle	-25.38			Li et al.[17]	CUG

R code 1 is for figure S2 and S3

```
#This code is for plotting the morphometrics PCA with centroid and data point name
#Please acknowledge Zhen Xu if it's helpful, for further question, email Z.xu@leeds.ac.uk
library(ggplot2)
library(ggrepel)
library(factoextra)
df <- read.csv("D:/PCA-centroid test/Pleuromeia/R/Pleuromeia.csv", header=T)
rownames(df)=df[,70]
head(df)
pca1<-prcomp(df[,1:68],center = T, scale. = T)
summary(pca1)
fviz_eig(pca1,addlabels = T)
fviz_pca_ind(pca1, axes = c(1, 2), col.ind = df$group, palette c("#FFFF66","#FFB6C1", "#9400D3","#0000FF","#B0C4DE","#808000","#006400","#BDB76B", "#FFA500", "#8B4513","#FF0000","#808080","#000000","#00FFFF","#32CD32","#FF00FF"), pointshape=21, addEllipses = T, ellipse.type = "convex", legend.title = "Groups", repel = TRUE)
#If the point name is too long and can't see it clear, change the figure size (suggest 1000mm×1000mm) when export the image
```

R code for figure S7

```
#This code is for plotting South China lycophyte sporophyll morphometrics (Triassic–Recent) PCA evaluated against sedimentary facies, and evaluates facies effects using PERMANOVA with a betadisper homogeneity check
#Please acknowledge Zhen Xu if it's helpful, for further question, email Z.xu@leeds.ac.uk
library(readxl)
library(tidyverse)
library(ggrepel)
library(vegan)
library(cluster)
library(ape)
file_path <- "D:/Data/2020.10.30-巴东文章/2022.5 新增/2023.9.19 manuscript/第八稿 2025.08.05/R 分析/看沉积相对形态矩阵影响.xls"
df_raw <- read_excel(file_path, sheet = 1)
nm <- names(df_raw)
pick_first <- function(cands) {
  hit <- cands[cands %in% nm]
  if (length(hit) == 0) NA_character_ else hit[1]
}
col_species <- pick_first(c("Species","Taxon","种名","属种","Content description","Genus species"))
col_facies  <- pick_first(c("Sedimentary facies","Facies","沉积相"))
if (is.na(col_species) || is.na(col_facies)) {
  stop("找不到‘种名/属种’或‘沉积相’列")
}
fac_raw <- as.character(df_raw[[col_facies]])
if (all(na.omit(unique(fac_raw)) %in% c("1","2","3","4"))) {
  fac_map <- c("1"="Marine","2"="Lagoon","3"="Delta & coastal plain","4"="Fluvial / Lacustrine")
```

```
  fac_raw <- unname(fac_map[fac_raw])
}
fac_low <- tolower(fac_raw)
fac_raw[grepl("^marin",        fac_low)] <- "Marine"
fac_raw[grepl("lagoon",        fac_low)] <- "Lagoon"
fac_raw[grepl("delta|coastal",  fac_low)] <- "Delta & coastal plain"
fac_raw[grepl("fluvial|lacus|river|lake", fac_low)] <- "Fluvial / Lacustrine"
fac_raw[trimws(fac_raw) == ""] <- NA
df_raw[[col_facies]] <- forcats::fct_explicit_na(
  factor(fac_raw, levels = c("Marine","Lagoon","Delta & coastal plain","Fluvial / Lacustrine")),
  na_level = "Unknown facies"
)
print(table(df_raw[[col_facies]], useNA = "ifany"))
meta_cols <- c(col_species, col_facies,
               intersect(c("Section number","Age","Sample number","Carbon type",
                           "δ13Corg","Test time","Test location"), nm))
morph_cols <- setdiff(nm, meta_cols)
morph <- df_raw[, morph_cols, drop = FALSE]
to_numeric_safe <- function(x){
  if (is.numeric(x)) return(x)
  if (is.logical(x)) return(as.numeric(x))
  v <- as.character(x)
  yn_map                                                                              <-
c("Y"=1,"N"=0,"Yes"=1,"No"=0,"YES"=1,"NO"=0,"y"=1,"n"=0,"T"=1,"F"=0,"True"=1,"Fal
se"=0)
  if (all(na.omit(v) %in% names(yn_map))) return(as.numeric(unname(yn_map[v])))
  suppressWarnings(vn <- as.numeric(v))
  if (mean(is.na(vn)) < 0.5) return(vn)
  rep(NA_real_, length(v))
}
morph_num <- as.data.frame(lapply(morph, to_numeric_safe))
keep_cols <- names(morph_num)[colSums(!is.na(morph_num)) > 0]
morph_num <- morph_num[, keep_cols, drop = FALSE]
if (ncol(morph_num) < 2) stop("有效的数值化形态特征列少于 2 列，无法做 PCA")
for (j in seq_along(morph_num)) {
  col <- morph_num[[j]]
  if (anyNA(col)) col[is.na(col)] <- median(col, na.rm = TRUE)
  morph_num[[j]] <- col
}
morph_scaled <- scale(morph_num)
pca_res <- prcomp(morph_scaled, center = TRUE, scale. = FALSE)
var_expl <- (pca_res$sdev^2) / sum(pca_res$sdev^2)
pc1_var <- round(100 * var_expl[1], 1)
pc2_var <- round(100 * var_expl[2], 1)
scores <- as.data.frame(pca_res$x[, 1:2])
names(scores)[1:2] <- c("PC1","PC2")
scores$Species <- as.factor(df_raw[[col_species]])
scores$Facies  <- df_raw[[col_facies]]
centroids <- scores %>%
  group_by(Species) %>% summarise(PC1 = mean(PC1), PC2 = mean(PC2), .groups = "drop")
```

```
fac_levels <- levels(scores$Facies)
shape_values <- setNames(rep(c(16, 17, 15), length.out = length(fac_levels)), fac_levels)

p <- ggplot(scores, aes(PC1, PC2, shape = Facies, color = Species)) +
  geom_point(size = 2.6, alpha = 0.95) +
  geom_point(data = centroids, aes(PC1, PC2), inherit.aes = FALSE, size = 0, alpha = 0) +
  ggrepel::geom_text_repel(data = centroids, aes(PC1, PC2, label = Species),
                           inherit.aes = FALSE, size = 3, max.overlaps = 60,
                           box.padding = 0.3, point.padding = 0.2, min.segment.length = 0) +
  scale_shape_manual(values = shape_values) +
  guides(
    shape = guide_legend(title = "Sedimentary facies", override.aes = list(size = 3)),
    color = guide_legend(title = "Species",         override.aes = list(shape = 18, size = 3))
  ) +
  labs(
    title = "Morphological PCA — shape by facies, color by species",
    x = paste0("PC1 (", pc1_var, "%)"),
    y = paste0("PC2 (", pc2_var, "%)")
  ) +
  theme_minimal(base_size = 13) +
  theme(panel.grid.minor = element_blank(),
        legend.position = "right")
print(p)
dist_euc <- dist(morph_scaled, method = "euclidean")
perm <- adonis2(dist_euc ~ scores$Facies, permutations = 999)
cat("\nPERMANOVA (Euclidean, scaled morph):\n"); print(perm)
bd <- betadisper(dist_euc, group = scores$Facies)
cat("\nHomogeneity of multivariate dispersion (betadisper) — ANOVA:\n")
print(anova(bd))
```

R code for figure S8

```
#This code is for plotting the δ13Corg by sedimentary facies for late Permian to Middle Triassic lycophyte and non-lycophyte plants
#Please acknowledge Zhen Xu if it's helpful, for further question, email Z.xu@leeds.ac.uk
library(readxl)
library(tidyverse)
library(ggrepel)
file_path <- "D:/Data/2020.10.30-巴东文章/2022.5 新增/2023.9.19 manuscript/第八稿 2025.08.05/R分析/看沉积相和碳同位素和形态矩阵.xls"
df_raw <- read_excel(file_path, sheet = 1)
df <- df_raw %>%
  transmute(
    Age     = as.factor(`Age`),
    Facies = as.character(`Sedimentary facies`),
    Taxon  = as.factor(`Content description`),
    delta13C = suppressWarnings(as.numeric(`δ13Corg`))
  ) %>%
  filter(is.finite(delta13C))
df <- df %>%
```

```
  mutate(
    Facies = recode(Facies,
                    "1" = "Marine",
                    "2" = "Lagoon",
                    "3" = "Delta & coastal plain",
                    "4" = "Fluvial / Lacustrine"),
    Facies = factor(Facies,
                    levels = c("Marine","Lagoon","Delta & coastal plain","Fluvial / Lacustrine"))
  )
p <- ggplot(df, aes(x = delta13C, y = Facies,
                    color = Taxon, shape = Age)) +
  geom_point(size = 2.8,
             position = position_jitter(height = 0.12, width = 0)) +
  scale_x_continuous(expand = expansion(mult = c(0.05, 0.05))) +
  labs(
    x = expression(delta^{13}*C[org]~"\u2030"),
    y = "Sedimentary facies",
    color = "Taxon",
    shape = "Age",
    title = expression("Distribution of " * delta^{13}*C[org] * " by facies")
  ) +
  theme_minimal(base_size = 13) +
  theme(
    panel.grid.minor = element_blank(),
    legend.position = "right",
    legend.key.height = unit(0.9, "lines")
  )

print(p)
```

Supplementary references:

77. Xu, Z. et al. Normalization of fossil plant megafossil databases for diversity and palaeobiogeography analyses by filtering taxonomic duplication: Principles, methods, examples, and recommendations. *Palaeogeogr. Palaeoclimat. Palaeoecol.* p.113236 (2025).
78. Chu, D. et al. Biostratigraphic correlation and mass extinction during the Permian-Triassic transition in terrestrial-marine siliciclastic settings of South China. *Glob. Planet. Change* 146, 67–88 (2016).
79. Zhou, Z. Y. & Li, B. X. A preliminary study of the Early Triassic plants from the Qionghai District, Hainan Island. *Acta Palaeontol. Sin.* 18, 444–464 (1979).
80. Wang, Z. & Lou, Y. Studies on the Megaspores of a Mid-Triassic Lycopsid in Shaanxi,China. J. Integr. Plant Biol. 32, (1990).
81. Ye, M. On some Middle Triassic plants from Hupeh and Szechuan. *Acta Palaeontol. Sin.* 18, 73–82 (1979).
82. Emmons, E. Geological Report of the Midland Countries, North Carolina. (Putnam, New York, 1856).
83. Fliche, P. & Zeiller, R. Flore fossile du trias en Lorraine et Franche-comté: avec des considérations finales. Berger-Levrault, éditeurs (1910).

84. Neuburg, M. F. *Pleuromeia* Corda from the Lower Triassic deposits of the Russian platform. *Trudy Geologičeskiego Instituta Akademii Nauk* S.S.S.R. 43, 65–94 (1960).
85. Grauvogel-Stamm, L. & Duringer, P. Annalepis zeilleri Fliche 1910 emend., un organe reproducteur de Lycophyte de la Lettenkohle de l'Est de la France. Morphologie, spores in situ et paléoécologie. *Geologische Rundschau* 72, 23–51 (1983).
86. Wang, Z. Advances on the Permo-Triassic lycopods in North China. I. An *Isoetes* from the mid-Triassic in northern Shaanxi province. in Palaeontographica Abteilung B Paläophytologie 1–30 (Publication country Germany, Stuttgart, 1991).
87. Meng, F. Studies on Annalepis from Middle Triassic Along the Yangtze River and Its Bearing on the Origin of *Isoetes*. *Acta Bot. Sin.* 8, 768–774 (1998).
88. Meng, F. S. Floral palaeoecological environment of the Badong Formation in the Yangtze gorges area. *Geology Mineral Res.* South China 1–13 (1996).
89. Meng, F. S. The *Annalepis-Pleuromeia* plant assemblage in South China and the significance of it. Chinese Sci. Bull. 18, 1686–1688 (1993).
90. Meng, F. Studies on *Annalepis* from Middle Triassic along the Yangtze river and its bearing on the origin of the *Isoetes*. *Acta Bot. Sin.* 40, 768–774 (1998).
91. Kustatscher, E., Wachtler, M. & Van Konijnenburg-Van Cittert, J. H. A. Lycophytes from the Middle Triassic (Anisian) locality Kühwiesenkopf (Monte Prà della Vacca) in the Dolomites (northern Italy). *Palaeontology* 53, 595–626 (2010).
92. Wang, Z. Advances on the Permo-Triassic lycopods in North China. I. An *Isoetes* from the Mid-Triassic in northern Shannxi Province. *Palaeontographica* 222, 1–30 (1991).
93. Bauer, K. et al. *Lepacyclotes kirchneri* n. sp.(Isoetales, Isoetaceae) aus dem unteren Jura von Oberfranken, Deutschland. *Berichte der Naturwissenschaftlichen Gesellschaft Bayreuth* 27, 429–443 (2015).
94. Brik, M. I. Iskopaemaya flora i stratigraphiya niznemezozoiskikh otlozhenii basseina srednego R. Ilek v zapadnom kazachstane (Fossil flora and stratigraphy of lower Mezozoic deposits of the central Ilek River in western Kazachstan). *Trudy Geologiskhaya Instituta Nauchotekhniskoi Akademia Nauk S.S.S.R.* 9, 3–73 (1952).
95. Sadovnikov, G. N. The morphology, systematics and distribution of the genus *Tomiostrobus*. *Paleontological J.* 1, 100–109 (1982).
96. Bauer, K., Kustatscher, E. & Krings, M. The ginkgophytes from the German Kupferschiefer (Permian), with considerations on the taxonomic history and use of Baiera and Sphenobaiera. *Bull. Geosci.* 88, 539–556 (2013).
97. Snigirevskaya, S. N. Nakhodka novogo iskopaemogo roda isostovikh v rannetriasovikh otlozheniyakh vostochnoi sibiri (A new fossil genus of Isoetopsida in the Triassic of eastern Siberia). *Bot. Z.* 65, 95–96 (1980).
98. Lundblad. A selaginelloid strobilus from East Greenland (Triassic). *Meddelelser Danske Geologische Foreningens* 11, 351–363 (1948).
99. Dobruskina, I. A. Some problems of the systematics of Triassic Lepidophytes. *Paleontol. J.* 19, 74–88 (1985).
100. Ash, S. R. *Skilliostrobus* gen. nov., a new lycopsid cone from the Early Triassic of Australia. *Alcheringa: An Australasian J. Palaeontol.* 3, 73–89 (1979).
101. Ash, S. R. & Pigg, K. B. A new Jurassic *Isoetites* (Isoetales) from the Wallowa Terrane in Hells Canyon, Oregon and Idaho. *Am. J. Bot.* 78, 1636–1642 (1991).
102. Prada, C. & Rolleri, C. H. A new species of Isoetes (Isoetaceae) from Turkey, with a study of microphyll intercellular pectic protuberances and their potential taxonomic value. Bot. J. Linnean Soc.147, 213–228 (2005).
103. Qin, R., Wang, Y., Yan, C. & Liu. H. Anatomical Structure of Sporophylls of Critically Endangered Plant Isoetes sinensis Palmer. *J. South-Central University for Nationalities (Nat. Sci. Edition)* 28, 38–41 (2009).

104. Skog, J. E. & Hill, C. R. Mesozoic lycopods. *Annals Misouri Bot. Garden* 79, 648–675 (1992).
105. Teixeira, C. Flora Mesozóica Portuguesa. (Alexander Doweld, 1948).
106. Hickey, J. R. The early evolutionary and morphological diversity of *Isoetes*, with descriptions of two new Neotropical species. *Syst. Bot.* 11, 309–321 (1986).
107. Barale, G. Sur la présence d'une nouvelle espèce d' Isoetites dans la flore du Crétacé inférieur de la région de Tataouine (Sud tunisien) : implications paléoclimatiques et phylogénétiques. *Canadian J. Bot.* 77, 189–196 (1999).
108. Brown, R. W. Some American fossil plants belonging to the Isoetales. *J. Washington Acad. Sci.* 29, 261 (1939).
109. Villanueva-Amadoz, U. A new isoetalean microsporophyll from the latest Albian of northeastern Spain: Diversity in the development and dispersal strategies of microspores. *Acta Palaeontol. Pol.* (2012) doi:10.4202/app.2012.0010.
110. Pereira, J. B. D. S., Salino, A., Arruda, A. & Stutzel, T. Two new species of *Isoetes* (Isoetaceae) from northern Brazil. Phytotaxa 272, 141 (2016).
111. Liu, H., Wang, Q. F. & Taylor, W. C. Morphological and anatomical variation in sporophylls of *Isoetes sinensis* palmer (Isoetaceae), an endangered quillwort in China. *Am. Fern. J.* 96, 67–74 (2006).
112. Naugolnykh, S. V. & Mogutcheva, N. K. A new representative of *Isoetes* (Lycopodiopsida) from the lower Triassic of the Tunguska Basin. *Новости* 8, 81–93 (2006).
113. Brunton, D. F. & Britton, D. M. The Status, Distribution, and Identification of Georgia Quillwort (*Isoetes georgiana*; Isoetaceae). *Am. Fern. J* 86, 105 (1996).
114. Wang, L. X., Xie, Z. M. & Wang, Z. Q. On the occurrence of *Pleuromeia* from the Qinshui Basin in Shanxi province. *Acta Palaeontol. Sinica* 17, 195–212 (1978).
115. Deng, S. et al. A new species of *Pleuromeia* (Lycopsid) from the upper Middle Triassic of Northern China and discussion on the spatiotemporal distribution and evolution of the genus. *Geobios* 75, 1–15 (2022).
116. Lugardon, B., Grauvogel-Stamm, L. & Dobruskina, I. The microspores of *Pleuromeia rossica* Neuburg (Lycopsida; Triassic): Comparative ultrastructure and phylogenetic implications. *Comptes Rendus de l'Académie des Sciences - Series IIA – Earth Planet. Sci*. 329, 435–442 (1999).
117. Grauvogel-Stamm, L. Pleuromeia sternbergii (Münster) Corda from the Lower Triassic of Germany—further observations and comparative morphology of its rooting organ. *Rev. Palaeobot. Palynol.* 77, 185–212 (1993).
118. Bomfleur, B., Krings, M., Taylor, E. L. & Taylor, T. N. Macrofossil Evidence for Pleuromeialean Lycophytes from the Triassic of Antarctica. *Acta. Palaeontol. Pol.* 56, 195–203 (2011).
119. Zhang, Y., Wang, Y. D., Hong, Y., Cao, L. & Gao, F. liang. *Pleuromeia* discovered from the Middle Triassic Linjia Formation of Benxi, Northeast China. *Palaeoworld* 29, 706–714 (2020).
120. Lele, K. M. Studies in the Indian Middle Gondwana flora-2. Plant fossils from the South Rewa Gondwana Basin. *Palaeobotanist* 10, 69–83 (1962).
121. Helby, R. & Martin, R. H. *Cylostrobus* gen. nov., cones of lycopsidean plants from the Narrabeen Group (Triassic) of New South Wales. *Aust. J. Bot.* 13, 389–404 (1965).
