# Supplementary material for: CAM photosynthesis may have conferred an advantage during the Permian–Triassic mass extinction event
Source: Nat Ecol Evol. 2026 Apr 20;10(5):997–1010. doi: 10.1038/s41559-026-03026-0 (PMC13167456; doi:10.1038/s41559-026-03026-0)
Supplement: Supplementary file 2 — Reporting Summary [file 41559_2026_3026_MOESM2_ESM.pdf]

nature portfolio

Corresponding author(s): Zhen Xu, Jianxin Yu

Last updated by author(s): Jan 15, 2026

Reporting Summary

Nature Portfolio wishes to improve the reproducibility of the work that we publish. This form provides structure for consistency and transparency in reporting. For further information on Nature Portfolio policies, see our Editorial Policies and the Editorial Policy Checklist.

Statistics

For all statistical analyses, confirm that the following items are present in the figure legend, table legend, main text, or Methods section.

n/a	Confirmed	
☐	☒	The exact sample size (n) for each experimental group/condition, given as a discrete number and unit of measurement
☐	☒	A statement on whether measurements were taken from distinct samples or whether the same sample was measured repeatedly
☒	☐	The statistical test(s) used AND whether they are one- or two-sided<br>*Only common tests should be described solely by name; describe more complex techniques in the Methods section.*
☒	☐	A description of all covariates tested
☒	☐	A description of any assumptions or corrections, such as tests of normality and adjustment for multiple comparisons
☒	☐	A full description of the statistical parameters including central tendency (e.g. means) or other basic estimates (e.g. regression coefficient) AND variation (e.g. standard deviation) or associated estimates of uncertainty (e.g. confidence intervals)
☒	☐	For null hypothesis testing, the test statistic (e.g. F, t, r) with confidence intervals, effect sizes, degrees of freedom and P value noted<br>*Give P values as exact values whenever suitable.*
☒	☐	For Bayesian analysis, information on the choice of priors and Markov chain Monte Carlo settings
☒	☐	For hierarchical and complex designs, identification of the appropriate level for tests and full reporting of outcomes
☒	☐	Estimates of effect sizes (e.g. Cohen's d, Pearson's r), indicating how they were calculated

Our web collection on statistics for biologists contains articles on many of the points above.

Software and code

Policy information about availability of computer code

Data collection	Open access software: ImageJ
Data analysis	Open access software: PAST, Mesquite, PAUP, SpiltTree, Matlab, R

For manuscripts utilizing custom algorithms or software that are central to the research but not yet described in published literature, software must be made available to editors and reviewers. We strongly encourage code deposition in a community repository (e.g. GitHub). See the Nature Portfolio guidelines for submitting code & software for further information.

Data

Policy information about availability of data

All manuscripts must include a data availability statement. This statement should provide the following information, where applicable:

- Accession codes, unique identifiers, or web links for publicly available datasets
- A description of any restrictions on data availability
- For clinical datasets or third party data, please ensure that the statement adheres to our policy

The data that support the findings of this study are included in the paper and/or the supplementary information.

Research involving human participants, their data, or biological material

Policy information about studies with human participants or human data. See also policy information about sex, gender (identity/presentation), and sexual orientation and race, ethnicity and racism.

Reporting on sex and gender	*Use the terms sex (biological attribute) and gender (shaped by social and cultural circumstances) carefully in order to avoid confusing both terms. Indicate if findings apply to only one sex or gender; describe whether sex and gender were considered in study design; whether sex and/or gender was determined based on self-reporting or assigned and methods used. Provide in the source data disaggregated sex and gender data, where this information has been collected, and if consent has been obtained for sharing of individual-level data; provide overall numbers in this Reporting Summary. Please state if this information has not been collected. Report sex- and gender-based analyses where performed, justify reasons for lack of sex- and gender-based analysis.*
Reporting on race, ethnicity, or other socially relevant groupings	*Please specify the socially constructed or socially relevant categorization variable(s) used in your manuscript and explain why they were used. Please note that such variables should not be used as proxies for other socially constructed/relevant variables (for example, race or ethnicity should not be used as a proxy for socioeconomic status). Provide clear definitions of the relevant terms used, how they were provided (by the participants/respondents, the researchers, or third parties), and the method(s) used to classify people into the different categories (e.g. self-report, census or administrative data, social media data, etc.) Please provide details about how you controlled for confounding variables in your analyses.*
Population characteristics	*Describe the covariate-relevant population characteristics of the human research participants (e.g. age, genotypic information, past and current diagnosis and treatment categories). If you filled out the behavioural & social sciences study design questions and have nothing to add here, write "See above."*
Recruitment	*Describe how participants were recruited. Outline any potential self-selection bias or other biases that may be present and how these are likely to impact results.*
Ethics oversight	*Identify the organization(s) that approved the study protocol.*

Note that full information on the approval of the study protocol must also be provided in the manuscript.

Field-specific reporting

Please select the one below that is the best fit for your research. If you are not sure, read the appropriate sections before making your selection.

☐ Life sciences ☐ Behavioural & social sciences ☒ Ecological, evolutionary & environmental sciences

For a reference copy of the document with all sections, see nature.com/documents/nr-reporting-summary-flat.pdf

Ecological, evolutionary & environmental sciences study design

All studies must disclose on these points even when the disclosure is negative.

Study description	This study investigates the survival strategies of lycophyte plants across the Permian–Triassic Mass Extinction (PTME), with a focus on whether Crassulacean Acid Metabolism (CAM) photosynthesis enabled their persistence under extreme environmental stress. The study combines phylogenetic analysis, morphological quantification (127 traits), carbon isotope geochemistry, and Earth system climate modeling (HadCM3BL). It uses a factorial design integrating multiple methods across time intervals (Late Permian to Middle Triassic) and geographic regions.
Research sample	The research sample consists of 485 lycophyte sporophyll fossil specimens from the Late Permian to Middle Triassic of South China and other global sites, along with 200 literature-derived fossil data points. The focus is on sporophylls, as these reproductive structures preserve the most diagnostic characters. The sample includes both extinct taxa and extant Isoetes species to assess phylogenetic and ecophysiological continuity. Specimens were collected from fieldwork and literature and were selected based on preservation quality and stratigraphic context.
Sampling strategy	No formal statistical power calculation was performed due to fossil record constraints. Sample sizes were determined based on fossil availability and completeness, with efforts to maximize taxonomic, stratigraphic, and geographic coverage. Sampling aimed to include all morphologically character-rich sporophylls suitable for morphometric and isotopic analysis. Carbon isotope samples pooled material from the same species, locality, and stratigraphic level to ensure data quality.
Data collection	Morphological data were scored from fossils collected in this work and also references using a 127-character matrix and visualized via PCA and Neighborhood Network. Analysis Isotope data were collected from the cuticle-bearing surfaces of plant fossils collected using a scalpel, avoiding matrix contamination. All the fossils are cleaned using compressed before sampling. Data were recorded and processed by the lead author and collaborators at the University of Leeds and China University of Geosciences Wuhan. Climate model simulations were run by a co-author using HadCM3BL under different CO_2 scenarios, which have been previously published.
Timing and spatial scale	Fossil data span ~254–237 Ma, covering the Changhsingian, Permian–Triassic transition, and Early–Middle Triassic. Fossils used for phylogeny were collected from multiple sedimentary facies across South China, in field work with additional data from North China, China Xinjiang, Europe, Australia, Russia, middle Asia, Antarctica, Argentina in references. The carbon isotope data only come from

	South China because South China was in the low latitude tropical area thus has the highest temperature and highest potential of CAM. The climate simulations are come from a published paper (Sun et al., 2024, Science), and the full simulations can be found in the Bristol BRIDGE website (https://www.bristol.ac.uk/geography/research/bridge/).
Data exclusions	Fossils lacking key morphological features or too poorly preserved for confident character coding were excluded. Similarly, literature-derived isotope data without associated sediment $\delta^{13}C$ values were excluded from the core analysis. The rationale for all exclusions is described in the Methods and Supplementary Information. No exclusions were based on results.
Reproducibility	All data, including morphological matrices, specimen photographs, images of carbon isotope sampling sites on both fossils and rocks, and measured $\delta^{13}C$ values, are provided in the Supplementary Information. PCA and NNA procedures are described in full detail with annotated figures, and all software used (PAST, Mesquite, PAUP, SplitTree, and R) is freely available. These methods can therefore be readily applied to other fossil datasets. Carbon isotope measurements were independently conducted at the China University of Geosciences (Wuhan) and the University of California, Davis, using three different sets of instruments and standards, yielding consistent results. Remaining isotope samples are archived at the University of Leeds. Climate model simulations are reproducible using the HadCM3BL code and settings described in the cited references, and code availability is provided. For figures containing small symbols that cannot be enlarged due to journal size constraints, we have included a statement noting that all source data and code are available in the Supplementary Information, enabling others to reproduce the analyses and generate high-resolution vector figures.
Randomization	Randomization is not applicable, as this is a retrospective fossil-based study. Sample selection was determined by preservation quality, stratigraphic control, and specimen completeness, not experimental manipulation. Covariates such as geography, facies, and age were recorded for all samples and considered in interpretation.
Blinding	Blinding was not relevant to this study. Data acquisition and analysis involved fossil specimens and geochemical measurements that are not subject to observer bias in the conventional sense. Morphological character scoring followed predefined criteria, and isotopic analyses were performed using standardized laboratory protocols. Sample selection was based on preservation quality and stratigraphic control, not experimental grouping, making blinding unnecessary.

Did the study involve field work? ☒ Yes ☐ No

Field work, collection and transport

Field conditions	The field works are conducted during summer in South China when the highest day temperature is below 35 degree and we only work when there is no rain to avoid potential risks.
Location	The field works are in Hubei, Hunan, Guizhou, Yunnan, Sichuan provinces in South China. The carbon isotope experiments are taken in the China University of Geosciences Wuhan and University of California Davis.
Access & import/export	Fossil sampling was authorized by an official introduction letter from China University of Geosciences (Wuhan), which permitted access to fossil localities and collection in compliance with Chinese national regulations. The carbon isotope samples transported to University of Californian Davis were accompanied by an official academic invitation letter and a written explanation of research purpose.
Disturbance	This study caused minimal disturbance. All specimens were collected from previously excavated or naturally exposed outcrops, avoiding any protected or ecologically sensitive areas. No living ecosystems or habitats were altered. Sampling was conducted by hand without the use of heavy machinery.

Reporting for specific materials, systems and methods

We require information from authors about some types of materials, experimental systems and methods used in many studies. Here, indicate whether each material, system or method listed is relevant to your study. If you are not sure if a list item applies to your research, read the appropriate section before selecting a response.

Materials & experimental systems

n/a	Involved in the study	
☒	☐	Antibodies
☒	☐	Eukaryotic cell lines
☐	☒	Palaeontology and archaeology
☒	☐	Animals and other organisms
☒	☐	Clinical data
☒	☐	Dual use research of concern
☒	☐	Plants

Methods

n/a	Involved in the study	
☒	☐	ChIP-seq
☒	☐	Flow cytometry
☒	☐	MRI-based neuroimaging

Palaeontology and Archaeology

Specimen provenance	Fossil specimens were collected under an official introduction letter issued by China University of Geosciences (Wuhan), which permitted access and collection at designated fossil localities across Hubei, Hunan, Guizhou, Yunnan, and Sichuan provinces in China. Carbon isotope samples were transported to the University of California, Davis under an academic invitation and collaboration agreement for non-commercial scientific research. All specimens were collected in accordance with institutional and national regulations at the time of sampling.
Specimen deposition	All specimens and remaining samples are permanently stored at the China University of Geosciences (Wuhan), Room 014B of the Main Building, and at the University of Leeds, School of Earth and Environment. These repositories allow access to qualified researchers upon request.
Dating methods	No new radiometric or calibrated dates were produced in this study. Stratigraphic age assignments for the fossil specimens are based on previously published biostratigraphy and lithostratigraphy of the Kayitou, Feixianguan, and Badong formations. Full details and references are provided in the Supplementary Information and main text.

☒ Tick this box to confirm that the raw and calibrated dates are available in the paper or in Supplementary Information.

Ethics oversight	No ethical approval was required for this study, as it did not involve living organisms or human subjects. All fossil material was collected and studied in accordance with institutional and national guidelines for palaeontological research.

Note that full information on the approval of the study protocol must also be provided in the manuscript.

Plants

Seed stocks	*Report on the source of all seed stocks or other plant material used. If applicable, state the seed stock centre and catalogue number. If plant specimens were collected from the field, describe the collection location, date and sampling procedures.*
Novel plant genotypes	*Describe the methods by which all novel plant genotypes were produced. This includes those generated by transgenic approaches, gene editing, chemical/radiation-based mutagenesis and hybridization. For transgenic lines, describe the transformation method, the number of independent lines analyzed and the generation upon which experiments were performed. For gene-edited lines, describe the editor used, the endogenous sequence targeted for editing, the targeting guide RNA sequence (if applicable) and how the editor was applied.*
Authentication	*Describe any authentication procedures for each seed stock used or novel genotype generated. Describe any experiments used to assess the effect of a mutation and, where applicable, how potential secondary effects (e.g. second site T-DNA insertions, mosiacism, off-target gene editing) were examined.*
